# The archaeological evidence for the appearance of pastoralism and farming in southern Africa

**DOI:** 10.1371/journal.pone.0198941

**Published:** 2018-06-14

**Authors:** Faye Lander, Thembi Russell

**Affiliations:** School of Geography, Archaeology and Environmental Studies, University of the Witwatersrand, Johannesburg, South Africa; University at Buffalo - The State University of New York, UNITED STATES

## Abstract

This paper is a response to the growing reference to archaeological evidence by linguists and geneticists interested in the spread of early farmers and pastoralists in southern Africa. It presents two databases. The first contains the archaeological evidence for pastoralism and farming in southern Africa, for the period 550 BC to AD 1050. This is the first time that the seven different types of archaeological evidence that have traditionally been used to identify both spread events are presented together at this scale. This was stimulated by our interest in investigating the antiquity of an early ‘Iron Age package’ relative to the spread of single archaeological traits. The analysis shows that the package appears approximately 700 years after sites containing pottery, cattle and sheep, without agriculture, appear in the drier parts of the sub-continent. It post-dates the appearance of earlier sites with pottery associated with farmers, metal-working and cultivation in the eastern half of the sub-continent. While poor preservation undoubtedly explains the absence of some parts of the package, the analysis suggests that other explanations should be considered. The second database is a quantitative, spatial study of archaeological publications on southern African farming and pastoralism for the period 1950 to 2016, covering the same geographical area and archaeological timeframe. This is presented as a proxy for research-intensive areas in attempt to show the gaps in archaeological fieldwork and knowledge.

## Introduction

Archaeologists have long grappled with the identification of past movements of people through the examination of their material culture. Consensus has not been reached on how, why and with whom livestock first spread in southern Africa, but recent genetic analyses, in particular, have freed archaeologists to once again talk of demic diffusion as an explanation for change, particularly for the spread of livestock without agriculture. Genetic evidence for the relatedness of present day Bantu-language speaking Africans across the sub-continent, and the persistence in Namibian Khoesan descendants of the gene that enables adults to digest milk proteins, has allowed geneticists to see migrations [1, 2, 3] and to suggest their origins and antiquity. Yet this does not remove the longstanding problem that archaeologists face: that it is difficult to tell cultural from demic diffusion. Archaeologists need to pause, rather than scrambling to see migration in their data. This recalls a similar reaction by archaeologists to linguistic analysis in past decades [4].

There is an increasing reference to archaeological evidence by researchers outside the discipline, but this evidence has not been brought together by archaeologists. Previous research has considered the spatio-temporal spread of single traits (Table 1), this is the first time that seven different archaeological traits are presented together at this scale. This paper attempts a comprehensive summary of the material remains of pastoralists and farmers in southern Africa for the period 550 BC to AD 1050. We present a detailed spatio-temporal review of the types of archaeological evidence that have been used by archaeologists who study the movement of the first southern African farmers and pastoralists. A second dataset provides an overview of those areas where the most research has been carried out in southern Africa, in order to identify the lacunae in archaeological field survey and published research. The second dataset shows how patchy the evidence is. Any conclusions must be tentative.

**Table 1:** Databases that present archaeological traits that are related to early southern African farming and pastoralism and that contain either a spatial or temporal dimension.

Material	Coverage	References
LSA fauna	South Africa	[5]
	South Africa, Lesotho, Namibia, Kenya and Tanzania	[6]
	Namibia, Lesotho, South Africa and Botswana	[7]
Farmer and LSA pottery	Southern Africa from Latitude 10°S	[8, 9]
LSA fauna and Farmer fauna	Southern Africa	[10]
Farmer sites	Eastern and Southern Africa	[11]
Farmer pottery	Zimbabwe, Botswana and South Africa	[12]
	Sub-Saharan Africa	[13, 14]
LSA pottery and fauna, farmer pottery and fauna	KwaZulu-Natal province, South Africa	[15]
LSA stone tools	Southern Africa from latitude 10°S	[16]

We were concerned about how to label people in the database and maps. Traditionally southern Africanist archaeologists have labelled sites by the subsistence type of their occupants–farmer, herder, hunter-herder, for example. Conventionally, livestock bones and pottery found within a Later Stone Age context (i.e. without iron tools) in the western half of southern Africa are understood to be the remnants of herders (who may or may not be related to autochthonous hunter-gatherers and who spoke languages related to the Khoe language group), whilst pottery (and later, iron tools, livestock and cereals) found on the eastern side of southern Africa is interpreted as having an agro-pastoralist origin, and is linked to the movement of Bantu-language speakers. We have retained these conventional labels for ease of comparison with other work, while recognizing that they may be problematic [6]. The detailed description of the archaeological finds at each site in the database allows the reader to interrogate their strength and to understand which proxies are commonly used for farming and pastoralism in the discipline. Whilst the database and maps refer to ‘herders’ and ‘farmers’, our naming of the database, outlined below, is in recognition of the difficulty of moving from archaeological evidence to subsistence type and group identity.

In summary, the objectives are:

- To present a spatio-temporal review of multiple archaeological traits that are taken as evidence for farming and pastoralism in southern Africa.
- To investigate the antiquity of an early ‘Iron Age Package’ relative to the spread of single archaeological traits.
- To look for patterns that might be missed at a smaller scale of analysis.
- To present an overview of the coverage and quality of the archaeological data.
- To investigate whether archaeological classifications have been over-influenced by the modern distribution of linguistic groups.

## Materials

Two geo-referenced databases and a southern African language distribution map are presented. The first database (Database 1) is named the ‘Southern African Database for the appearance of Livestock, Pottery, Metal and Crops’ (S1 Table). The naming reflects our reluctance to definitively identify the occupants of a site as farmers or pastoralists–although, most commonly, farmer sites are identified by archaeologists on the presence of a minimum of pottery type and geographical position, and pastoralist sites by the minimum evidence of livestock and geographical position.

There are multiple ways of being a *pastoralist* and here we use it very broadly to describe livestock-keepers without agriculture. It thus encompasses all the various terms (hunter-herders, hunters-with-stock, stone-tool-using herders) that have been used to describe livestock-keepers by southern Africanist archaeologists. We use the term *hunter-gatherer* to describe LSA sites where no livestock are found.

### The southern African database for the appearance of livestock, pottery, metal and crops (Database 1)

This is the first database to provide a chronological, geo-referenced list of the material that is commonly used by archaeologists to identify the presence of early farmers and pastoralists in southern Africa. The database contains geo-referenced and radiocarbon-dated archaeological evidence for farmers and pastoralists from 209 sites dating to between 550 BC to AD 1050 in southern Africa. Southern Africa includes all countries which lie to the south of Congo, the Democratic Republic of Congo and Tanzania. The cut-off of 1050 AD is chosen to exclude the Later Iron Age and the increasing socio-political complexity that occurs thereafter. Farmers are traditionally identified in the archaeology when the remains of domestic livestock, crops, iron tools, iron working, diagnostic pottery, daga walls and floors, grain bins, pits and livestock byre floors or enclosures are found. Together these constitute what has been called the ‘Iron Age Package’ or the ‘Chifumbaze complex’ [17]. This archaeology is conventionally understood to be that of Bantu-language speaking farmers.

Pastoralists are identified by the remains of livestock, pottery type and the geographical location of the site. Sites containing evidence of livestock and/or pottery without agriculture might contain other items of material culture such as stone and bone tools, and/or ostrich eggshell and marine shell beads but we have chosen not to include these in the database. Instead we have focused on recording those items that are common to both farmer and pastoralist sites. For example, grindstones, metals, pits and stone features, when they occur at pastoralist sites, are listed in the database. Traditionally, pastoralists are understood to be non-Bantu-language speakers and ancestral to the Khoe.

We collected all available archaeological data relating to farming and pastoralism, by southern African country. All except two sites fell below latitude 10 degrees south. The two outliers, at 8 degrees south, are Benfica in northern Angola and Kalambo Falls in northern Zambia. Benfica, on the coast of northern Angola, stands out as a potentially important site because Huffman [12, 18] has identified pottery from a shell midden at Benfica as a possible origin for the Kalundu tradition of pottery, linking it to a potential westerly route for the spread of farming. The 209 sites listed in the database have a total of 475 radiocarbon dates of which 40 are direct dates (Table 2).

**Table 2:** A summary of the number and type of dates for LSA and farmer sites in Database 1.

Type of site	Number of sites	Number of conventional dates	Number of direct dates
LSA	93	176	20 (livestock bone)
			8 (fibre from pottery)
			1 (charcoal from pottery
Farmer	113	257	5 (carbon from pottery)
			1 OSL (pottery)
			2 (charcoal from pottery)
Metal-workers	3	2	3 (charcoal from pottery)
**Total**	**209**	**435**	**40**

### The southern African farmer and pastoralist publication database (Database 2)

Database 2 presents a spatio-quantitative study of the numbers of publications on farmer and pastoralist archaeology for the period 1950 to 2016 for the research area (S2 Table).

### Southern African language map

This map presents the distribution of extant southern African languages. It excludes European languages and Afrikaans. Whilst the Bantu language distribution is based on the modern distribution of these languages, the distribution of Khoe languages is based on their precolonial distribution (after [19]).

## Method

All maps were created using ArcGIS version 10.5. The base map for Database 1 was compiled using the freely available USGS (US Geological Survey) Africa Digital Elevation Map.

### Compilation of the southern African database for the appearance of livestock, pottery, metal and crops (Database 1)

The database was compiled from a combination of site reports, academic publications, radiocarbon laboratory lists and existing databases in print. It expands on previous spatio-temporal databases that focused on single traits in southern Africa (Table 1). The principle source journals for radiocarbon data and geographic coordinates are *Radiocarbon* and the *Journal of African History*. Other journals publish site specific reports which contain the detail required to understand what a radiocarbon date is associated with. A full list of journals and reports consulted whilst compiling the database are provided in S1 Text. The database contains these fields:

1. A unique site identifier and its location by country.
2. The official site name, the type of site and the subsistence base of its occupants as designated by archaeologists.
3. Uncalibrated radiocarbon date and laboratory number. Direct dates are noted in bold alongside the material dated.
4. Calibrated date ranges. Dates were calibrated using OxCal 4.2 and the southern hemisphere calibration curve ShCal 13. Calibrations are to 1 sigma and reported as calibrated years BC/AD.
5. Average date ranges are given in cases where there is overlap in the calibrated date ranges of multiple dates at a site and/or where a researcher recognizes a single occupation phase which has multiple dates.
6. The radiocarbon dates are given the following rating:
  1. Good. A direct date.
  2. Fair. Sequence of dates with relatively good stratigraphic control/excavator is highly confident of the association between dates and materials.
  3. Poor. Single radiocarbon dates or poor stratigraphic control and/or poor correlation between radiocarbon date and associated material and/or excavator notes that the date is unreliable.
7. A number of archaeological sites were excluded from the database (S3 Table). The criteria for exclusion from Database 1 are as follows:
  - Insufficient detail was provided about an archaeological trait and its context.
  - Reports were not accessible.
  - The radiocarbon date is considered an outlier (i.e. very old or very young).

The sites are grouped temporally into eight 200 year time slices: 1: 551–351 BC; 2: 350–150 BC; 3: 149 BC-AD 51; 4: AD 52–252; 5: AD 253–453; 6: AD 454–654; 7: AD 655–855; 8: AD 856–1056. We are cognizant of the fact that the way in which data are grouped can manipulate patterns. However the information in Database 1 allows the reader to see the finer chronology for the appearance of archaeological traits within each time slice and their overlap. The use of a tight time range is to try to ensure that things that are plotted on the same map are contemporaneous, although the calibration distributions on radiocarbon dates constrain how refined these slices can be.

To show the spatio-temporal co-occurrence of archaeological traits at a site, a coding system is used. It represents the 12 combinations of types of archaeological evidence that may be present at archaeological sites across southern Africa. These are simplified for easy visualization; for a detailed description of each of the archaeological traits at a particular site see S1 Table. Code i and ii are only found in combination with one of the codes 1–10. The codes are:

Code 1: Site with pottery.
Code 2: Site with livestock.
Code 3: Site with pottery and livestock.
Code 4: Site with pottery, livestock and metal artefacts.
Code 5: Site with pottery and metal artefacts.
Code 6: Site with pottery and metal-working.
Code 7: Site with pottery, metal-working and cultivation.
Code 8: Site with pottery, metal-working, cultivation and livestock.
Code 9: Site with pottery, metal-working and livestock.
Code 10: Site with pottery and cultivation.
Code i: Site with structures (including pits).
Code ii: Site with livestock byres.

The following criteria were used to compile the codes:

- Presence of cultivation: carbonized seeds, seed impressed pottery and/or raised granary structures. Grindstones were not taken as a proxy for cereal cultivation but their presence is noted in the database.
- Presence of livestock: faunal remains and livestock byres.
- Presence of metal use: metal artefacts.
- Presence of metal-working: slag, tuyere and other fragments; bloom, ore, and furnace remains.
- Presence of structures: daga, floors, post-holes and pits.
- Presence of byre: phytolith analysis of soil and dung layers.

For farmer sites, in particular, there is frequently inadequate information on the association between radiocarbon dates and the archaeological finds. This means that it is difficult to determine exactly when a particular item was first introduced at a site. For example a site may be described as having a date range that covers 4^th^ to 8^th^ centuries AD but we have no way of knowing where in this 400 year period a particular find (e.g. a cattle tooth) fits.

Livestock are reported as Minimum Number of Individuals (MNI) represented by (M) in Database 1. When MNI is not available the number of individual specimens (NISP) is given, represented by (N) in Database 1. In cases where livestock are reported without a breakdown with stratigraphy, only the total livestock at the site can be presented.

### Compilation of the southern African farmer and pastoralist publication database (Database 2)

The following online publication databases were accessed (1) Ebsco Host (Academic Search Complete and Africa-Wide Information); (2) ProQuest; (3) Taylor and Francis and (4) Jstor. We searched by country name, the term ‘archaeology’ and one of the following additional search terms: Iron Age, Later Stone Age (LSA), farming communities (early and late), pottery, livestock, herder, hunter-gatherer and hunter-herder.

**Fig 1:**
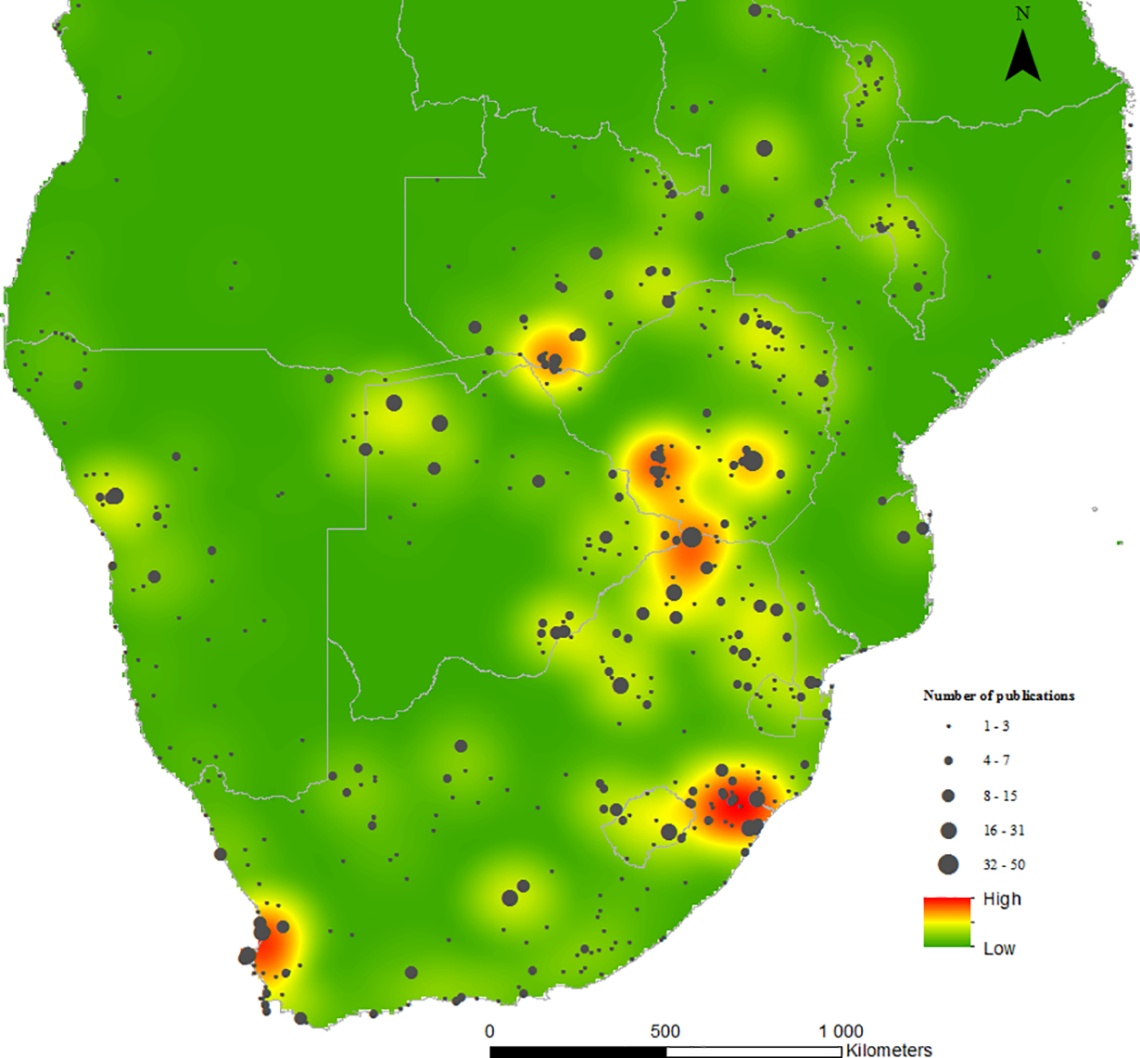
Quantitative spatial distribution of publications relating to LSA and farming research for the period 1950 to 2016.

All language categories were searched with the majority being in English; less frequently were, in order, Portuguese, German and French. Rock art research was omitted from the study as our focus was on survey and excavation for occupation sites. Once the results were reviewed, references were identified and included that were missed by the search (listed as ‘Personal documentation’ in Table 3). The initial search, which was hugely inflated, was whittled down to 1451 publications (Table 3). These 1451 publications were then examined for their geographic focus and to confirm their relevance for this study. When a single publication referred to multiple archaeological sites then this publication was counted against the tally of total number of publications for each of the sites, therefore, the total publication count rose to 2033 publications (S2 Table). In cases where archaeological research was across an area, then a central set of geographic coordinates was taken for plotting purposes (S2 Table). There are a total of 609 geo-referenced ‘publication’ points which were used to plot the map. An example of the full electronic search strategy is included in S2 Text. The map was compiled by overlaying a graduated symbol map (to show the geographic distribution and quantity of publications for each research area) on to an Optimized Hot spot map. This is a spatial statistical method in ArcGIS to identify high and low value regions, in this case a proxy for high and low research coverage based on publication count (Fig 1).

**Table 3:** Total publications for each southern African country as accessed through online academic databases (accessed between 21 July 2017 and 21 August 2017).

Country	Ebsco Host Academic Search Premier	ProQuest	Taylor & Francis	Jstor	Personal documentation	Total
South Africa	402	62	9	248	1	722
Swaziland	1	2	0	4	4	11
Lesotho	8	3	1	6	0	18
Botswana	97	13	2	49	16	177
Zimbabwe	50	12	8	84	0	154
Namibia	116	3	0	27	0	146
Mozambique	28	2	0	3	22	55
Malawi	16	9	4	17	10	56
Zambia	38	7	13	50	0	108
Angola	15	0	0	5	2	22
**Total**	**763**	**109**	**37**	**487**	**55**	**1451**

### Compilation of the southern African language map

The language map was compiled by reference to the online sources: glottolog.org, ethnologue.com and sil.org (accessed February 2018). These databases of world languages provided the modern day distribution of different language groups. Glottolog and Ethnologue provide a classification for western and eastern Bantu-language speakers. Other reference material was also consulted to validate the map–an overview of Western, West-central and Eastern Bantu languages [20], an updated version of Malcolm Guthrie’s classification of the Bantu languages [21], a classification of Western Savanna Bantu languages [22], Western and Eastern Bantu language distributions in Zambia [23], and the classification and distribution of extant pre-colonial Khoesan languages [19].

## Results

**Fig 2:**
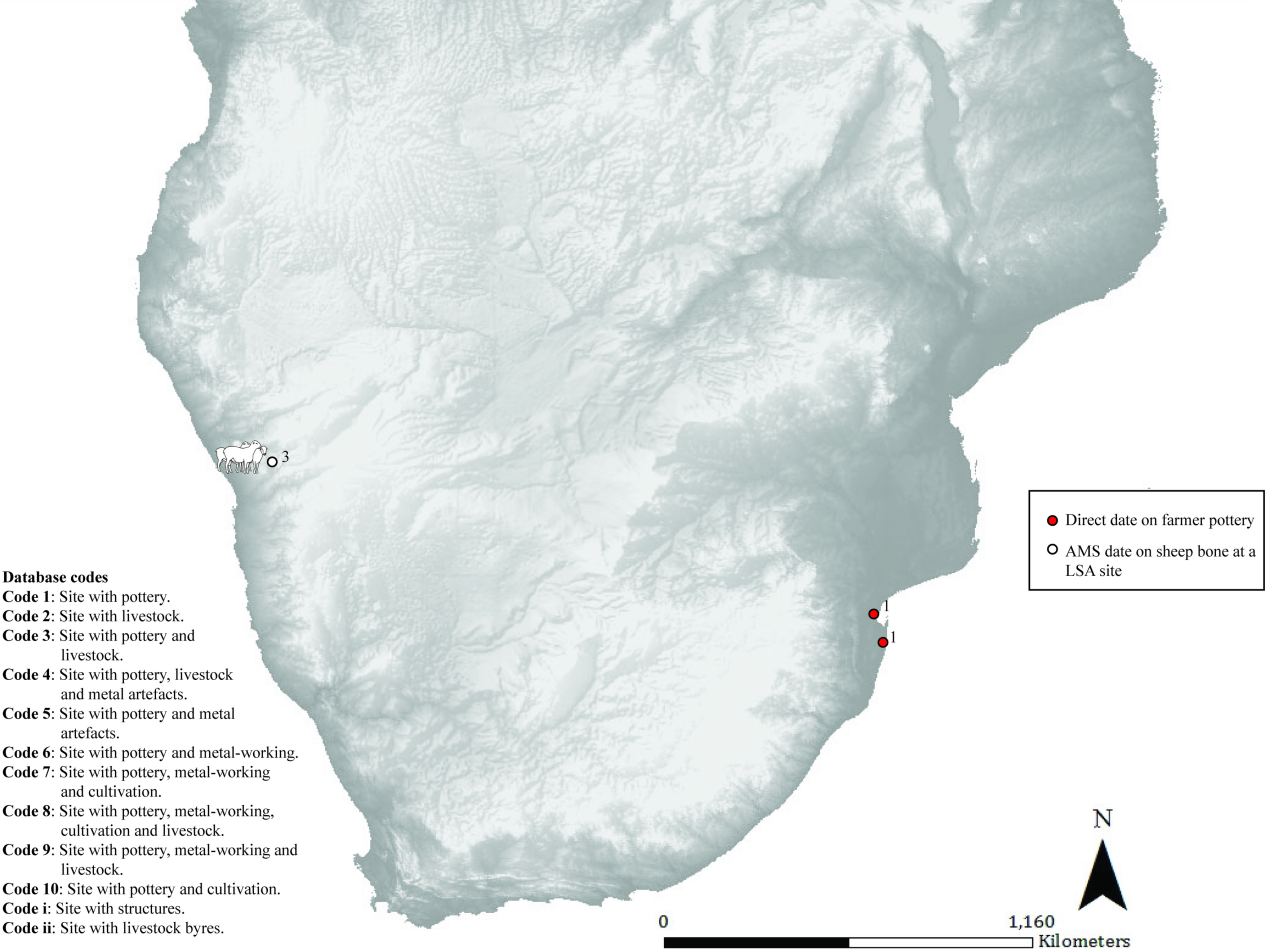
Map 1. Archaeological evidence for pastoralism and farming for the period 551–351 BC.

**Fig 3:**
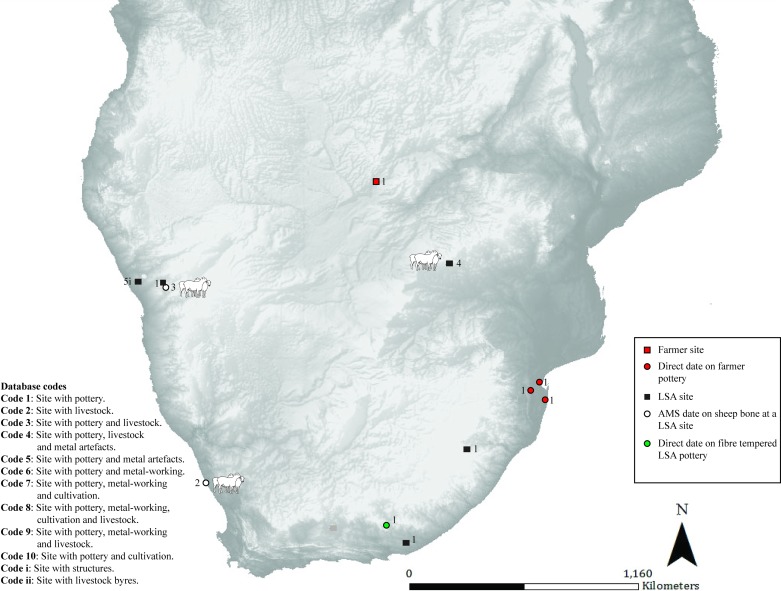
Map 2. Archaeological evidence for pastoralism and farming for the period 350–150 BC.

**Fig 4:**
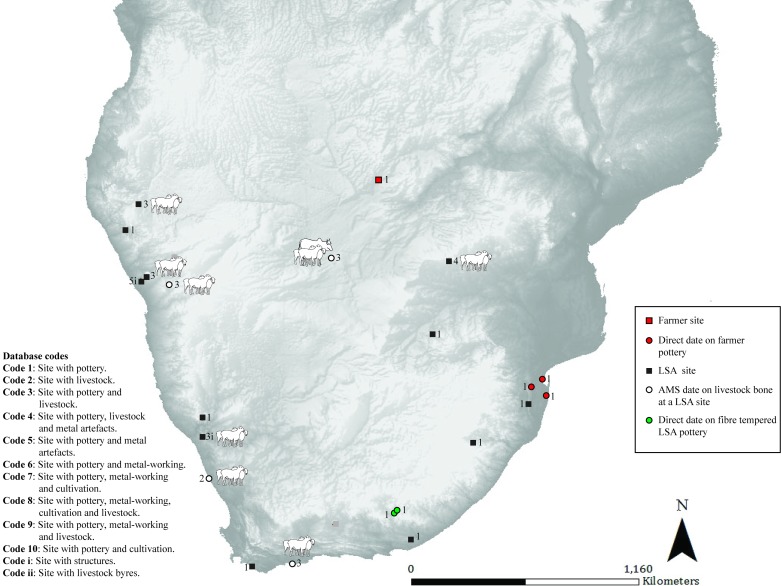
Map 3. Archaeological evidence for pastoralism and farming for the period 149 BC–AD 51.

**Fig 5:**
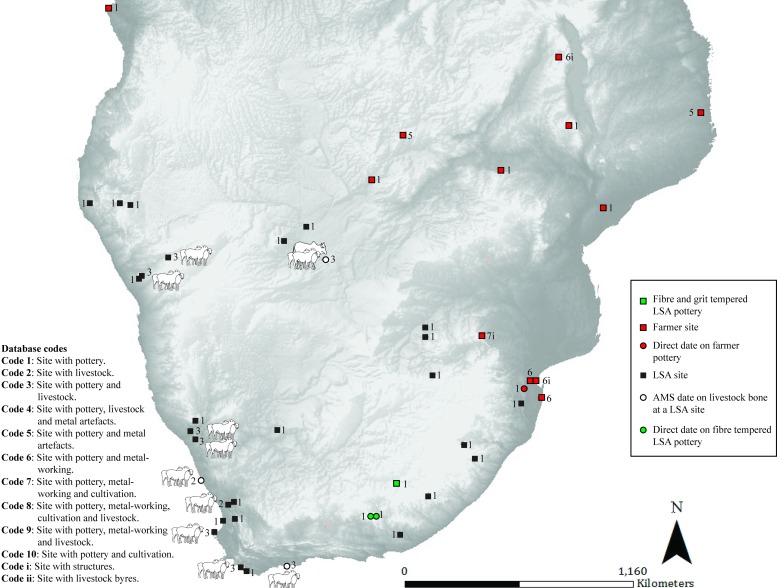
Map 4. Archaeological evidence for pastoralism and farming for the period AD 52–252.

**Fig 6:**
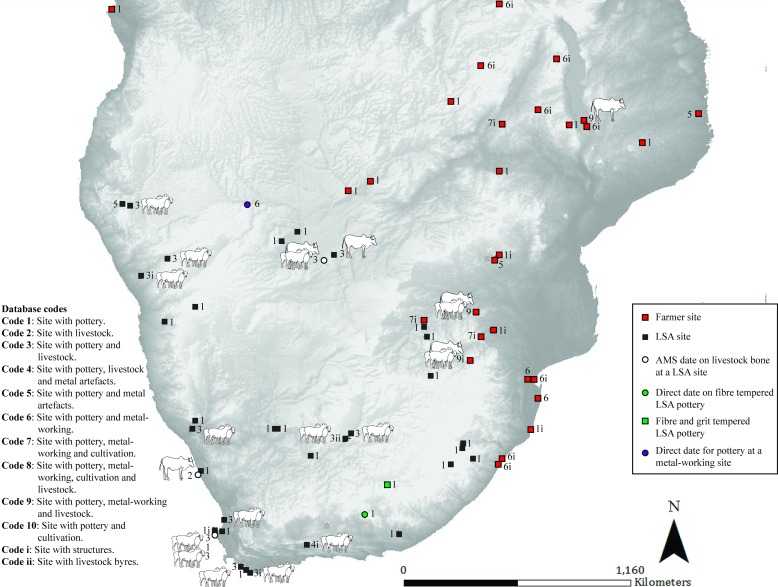
Map 5. Archaeological evidence for pastoralism and farming for the period AD 253–453.

**Fig 7:**
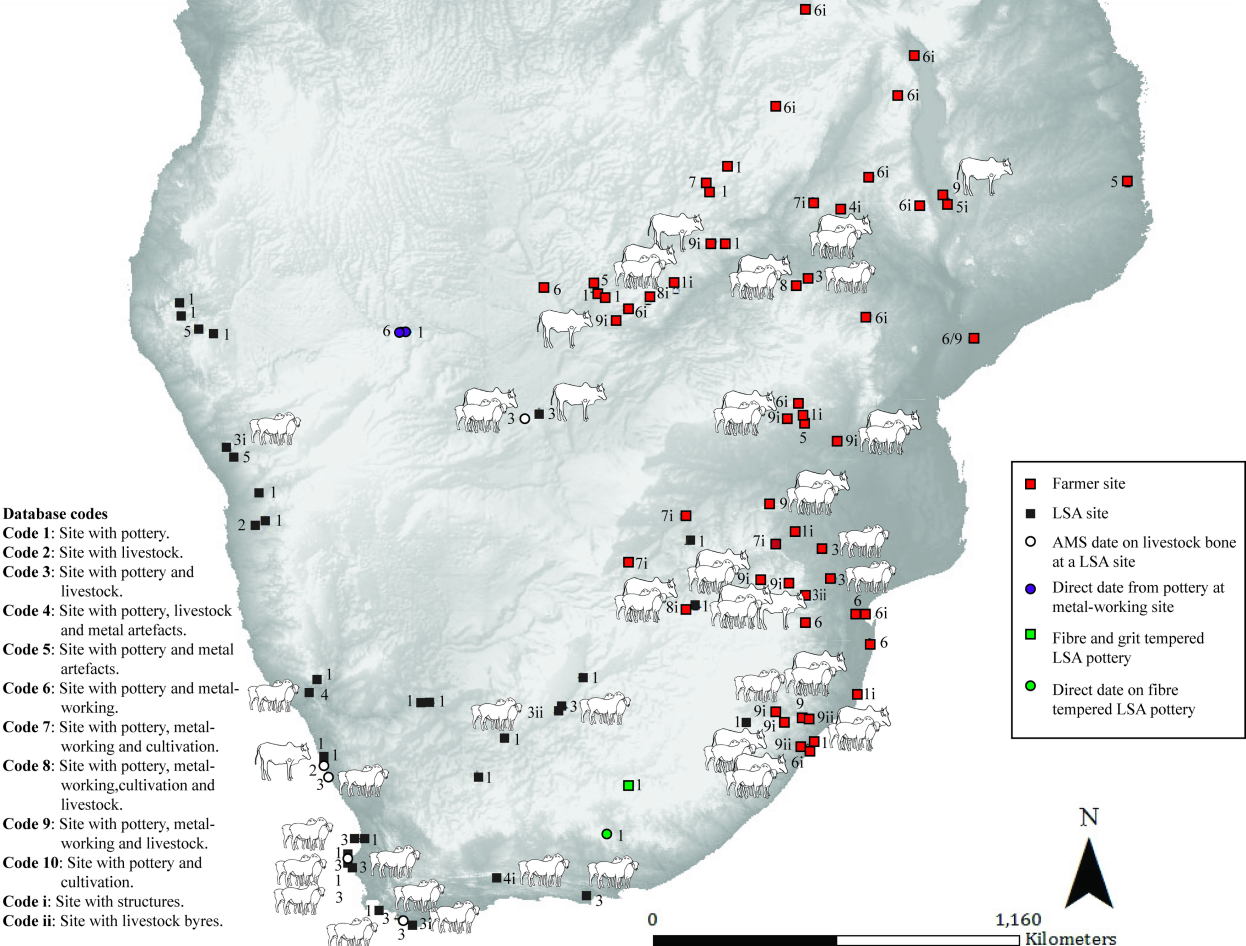
Map 6. Archaeological evidence for pastoralism and farming for the period AD 454–654.

**Fig 8:**
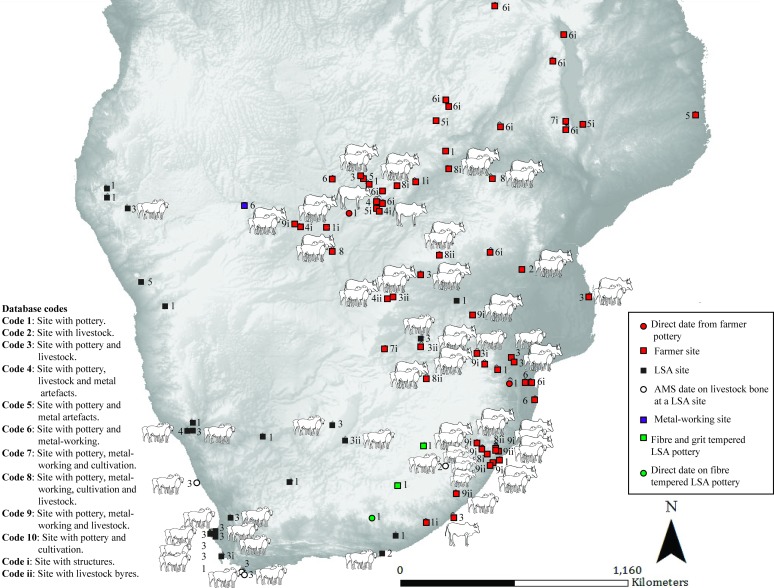
Map 7. Archaeological evidence for pastoralism and farming for the period AD 655–855.

**Fig 9:**
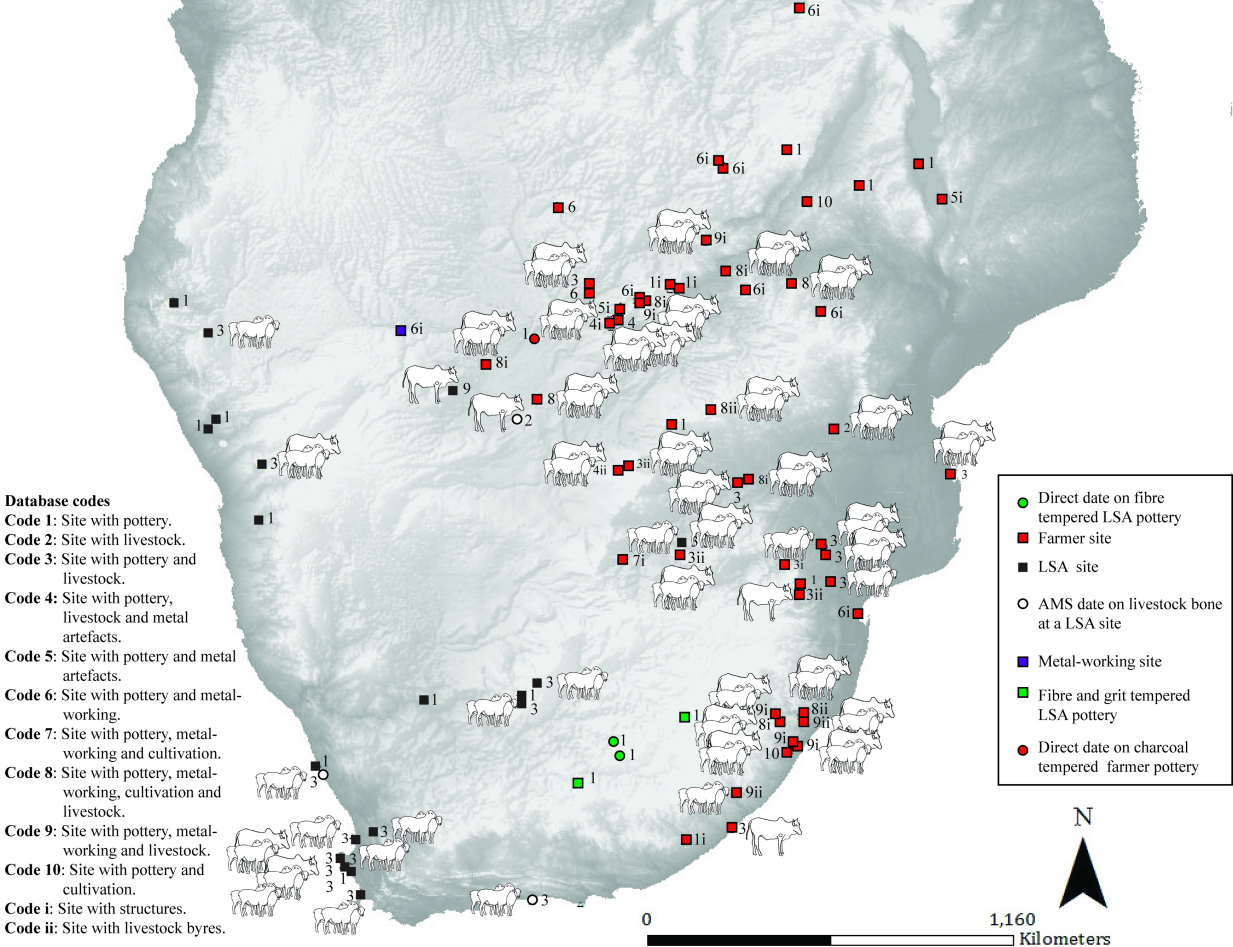
Map 8. Archaeological evidence for pastoralism and farming for the period AD 856–1056.

The maps (Figs 2–9) (S1–S8 Figs) and S1 Table are designed to be read in conjunction and they follow the same chronological order. Key events for each of the two hundred year time slices are summarized below. The conventional interpretation as to the subsistence base of groups (farmer, pastoralist, hunter-gatherer and occupants unclassified) at a site is noted in brackets after data is presented below. A summary of the chronological appearance of livestock and directly dated pottery at southern African sites is given in S3 Text.

### Map 1 (551–351 BC) (Fig 2): Earliest LSA sheep and pottery

In southern Africa the earliest evidence for sheep and undecorated pottery comes from the western half of the sub-continent. Directly dated sheep bone and associated pottery are found at Leopard’s Cave in Namibia (pastoralist) [24]. This is followed by the earliest evidence of decorated pottery from direct dates on pottery at two sites in southern Mozambique (farmer) [25].

### Map 2 (350–150 BC) (Fig 3): Pottery in multiple contexts

Further directly dated decorated pottery is found in the same area in Mozambique (farmer) [25]. Bambata Cave in Zimbabwe has evidence for caprines, decorated Bambata pottery, two spouts and a copper bead, although the dating is poor (pastoralist or hunter-gatherer) [26]. The earliest pottery from a South African context is found at Boundary Cave in the southern Cape with a direct date obtained from fibre temper in the pottery (hunter-gatherer) [9]. Soon after thin-walled, grit-tempered pottery is found in LSA contexts in KwaZulu-Natal and the Eastern Cape (hunter-gatherer or pastoralist) [15, 27]. The earliest sheep from a South African context are found at the LSA site of Spoegrivier Cave and are directly dated to this period (pastoralist) [28]. In Zambia, there is the first evidence of a farmer presence on the basis of decorated pottery, however, its association with radiocarbon dating is poor [29]. The last site occupied in this time period is Messum in Namibia which has evidence of pottery, pits and iron loops–the association between artifacts and dates are poor (occupants unclassified) [30, 31].

### Map 3 (149 BC- AD 51) (Fig 4): Farmer sites within a restricted area

This period sees the appearance of more LSA sites with pottery and sheep in Namibia (pastoralist or hunter-gatherer). The earliest evidence of cattle in southern Africa is directly dated to an LSA site in Botswana, where they are found in association with sheep (pastoralist or hunter-gatherer) [32]. Decorated pottery with lugs, caprines and pits are found together at /Ai Tomas in South Africa (pastoralist) [28]. Pottery is found at further sites within a variety of LSA contexts that cannot be confidently linked to any particular subsistence strategy/group. Sites with sheep (directly dated) and pottery on the southern Cape coast appear for the first time (pastoralist). Farmer sites, identified by their decorated pottery proxy, are found within a restricted area.

### Map 4 (AD 52–252) (Fig 5) First evidence of crops in a farmer context

Sheep and pottery are found in further LSA context sites, along the western and southern coast of southern Africa (pastoralist or hunter-gatherer). Pottery is found in a variety of contexts. For the first time evidence of metal-working, metals and daga remains are found alongside pottery at farmer sites. This period has the first evidence of crops at a farmer site dating to mid-3^rd^ century AD—impressions of bulrush millet in pottery at the site of Silver Leaves, South Africa [33]. Farmer sites appear north of the Zambezi. Direct evidence of livestock is yet to be found at a site associated with farming in southern Africa.

### Map 5 (AD 253–453) (Fig 6): Earliest evidence of cattle in farmer context

There is the first evidence of cattle, a single tooth, in a farmer context north of the Zambezi (Malawi). It dates to the end of 4^th^ century AD, however, the dating is poor [34]. During this period LSA sites with livestock (15 sites) outnumber farmer sites with livestock (3 sites). Decorated pottery and iron-working are found at sites along the southern African East coast from southern Mozambique to KwaZulu-Natal, South Africa, with no evidence for cattle or crops at these coastal sites (farmer). Caprines and cattle from two sites in South Africa date to the 5^th^ century AD, this is their earliest occurrence at farmer sites in South Africa. Further sites appear with metal-working, metal artefacts and pottery in Namibia (occupants unclassified) [35]. Sheep and some cattle are found across the drier parts of southern Africa, sometimes associated with pottery (pastoralist or hunter-gatherer). There is the first appearance of sheep in an LSA context in the interior of South Africa (end of 3^rd^ century AD), although the dating is poor [36]. Cattle, identified through ancient DNA analysis of a horn core, and directly dated, first appear in an LSA context in South Africa (pastoralist), dating to the end of the 5^th^ century AD [37].

### Map 6 (AD 454–654) (Fig 7): LSA livestock at a peak. First appearance of the Iron Age package at three sites

As well as cattle and caprines appearing at many farmer sites (21 sites with livestock, 17 of which have cattle) for the first time, proxy evidence for cattle byres also appears for the first time. Cattle byres are only found at sites from the Limpopo basin southwards. Caprines and pottery predominate at LSA sites in the drier western half of the sub-continent, although cattle do occur (17 sites with livestock, 3 of which have cattle). In Zimbabwe, a possibly circular hut floor is recorded, if this is correct it is the earliest example of this type of feature [38]. This is the first time that rectangular hut floors are recognized in southern Zambia [39]. Whilst only three sites have the complete Iron Age package (code 8), there are 19 sites containing an almost complete package (code 7 or 9), making this time slice the first in which the Iron Age package is common (total 22 sites).

### Map 7 (AD 655–855) (Fig 8): Farming reaches most southerly known point

The Iron Age package is found at many sites in the eastern half of southern Africa during this period. Cattle numbers increase, with many farmer sites having evidence for pottery, metal-working, cultivation and livestock. Caprines and pottery have a continued presence in the drier western half of southern Africa. The spread of farming reaches its most southerly point near the Fish River, South Africa. This is the first time that farmer sites with livestock significantly outnumber LSA sites with livestock (18 LSA to 32 farmer). Farmer sites containing cattle are at a peak.

### Map 8 (AD 856–1056) (Fig 9)

The ratio of farmer sites with livestock to LSA sites with livestock remains almost constant from the proceeding period (16 LSA to 32 farmer). This period sees the continued dominance of cattle, caprines and the Iron Age Package at farmer sites throughout eastern southern Africa and the continued presence of pottery and caprines in LSA contexts in the western half of southern Africa.

## Discussion

This paper is a response to evidence of increasing interest from other disciplines in the spread of farming and pastoralism in southern Africa, as evidenced in cross-disciplinary papers [40, 41, 42]. We first comment on the two spread events themselves, before raising some points that speak to potential gaps and weaknesses in archaeological research and method.

### The spread events

The main contribution of our analysis is to suggest five patterns in the archaeology of this period. New archaeological finds may well change such patterns. Our data highlights the gaps in our evidence.

1. Contrary to prevailing assumptions, our data shows a strong and enduring pastoralist presence in the western half of the subcontinent. Most papers on the spread of pastoralism to South Africa recount the sightings of large herds of cattle by early European mariners visiting the southern shores of Africa and then discuss the absence for evidence for them in the archaeological record. Mapping at this scale reveals that which has been overlooked by archaeologists–the enduring pastoralist presence in the western half of southern Africa, ahead of and during the period of farmer expansion. This pattern is revealed because we chose to group all the various categories of LSA people who are found with domestic animals as pastoralists, rather than following previous research that focused on categorizing people with livestock to distinguish hunter-gatherer-herders from immigrant herders (for a discussion see [6]).
2. Different types of early pottery, which have been attributed to pastoralists, farmers and hunter-gatherers, appear at the same time at widely dispersed points across southern Africa (cf. [8]).
3. Crops predated livestock in farmer contexts. Livestock are absent at the earliest farmer sites on the eastern side of southern Africa. This absence of evidence has most commonly been attributed to poor preservation and the ritual disposal of bone [43], but with the greater body of archaeological data this argument is becoming less tenable—livestock remains of greater antiquity are preserved at many southern African pastoralist sites and from AD 600 onwards at many farmer sites. The current evidence favours a scenario that sees the arrival of farmers without livestock ahead of agro-pastoralists (see [44]).
4. There is an explosion of livestock, particularly cattle, at farmer sites, from the mid-7^th^ century AD onwards, contemporaneous with the appearance of the Iron Age package at many sites across eastern southern Africa (cf. [45, 46])
5. Pastoralism arrived in southern Africa ahead of farming: livestock-keeping spread rapidly from present day Namibia across a wide area southwards and eastwards (cf. [37]).

### The robustness of the archaeological evidence: Potential biases

Arthur [47] draws attention to the inherent bias in South African pastoralist research towards long occupation rock shelters. These are most commonly discovered and excavated at the neglect of open air sites. Furthermore open air sites are more likely to represent a single occupation period and thus have a more fleeting presence on the landscape. In the database the bias is clear, 67 of the 93 LSA sites are rock shelters or coastal boulder sites (of which there are only five), while 10 are coastal shell middens and 15 are inland open air sites. This bias in survey and excavation affects the mapping exercise by presenting an artificially static, and coastal, distribution, of what we assume would have been more widely distributed and mobile pastoralist groups. It is likely that as more open air sites are found and dated, the picture will become more fluid and filled.

We have made the assumption in presenting this work that the identification of cattle and caprines is valid. Recent debates about the strength of genetic identification versus morphological identification suggest we need to be cautious and that there is need for increased analysis of the ancient DNA of animal bones [48–53]. Similarly, the finds needs to be directly dated. Whilst there is an awareness of the importance of direct dating among those working on pastoralism this is not the case in early farmer research. There is not a single direct date on livestock from a farmer context in southern Africa. Research at pastoralist sites has demonstrated that livestock bones may be younger than their deposit, which has led to a concerted effort to directly date bones at pastoralist sites [54, 55]. The need for direct dating of livestock on farming sites is pressing, particularly given the dominance of an interpretative model, the Central Cattle Pattern, which relies on the early presence of cattle at farmer sites ([43, 56], see also [57]).

The dating of pottery is also problematic. It is still overwhelmingly dated by association with stratigraphic layer at LSA sites, even at those sites where bones within the same stratigraphic layer have shown to be younger (i.e. [54]).

**Fig 10:**
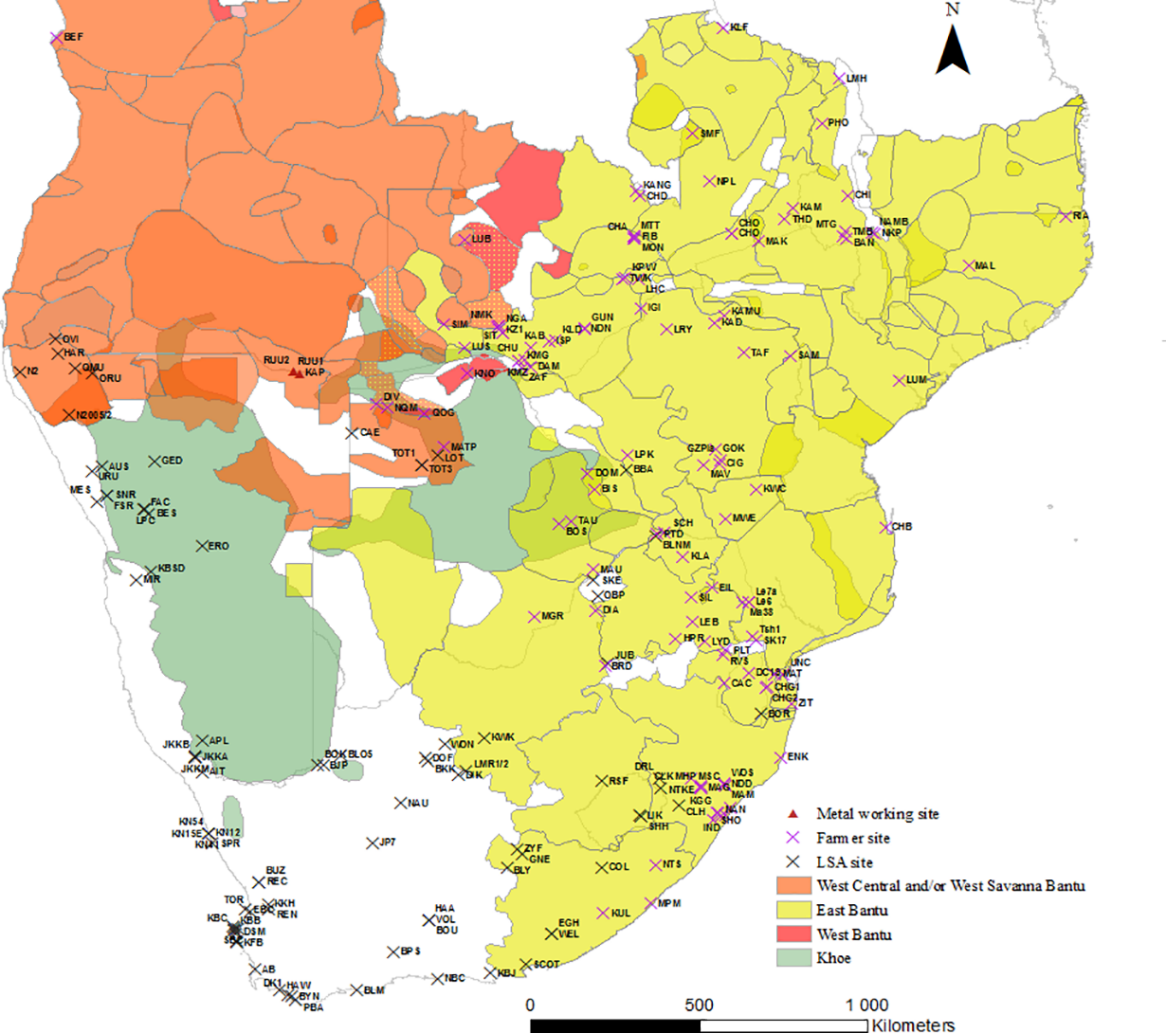
The distribution of archaeological sites in Database 1 overlaid onto the distribution map of African languages–note the almost perfect match between modern language distribution and archaeological classification.

We compiled the Southern African Language map because of a concern that there was inherent bias on the part of researchers to interpret the subsistence base of a site’s occupants based on a site’s geographical position. To explore this we have plotted all of the archaeological sites onto the language map (Fig 10). We wanted to see if there were any outliers–namely an archaeological classification that did not follow modern day language patterning. There is only one. The site of Gundu (GUN) is not classified as Eastern Bantu but falls within the modern day distribution of Eastern Bantu languages. Similarly, with the exception of a handful of sites, pastoralist LSA sites are found outside of the eastern Bantu language area. Further investigation is needed—were pastoralists absent from areas where eastern Bantu-languages dominate today? This seems unlikely. These areas would have been attractive to pastoralists given their watering and grazing potential.

**Fig 11:**
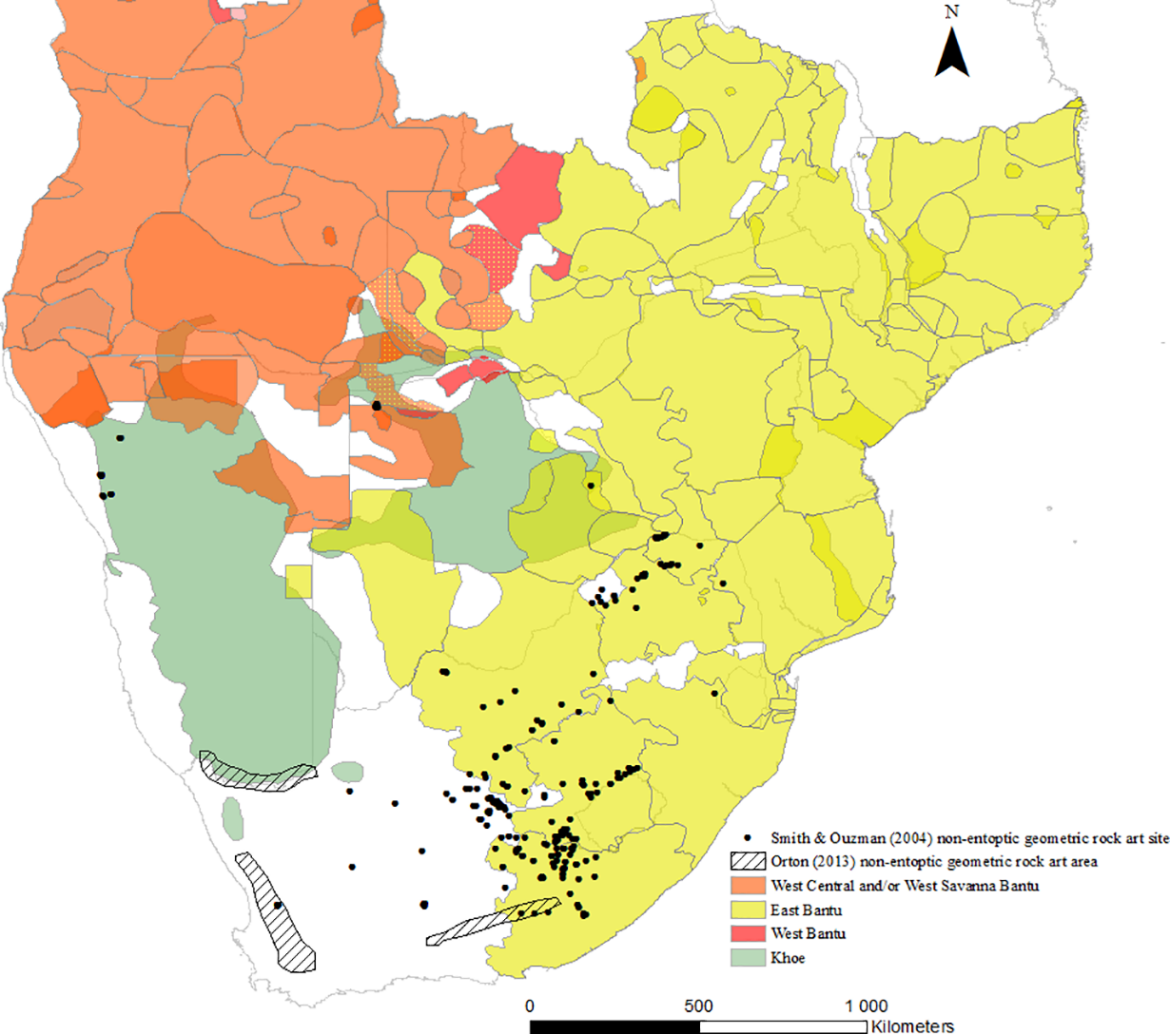
Map to show the distribution of geometric rock art (pastoralist) [58, 59, 60] compared to the distribution of African languages as an illustration of areas which might potentially contain archaeological evidence that has not yet been captured.

In 2004, Smith and Ouzman raised the hypothesis that a body of southern African geometric rock art had a pastoralist authorship [58, 59]. In Fig 11, its distribution is shown overlaid on to the language map. As the figure shows this type of pastoralist archaeology falls within the modern day distribution of eastern Bantu-language speakers and is illustrative of what might be missing from our archaeological knowledge.

When we mapped the number of publications on pastoralist and farmer research for the period 1950 to 2016, the bias in our data towards research-rich areas is clear. More research is needed in Angola, Mozambique, large parts of Botswana and South Africa’s interior. Angola is a potentially important route for the spread of pastoralists, farmers and livestock [12, 17] and yet there is hardly any archaeology from this region. The picture and patterns presented in this short paper are sure to change as these gaps are filled.

## Conclusion

This paper is a response to a growing interest in the spread of farming and pastoralism in southern Africa by other disciplines, particularly geneticists, by providing a detailed spatio-temporal overview of the archaeological evidence that is used to interpret the past spread of these groups. Sadr [8] has stressed that more might be learnt about past population movements and interactions in southern Africa by dissolving the conceptual boundary between the southern African Iron Age and Stone Age periods because they overlap in time. The patterns presented in the paper prove the strength of this argument at this scale of analysis.

Our key findings are (1) evidence for an enduring pastoralist presence in southern Africa ahead of, and during, the appearance of agro-pastoralists; (2) the early, contemporaneous and widespread appearance of pottery that has conventionally been assigned to different subsistence groups (hunter-gatherer, farmer and pastoralist), and (3), the appearance of crop farming ahead of agro-pastoralism at farmer sites. These findings are tentative. Patterns may change as more data becomes available. In particular we draw attention to the bias towards rock shelter excavation in Later Stone Age Archaeology and to the complete absence of direct dates on livestock within farming research.

## Supporting information

S1 Fig Archaeological evidence for pastoralism and farming for the period 551–351 BC. (TIF)

S2 Fig Archaeological evidence for pastoralism and farming for the period 350–150 BC. (TIF)

S3 Fig Archaeological evidence for pastoralism and farming for the period 149 BC–AD 51. (TIF)

S4 Fig Archaeological evidence for pastoralism and farming for the period AD 52–252. (TIF)

S5 Fig Archaeological evidence for pastoralism and farming for the period AD 253–453. (TIF)

S6 Fig Archaeological evidence for pastoralism and farming for the period AD 454–654. (TIF)

S7 Fig Archaeological evidence for pastoralism and farming for the period AD 655–855. (TIF)

S8 Fig Archaeological evidence for pastoralism and farming for the period AD 856–1056. (TIF)

S1 Table The archaeological evidence for the appearance of livestock, pottery, metal and crops in southern Africa (Database 1). (DOCX)

S2 Table Southern African publication count for research on farming and pastoralism for the period 1950 to 2016 (Database 2). (XLSX)

S3 Table List of archaeological sites that were excluded from Database 1. (DOCX)

S1 Text The full list of journals and reports consulted to compile Database 1. (DOCX)

S2 Text An example of the full electronic search strategy used to capture data for Database 2. (DOCX)

S3 Text A chronological summary for the appearance of livestock and directly dated pottery at southern African sites for the period 550 BC to AD 1056. (DOCX)
