# Supplementary figures and images for: The archaeological evidence for the appearance of pastoralism and farming in southern Africa
Source: PLoS One. 2018 Jun 14;13(6):e0198941. doi: 10.1371/journal.pone.0198941 (PMC6002040; doi:10.1371/journal.pone.0198941)

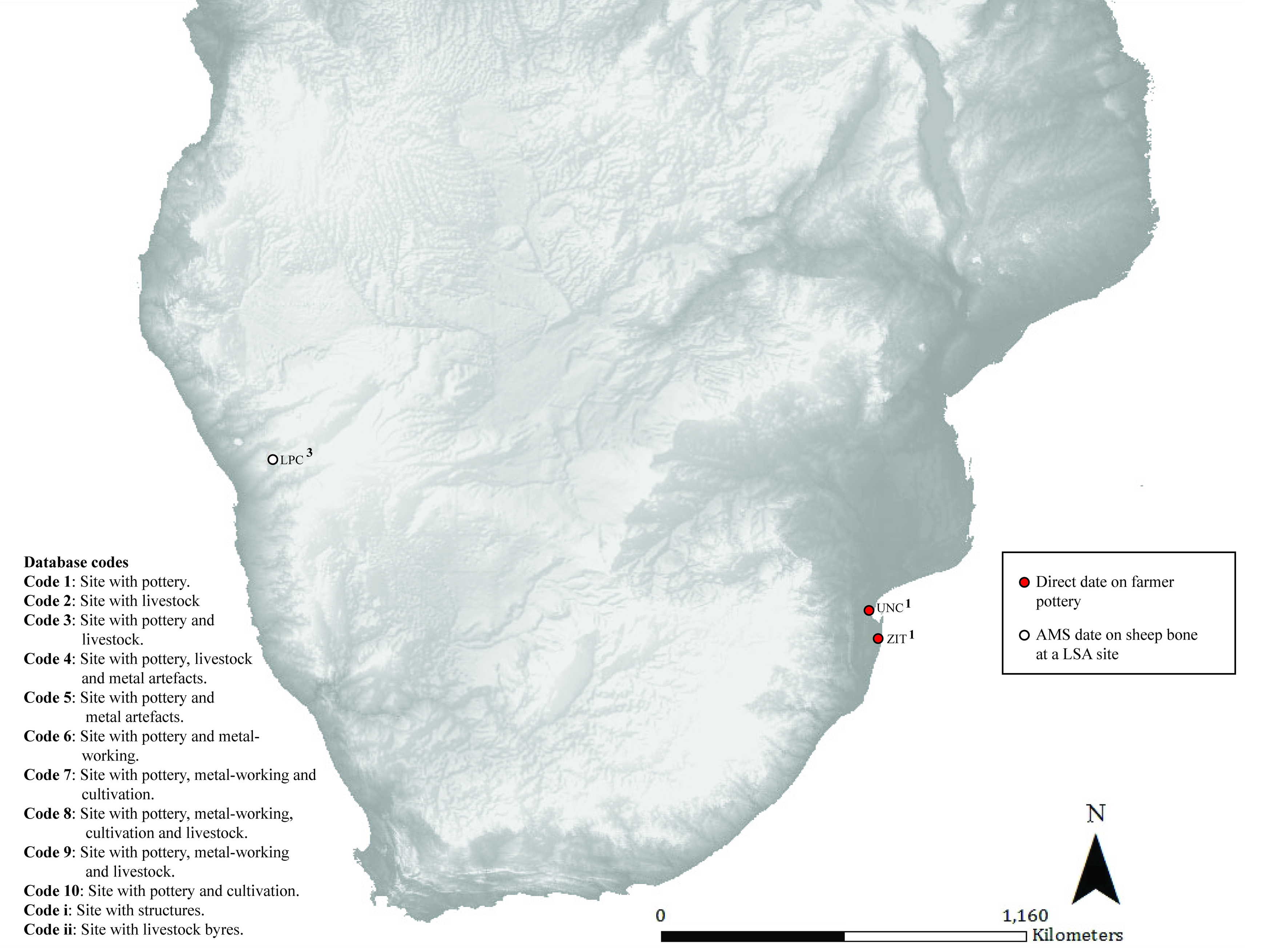

Supplement: S1 Fig — (TIF) [file pone.0198941.s001.tif]

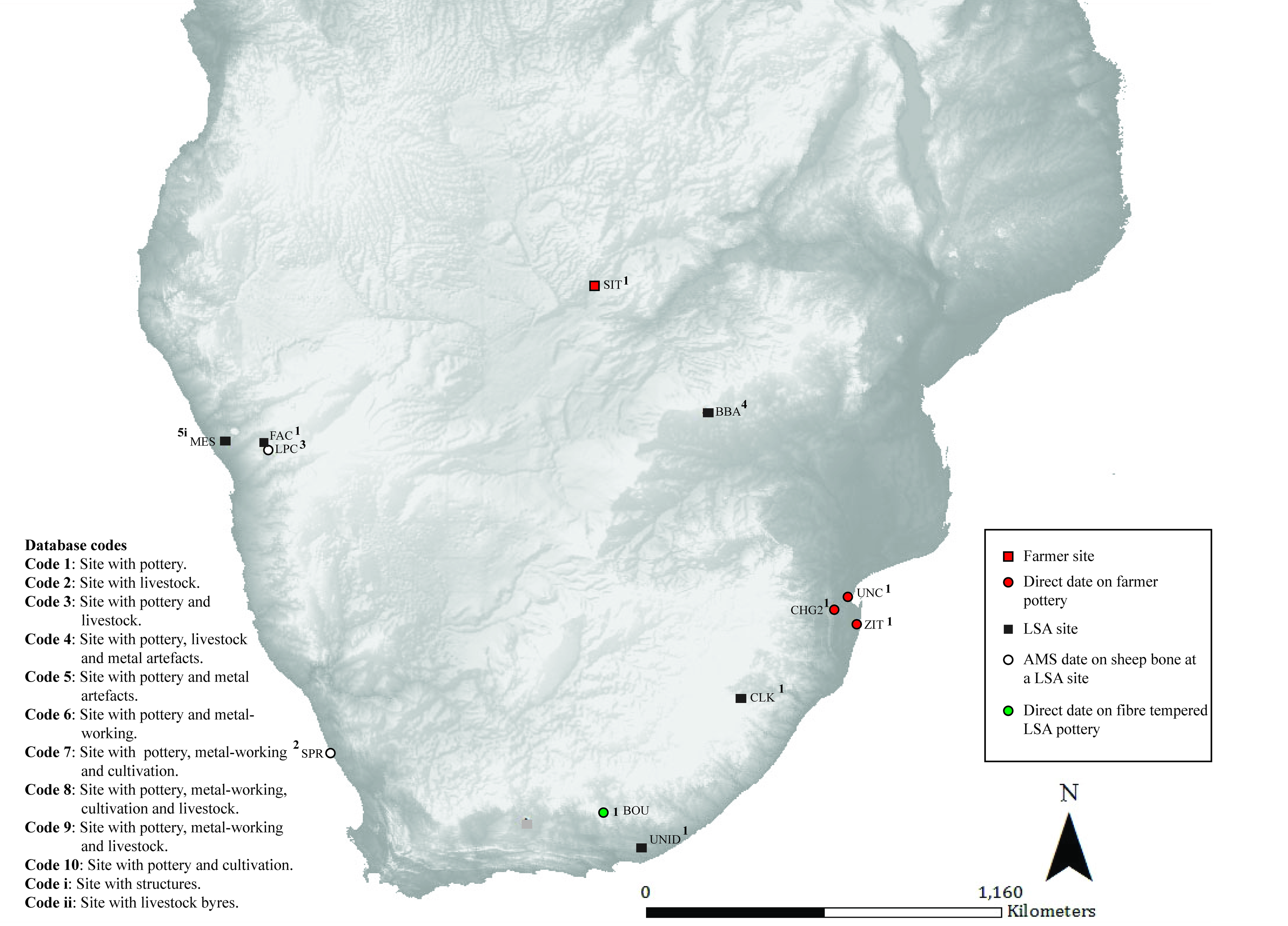

Supplement: S2 Fig — (TIF) [file pone.0198941.s002.tif]

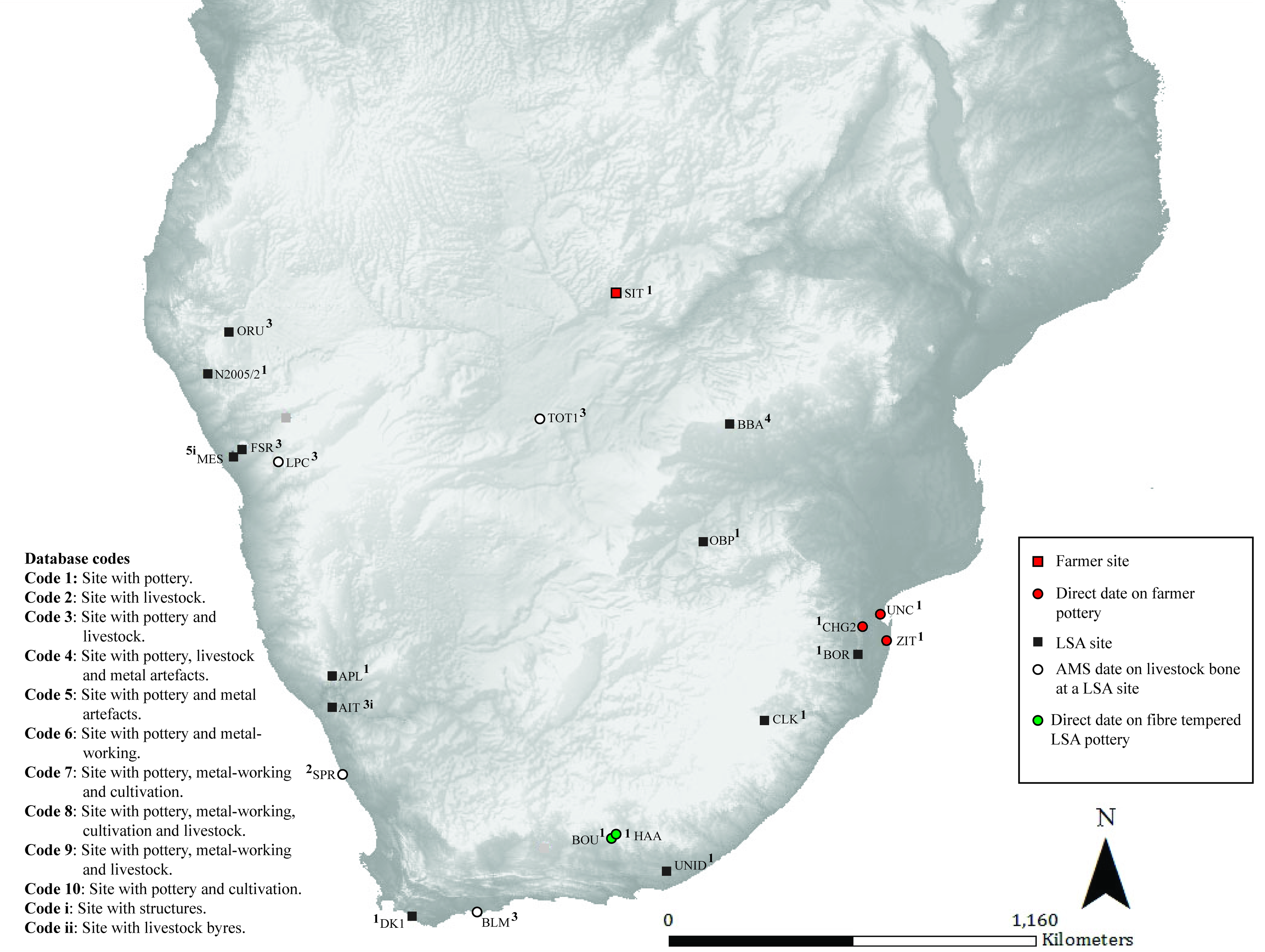

Supplement: S3 Fig — (TIF) [file pone.0198941.s003.tif]

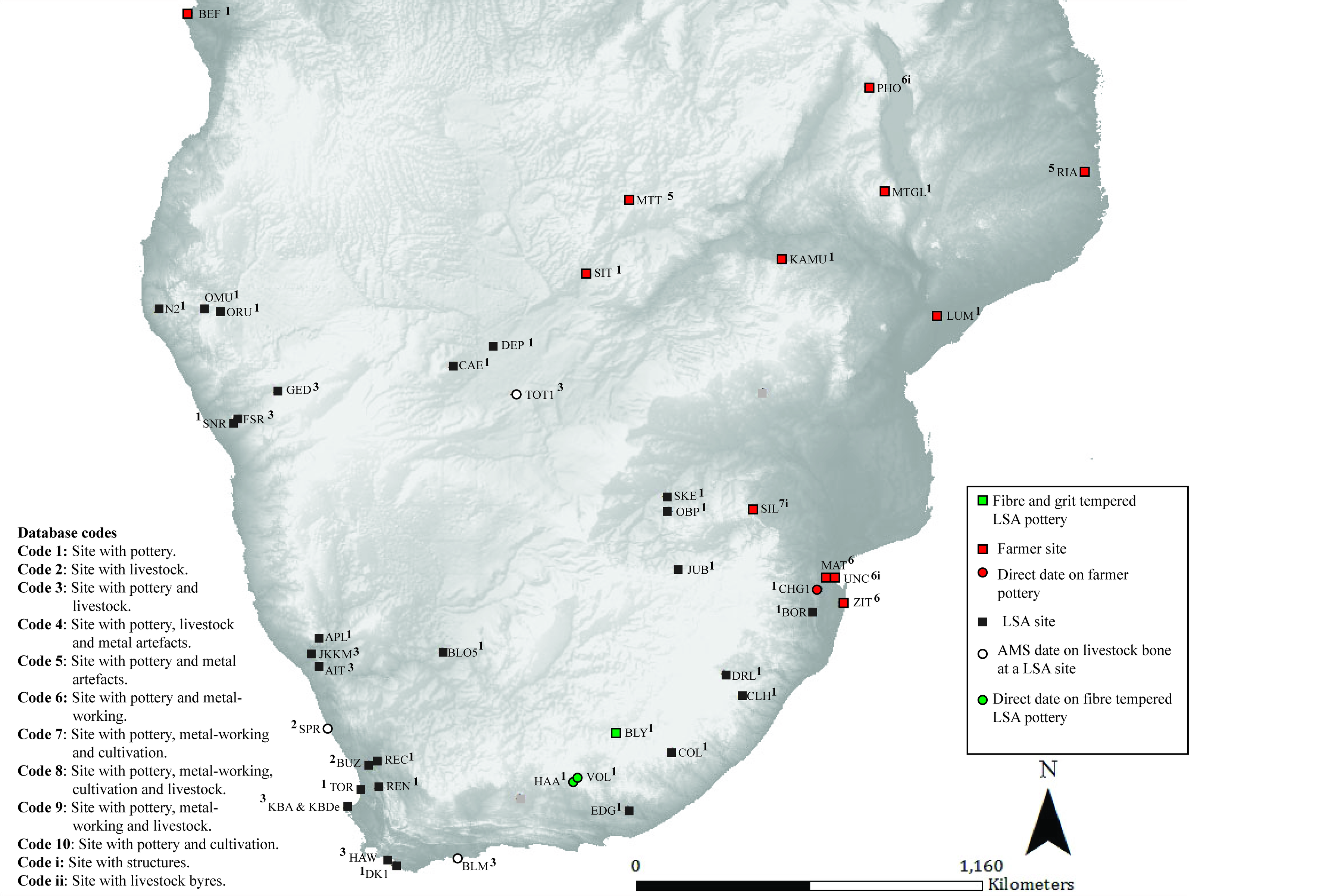

Supplement: S4 Fig — (TIF) [file pone.0198941.s004.tif]

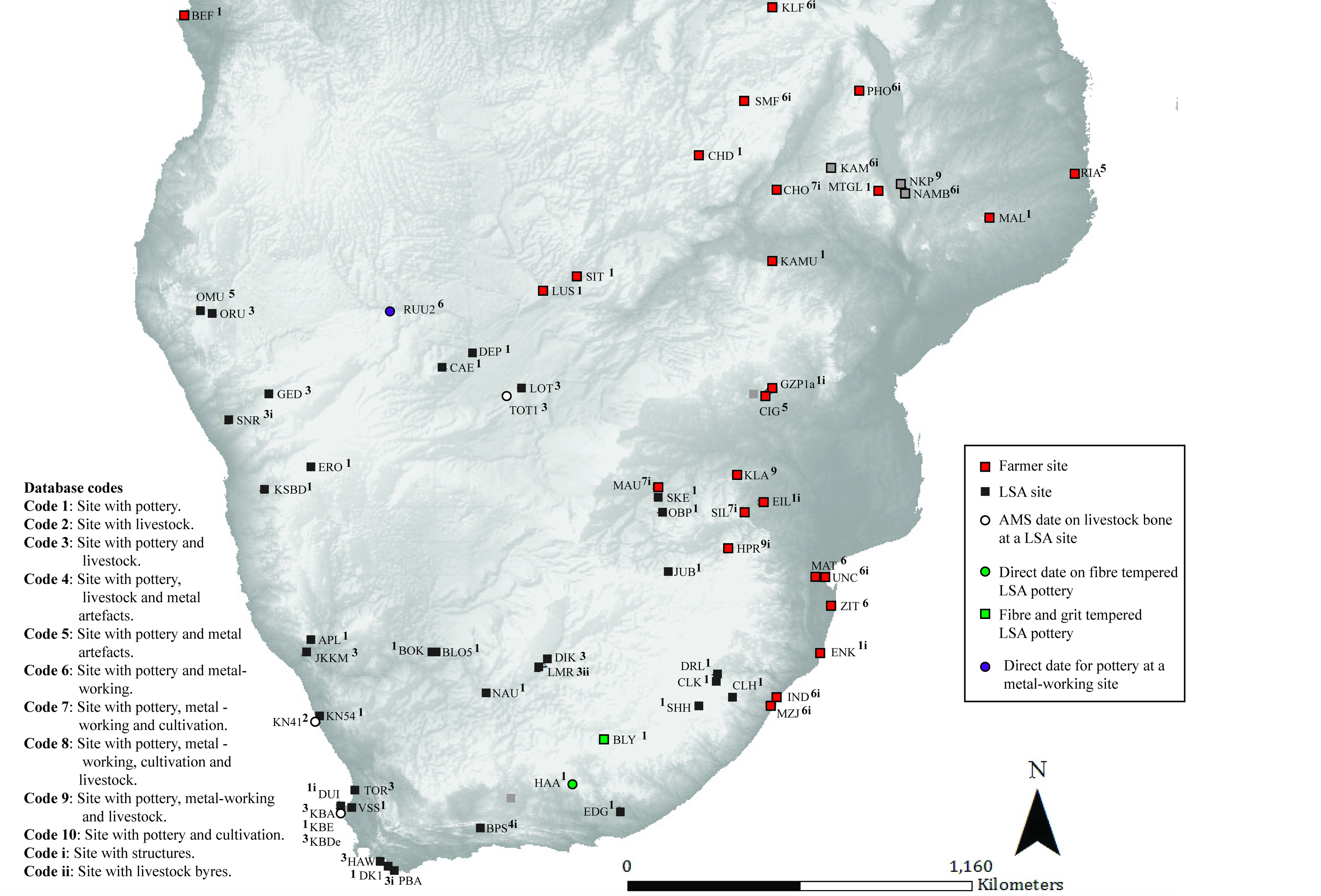

Supplement: S5 Fig — (TIF) [file pone.0198941.s005.tif]

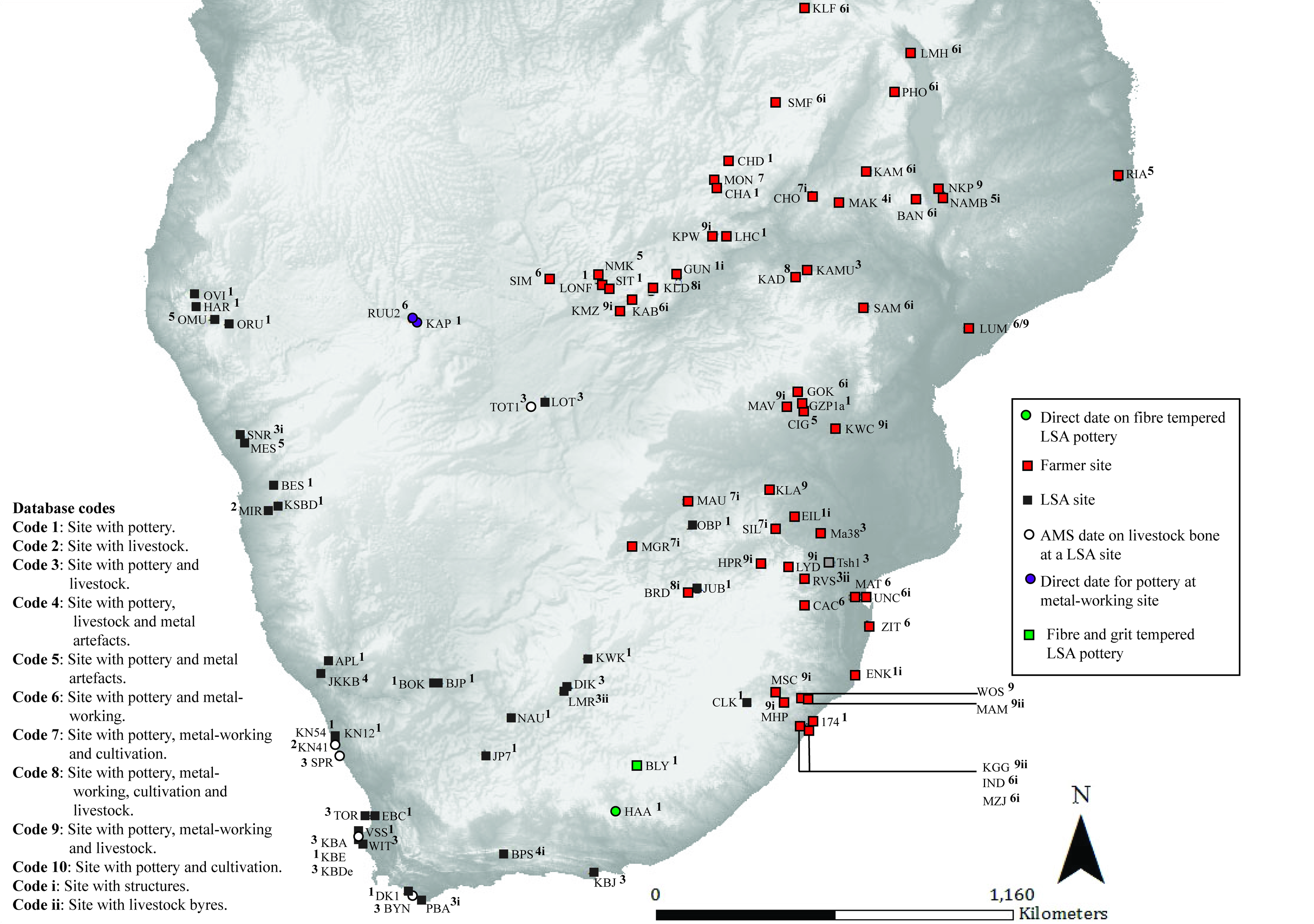

Supplement: S6 Fig — (TIF) [file pone.0198941.s006.tif]

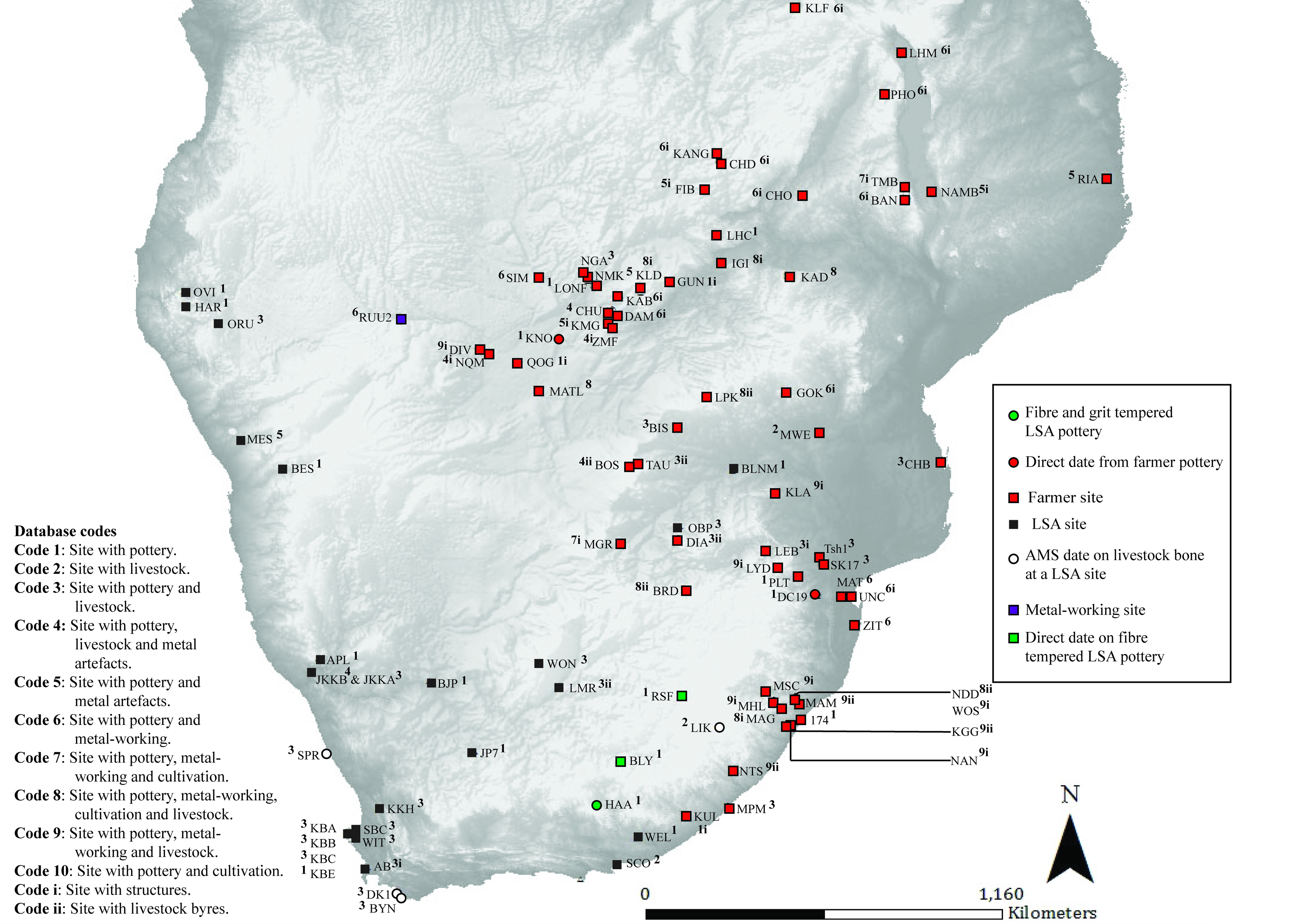

Supplement: S7 Fig — (TIF) [file pone.0198941.s007.tif]

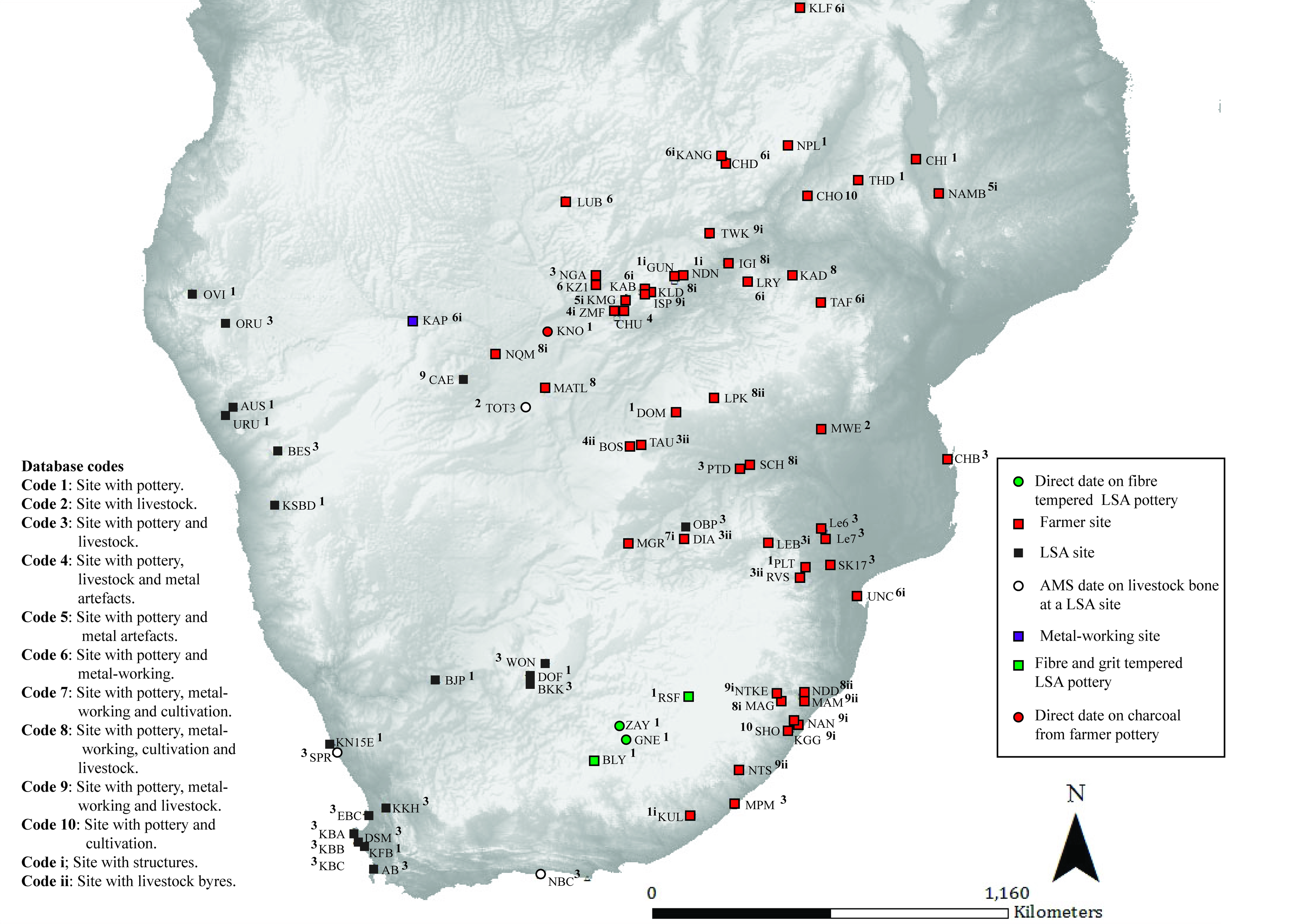

Supplement: S8 Fig — (TIF) [file pone.0198941.s008.tif]
