# Supplementary material for: The archaeological evidence for the appearance of pastoralism and farming in southern Africa
Source: PLoS One. 2018 Jun 14;13(6):e0198941. doi: 10.1371/journal.pone.0198941 (PMC6002040; doi:10.1371/journal.pone.0198941)
Supplement: S1 Table — (DOCX) [file pone.0198941.s009.docx]

| **Site ID and country** | **Site Name**  **Type of site**  **References** | **Radiocarbon date**  **Lab number** | **Calibration at 68.2% probability**  **(BC or AD)** | **Median date** | **Grade of date**  **1 – 3** | **Temporal group**  **1 – 8** | **Database code**  **1 – 10**  **i-ii** | **List of archaeological traits** |
| --- | --- | --- | --- | --- | --- | --- | --- | --- |
| **LPC**  Namibia | **Leopard Cave**  Inland LSA rock shelter/herding  [1] | 2270±40  Beta-270164  **sheep AMS**  2190±40  Beta-270163  **sheep AMS** | 370 – 211 BC  350 – 113 BC | - | 1 | 1 – 3 | 3 | - Undecorated pottery - Sheep |
| **ZIT**  Mozambique | **Zitundo**  Coastal hilltop site/farmer  [2] | 2200±50  **carbon dated pottery** | 354 – 136 BC | - | 1 | 1 – 3 | 1 | - Matola 1 type pottery: fluted rims, single bands of horizontal line incisions with diagonal or cross-hatched incisions on shoulder and body, thickened rims 7-8mm, discontinuous motif on body. |
| **UNC**  Mozambique | **University Campus**  Open-air site/Farmer  [2] | 2190±60  **carbon dated pottery** | 352 – 107 BC | - | 1 | 1 – 3 | 1 | - Matola 1 type pottery: fluted rims, single bands of horizontal line incisions with diagonal or cross-hatched incisions on shoulder and body, thickened rims 7-8mm, discontinuous motif on body. |
| **CHG2**  Mozambique | **Changalane II**  Inland open-air site/farmer  [2] | 2170±50  **carbon dated pottery** | 344 – 68 BC | - | 1 | 2 – 3 | 1 | - Matola 1 type pottery: fluted rims, single bands of horizontal line incisions with diagonal or cross-hatched incisions on shoulder and body, thickened rims 7-8mm, discontinuous motif on body. - Stone tools were found associated pottery at the site. |
| **FAC**  Namibia | **Fackeltrager**  LSA rock shelter  [3] | 2150±60  KN-I731  2190±40  KN-I732 | 337 – 328 BC  350 – 113 BC | - | 1 | 2 | 1 | - No description of pottery |
| **BBA**  Zimbabwe | **Bambata Cave**  Inland LSA cave site/pastoralist/hunter-gatherer  [4, 5, 6] | 2140±60  Pta-3072  (layer 3b) | 334 – 51 BC | - | 3  The items occur above the date and are therefore not directly associated with the radiocarbon determination. | 2 - 3 | 4 | - Bambata A pottery: 4-6mm thick; mostly jars; necked vessels and bowls, black to grey in colour, multiple bands of alternating designs, comb-stamping more common than incision, cross-hatching and punctates. Spouts were recovered at the site. - Cu bead - Sheep/goat (identification has not been confirmed). |
| **BOU**  South Africa | **Boundary Shelter**  Small inland overhang rock shelter/hunter-gatherer/hunter-herder  [7, 8] | 2160±50  Gr-A13564  **fibre from potsherd** | 206 – 63 BC | - | 1 | 2 – 3 | 1 | - Pottery with mineral and grass inclusion. |
| **CLK**  South Africa | **Clarke’s Shelter**  Inland LSA rock shelter/hunter-gatherer  [9, 10, 11] | 2160±50  Pta-2971 | 206 – 63 BC | - | 3 | 2 – 3 | 1 | - Early thin-walled ware: 7.78mm thick, bag-shaped, undecorated. |
| **UNID** | **Uniondale**  Inland LSA rockshelter/hunter-gatherer  [12] | 2130±60  2125±55 | Pta-1803  Pta-1804 | - | 3 | 2 - 3 | 1 | - Early thin-walled ware: well baked, some decoration with shallow grooves, mostly globular shaped. |
| **SPR**  South Africa | **Spoegrivier**  Coastal LSA cave site/pastoralist  [13, 14, 15] | 2105±65  **Sheep AMS** | 197 BC – AD 19 | - | 1 | 2 – 3 | 2 | - 1 (M) sheep |
| **SIT**  Zambia | **Situmpa**  Inland open-air site/farmer  [16, 17] | 2110±50  Pta-2486  1930±100  1845±212  C-829 | 196 BC – AD 15  38 BC – AD 240  51 BC – AD 436 | - | 3 | 2 – 5 | 1 | - Situmpa ware (Channel Decorated Ware): thick-walled, generally bag-shaped with a band of channel decoration below thickened rolled-over rim. |
| **MES**  Namibia | **Messum 1**  Inland LSA rock shelter  [3, 18, 19] | 2070±90  KN-I637  2090±45  KN-I639 | 195 BC – AD 67  134 BC – AD 18 | - | 3 | 2 – 3 | 5i | - No description of pottery - Fe loops (perhaps intrusive) - Pits |
| **FSR**  Namibia | **Falls Rockshelter**  Inland LSA rock shelter/Hunter-herder  [20, 21] | 2040±50  Pta-2930  2100±50  Pta-2929  1880±50  Pta-2927 | 145 BC – AD 18  57 BC – AD 52  AD 121 – 236 | - | 3 | 3 – 4 | 3 | - Possible ripple ware, globular pots and bowls, undecorated and decorated with punctates or incisions. - 2 (M) sheep. - The date 1880±50 (Pta-2927) was taken from a dung layer at the site. |
| **ORU**  Namibia | **Orunwanje 95/1**  Inland LSA rock shelter  [22, 23] | 2208±46  KN-5005  2165±35  KN-5304  2140±40  KN-5298  2005±35  KN-5300 | 355 – 143 BC  204 – 74 BC  197 – 63 BC  46 BC – AD 73  *Terminus post quem* 1912±27 | *Terminus post quem*  114 BC – AD 204  (noted by author) | 3 | 3 – 4 | 3? | - Pottery 5mm thick, quartz and mica with ground potsherds, grey to red in colour with horizontal grooves and herringbone decoration. Rounded bases, some incised decorations. Handle or spout fragment and bowl fragment from the lower deposits. - Sheep remains were found in levels 16 (3101±37; UtC-5587, charcoal) and level 12 (undated but post 2208±46; KN5005 from level 13); goat (3101±37; UtC-5587; charcoal, level 16). The dating of livestock remains at the site is problematic. |
| **N2005/2**  Namibia | **N2005/2**  Inland LSA rock shelter  [24] | 2090±35  KN-5798 | 110 BC – AD 17 | - | 3 | 3 | 1 | - Thin-walled (4.96mm) pottery, quartz inclusion, parallel lines and/or horizontal grooves, dark brown in colour. |
| **TOT1**  Botswana | **Toteng 1**  Inland open-air site/hunter-herder/pastoralist  [6, 25, 26, 27] | 2070±40  Beta-1904888  **cow AMS**  2020±40  Beta-186669  **sheep AMS**  1990±60  Beta-44963 | 93 BC – AD 24  48 BC – AD 58  46 BC – AD 118 | - | 1  (2 AMS dates only related to livestock remains and not to pottery) | 3 – 4 | 3 | - Bambata A: “The dated Bambata sherd is more recent than the earliest AMS-dated livestock at Toteng1. This evidence (including 3 AMS-dated potsherds supported by 2 bulk charcoal dates, suggests that the livestock arrived in the area before the Bambata pottery. However, we remain cautious about this conclusion” [26: 139). - Sheep, cattle |
| **AIT**  South Africa | **/Ai Tomas**  Inland boulder site/pastoralist  [13] | 1980±120  Pta-5530 | 66 BC – AD 225 | - | 3 | 3 – 4 | 3i | - Cape coastal ware: lugs present, decoration occurs beneath the rims with impressed horizontal lines, roughened black exterior and interior, 5.4mm thick, gritty with mica inclusions. - 2 (M) sheep/goat - Pits |
| **BOR**  South Africa/Swaziland | **Border Cave**  Inland LSA rock shelter  Hunter-gatherer  [10, 28] | 2010±50  Pta-506 | 49 BC – AD 72 | - | 3 | 3 – 4 | 1 | - Early thin-walled ware. |
| **HAA**  South Africa | **Haaskraal Shelter**  Inland LSA Rock shelter/hunter- herder  [7, 8] | 1970±50  Gr-A13541  **fibre from potsherd**  1920±130  Gr-A13557  **fibre from potsherd** | 48 BC – AD 326  37 BC – AD 127 | - | 1 | 3 – 5 | 1 | - Pottery with mineral and grass inclusion. |
| **DK1**  South Africa | **Die Kelders**  Coastal LSA rock shelter/pastoralist  [29] | 1960±95  Gx-1687 | 46 BC – AD 206 | - | 3 | 3 – 4 | 1 | - Thin-walled pottery, quartz inclusion, globular or bag shaped; undecorated, black with red-slip. - Sheep bone was recovered from level 7/9 associated with a radiocarbon determination (GX-2687), however AMS dates on sheep bone from level 7 indicate that the remains are younger. |
| **GED**  Namibia | **Geduld**  Inland LSA rockshelter/hunter-gatherer/pastoralist  [30, 31] | 1980±50  Pta-4413 | 41 BC – AD 121 | - | 3 | 3– 4 | 3? | - Ripple ware: thin-walled, spouts, undecorated and corrugated ware, burnishing is present (Bambata A). - Sheep/goat: Levels 7 and 8 produced sheep-size medium bovids but specific identification of sheep bones was possible only in Level 4. Level 4 remains undated [30:6]. - Upper grindstone |
| **OBP**  South Africa | **Olieboomspoort**  Inland LSA rock shelter  hunter- gatherer  [32] | 2000±25  Pta-8030 | 40 BC – AD 71 | - | 3 | 3 – 4 | 1 | - Bambata A pottery: thin-walled, jars, spout and bosses, grey to black with red slip, comb-stamped and incised, ripple rims. Happy Rest, Eiland, Broadhurst and Icon/Moloko pottery were found in the same stratigraphic levels as Bambata A pottery at different frequencies. |
| **BLM**  South Africa | **Blombos**  LSA rockshelter/hunter-gatherer/hunter-herder  [33, 34] | 1960±50  OxA-4543  **sheep AMS**  1880±55  OxA-4544  **sheep AMS** | AD 23 – 198  AD 116 – 243 | - | 1 | 3 – 4 | 3 | - Undecorated pottery with quartz inclusions. - 3 (M) sheep. |
| **APL**  Namibia | **Apollo 11 Rockshelter**  Inland LSA cave site/hunter-gatherer  [18, 35, 36] | 1960±45  Pta-1918 | AD 25 – 195 | - | 2 | 3 – 4 | 1 | - Early thin-walled ware. |
| **CHG1**  Mozambique | **Changlane I**  Inland open-air site/farmer  [2] | 1930±50  **carbon dated pottery AMS** | AD 63 – 203 | - | 1  The pottery has been classified as Matola 2 ware which post-dates the AMS date. | 4 | 1 | - Matola 2 type pottery: bowls with everted rims. - Stone tools were found associated with pottery at the site. |
| **OMU**  Namibia | **Omungunda 99/1**  Inland LSA rock shelter/herding  [23] | 1940±22  KIA-16045  1842±25  KIA-11982 | AD 70 – 125  AD 147 – 248 | - | 2 | 4 | 1 | - Pottery 5mm thick, quartz and mica with ground potsherds, grey to red in colour, horizontal grooves and herringbone decoration and ‘Ripple’ Ware: quartz inclusion, thin-walled, 5.5mm thick. |
| **REN**  South Africa | **Renbaan**  Inland LSA cave site  [37] | 1910±60  Pta-3783 | AD 75 – 216 | - | 3 | 4 | 1 | - Cape coastal ware: undecorated and black. |
| **BUZ**  South Africa | **Buzz Shelter**  Inland LSA rock shelter/hunter-gatherer  [38] | 1921±25  OxA-24515 | AD 78 – 202 | - | 3 | 4 | 2 | - 1 (M) sheep |
| **DEP**  Botswana | **Depression Cave**  Inland cave site/hunter-gatherer  [39, 40] | 1860±90  Beta-22878 | AD 79 – 339 | - | 3 | 4 – 5 | 1 | - Undecorated pottery.   Pottery was associated with stone tools at the site. |
| **VOL**  South Africa | **Volstruisfontein**  Inland rock shelter/hunter/herder  [7, 8, 41] | 1890±50  Gr-A13560  **fibre from potsherd** | AD 116 – 232 | - | 1 | 4 | 1 | - Pottery with mineral and grass inclusion. |
| **HAW**  South Africa | **Hawston**  coastal shell midden/strandlooper  [42, 43] | 1900±40  Pta-835  1860±60  Pta-834 | AD 116 – 216  AD 120 – 322 | - | 3 | 4 – 5 | 3 | - Pottery with lugs or boss, red in colour, undecorated, thin-walled, conical bases. - 1 (M) sheep |
| **KBA**  South Africa | **Kasteelberg A**  Coastal boulder site/hunter-herder/Pastoralist  [44,45, 36, 46, 47, 48] | 1860±60  Pta-3711  1790±40  Pta-3461 | AD 120 – 322  AD 243 – 342 | - | 2 | 4 – 5 | 3 | - Pottery 6mm thick, jar-shaped, spout, incised decoration and undecorated, red slip (Cape coastal pottery). - Sheep - Upper grindstones stained with ochre |
| **COL**  South Africa | **Colwinton**  Inland LSA rock shelter/hunter-gatherer  [49, 10] | 1890±45  Pta-2549 | AD120 – 224 | - | 3 | 4 | 1 | - Early-thin walled ware: 7mm wall-thickness, grit with some grass inclusions, undecorated, red to brown in colour. |
| **MAT**  Mozambique | **Matola IV**  River terrace midden site/farmer  [50, 51] | 1880±50  R -1 327 | AD 121 – 236 | - | 2 | 4 | 6 | - Matola 1 type pottery: fluted rims, single bands of horizontal line incisions with diagonal or cross-hatched incisions on shoulder and body, thickened rims, between 7-8mm thick, discontinuous motif on body of pot. - Fe artefacts, Fe slag. |
| **SKE**  South Africa | **Skeurkrans Shelter**  Inland LSA rock shelter/hunter-gatherer  [32, 52, 53] | 1850±50  Pta-5161 | AD 128 – 321 | - | 3 | 4 – 5 | 1 | - Bambata A and Ripple ware. |
| **ZIT**  Mozambique | **Zitundo**  Coastal hilltop site/farmer  [50] | 1760±105  St-8911  1775±85  St-8909  1685±105  St-8912 | AD 129 – 517  AD 247 – 362  AD 245 – 573 | - | 3 | 4 – 6 | 6 | - Matola 1 type pottery: fluted rims, single bands of horizontal line incisions with diagonal or cross-hatched incisions on shoulder and body, thickened rims 7-8mm. discontinuous motif on body. - Fe slag |
| **N2**  Namibia | **N2000/2**  LSA rock shelter  [23] | 1850±40  KIA-17709 | AD 130 – 249 | - | 3 | 4 | 1 | - Early thin-walled ware: 4mm, black to red sherds. |
| **RIA**  Mozambique | **Rian Rockshelter**  Inland rock shelter with farmer pottery  [54] | 1870±35  ICEN-133 | AD 131 – 233 | - | 3 | 4 | 6 | - Early farmer ware (no description except that it belongs to Kwale Tradition). - Fe artefact |
| **PHO**  Malawi | **Phopo Hill**  Open-air site/farmer  [55, 56] | 1745±170  SR-148 | AD 133 – 530 | - | 2 | 4 – 6 | 6i | - Phopo ware: globular pots with everted rims and hemispherical bowls with in-turned rims, graphite burnish is present and channel decoration; black to grey in colour, 5 -10mm thick. - Fe bangle, Fe slag, tuyere - Daga (no identifiable floor or feature) - Human figurine (not associated to radiocarbon date). |
| **SNR**  Namibia | **Snake Rock**  Inland LSA rock shelter/hunter-herder  [57] | 1840±50  Pta-2886 | AD 134 – 330 | - | 3 | 4 – 5 | 1 | - Ripple ware: globular pots and bowls; undecorated and decorated with punctates or incisions. |
| **JUB**  South Africa | **Jubilee Shelter**  Inland LSA rock shelter/hunter-gatherer  [58, 15] | 1840±50  Wits-1398 | AD 134 – 330 | - | 2 | 4 – 5 | 1 | - Bambata A: thin-walled (3.2-.9mm), 114 undecorated, 7 decorated with stamped impression, occasionally cross-hatching. - A single sheep bone occurs in the same layer as the earliest occurrence of Bambata A pottery but it is directly (AMS) dated to 310±50 (OxA-6039) (Sealy & Yates 1994). |
| **EGH**  South Africa | **Edgehill**  Inland LSA rock shelter/hunter-gatherer  [59] | 1830±60  Pta-3564 | AD 136 - 339 | - | 3 | 4 – 5 | 1 | - Albany ware: well-baked, thin-walled, globular-shaped pots with shallow grooved or impressed bands, red burnish occurs on some pots. |
| **ORU**  Namibia | **Orunwanje 95/1**  Inland LSA rock shelter/herding  [22, 23] | 1924±50  KN-4848  1950±35  KN-5303  1907±27  KN-5003  1900±40  KN-5302 | AD 68 – 204  AD 49 – 195  AD 116 – 205  AD 116 – 216 | AD 146 | 2 | 4 | 1 | - Pottery 5mm thick; quartz and mica with ground potsherds; grey to red in colour with horizontal grooves and herringbone decoration. Rounded bases, some with incised decoration. Handle or spout fragment and bowl fragment from the lower deposits. |
| **SPR**  South Africa | **Spoegrivier**  Coastal LSA cave site/pastoralist  [13, 14, 15] | 1900±50  GrA-9032  **sheep AMS**  1900±50  GrA-9028  **sheep AMS**  1890±50  GrA-9092  **sheep AMS** | AD 85 – 221  AD 85 – 221  AD 116 – 232 | AD 163 | 1 | 4 | 2 | - 3 (M) sheep |
| **LUM**  Mozambique | **Lumbi**  Inland open-air site/farmer  [60] | 1900±30  UA-40071  1892±30  UA-40070 | AD 121 – 205  AD 126 – 206 | AD 165 | 2 | 4 | 1 | - Matola type pottery: fluted rims, single bands of horizontal line incisions with diagonal or cross-hatched incisions on shoulder and body, thickened rims 7-8mm; discontinuous motif on body of pot**.**   LSA stone tools are represented at this time period. |
| **TOT1**  Botswana | **Toteng 1**  Inland open-air site/hunter-herder/pastoralist  [25, 6, 26, 17] | 1820±50  Pta-5534 | AD 170 – 340 | - | 2 | 4 – 5 | 3 | - Bambata A pottery: 4-6mm thick; mostly jars; necked vessels and bowls, black to grey in colour, multiple bands of alternating designs, comb-stamping more common than incision, cross-hatching and punctates are also present. - Sheep, cattle |
| **MTT**  Zambia | **M’teteshi**  Inland open-air site/farmer  [61] | 2030±90  NZ-4520  1810±80  NZ-4518 | 105 BC – AD 119  AD 144 – 364 | AD 176 | 3 | 4 | 5 | - Pottery with thickened rims, single band of chevron motifs and/or multiple lines. Punctates main decoration technique. Comb-stamping less frequent. - Fe bangle fragment, Fe razor, Fe spear point, Fe awl, Fe lump, Fe slag (few pieces) - Cow peas and sorghum sp. (not associated with radiocarbon dates) - Daga was recovered and was not associated with Unit P where the radiocarbon dates were derived. |
| **REC**  South Africa | **Reception Shelter**  Inland LSA small rock shelter  [38] | 1897±25  OxA-25353  1840±26  OxA-25354 | AD 124 – 205  AD 145 – 249 | AD 181 | 3 | 4 | 1 | - Undecorated pottery |
| **GED**  Namibia | **Geduld**  Inland LSA rock shelter/Hunter-herder/pastoralist  [30, 31] | 1790±80  Pta-4419 | AD 205 – 387 | - | 2/3  Sheep/goat remains found above this date. | 4 – 5 | 3? | - Ripple ware: thin-walled, spouts, undecorated and corrugated ware, burnishing is present (Bambata A) - Sheep/goat - Upper grindstone |
| **BLY**  South Africa | **Blydefontein**  Inland large rockshelter/hunter– herder  [62, 63,64, 65, 66, 67, 68] | 1810±50  GrA-15192 | AD 212 – 344 | - | 2 | 4 – 5 | 1 | - (1) Fibre tempered pottery: described as grass tempered plain ware mostly shaped into bowls or bag-shaped pots. (2) undecorated thin-walled ware described as silt tempered, coil-built pottery with grass and silt temper. (3) Khoe pottery occurs in more recent layers. |
| **BEF**  Angola | **Benfica**  Coastal shell midden site/farmer  [69, 70, 52, 6] | 1810±50  Pta-212 | AD 212 – 322 | - | 3 | 4 – 5 | 1 | - Kalundo Tradition pottery: decorated with cross-hatching, stamping, herringbone, parallel lines and multiple bands; along with parallel lines on the neck; and alternating blocks of lines both in the neck and on the shoulder.   LSA stone tools represented at the site |
| **UNC**  Mozambique | **University Campus**  Open-air site/farmer  [71] | 1775±85  St-9836 | AD 214 – 410 | - | 2/3 | 4 – 5 | 6i | - Matola 1 pottery: fluted rims, single bands of horizontal line incisions with diagonal or cross-hatched incisions on shoulder and body, thickened rims, 7-8mm wall-thickness, discontinuous motif on body. - Fe artefacts, Fe slag - Pits associated with smelting debris, daga remains (no identifiable floor or feature). |
| **TOR**  South Africa | **Tortoise Cave**  Coastal LSA cave site/hunter-gatherer  [72] | 1800±60  Pta-5616  1780±50  Pta-5615 | AD 217 – 361  AD 245 – 360 | - | 2 | 4 – 5 | 1 | - Undecorated pottery; 5.5 – 12.5mm thick; black-brown to red in colour. |
| **BLO5**  South Africa | **Bloubos 5 BLO5**  Inland LSA surface scatter  [73, 74] | 1810±45  Pta-7381  OES | AD 218 – 340 | - | 3  Date has not been corrected. | 4 – 5 | 1 | - Pottery with lugs; undecorated; red sherds; thin-walled (5.6mm), grit tempered. |
| **MTGL**  Malawi | **Mitongwe/Liwadzi**  Inland open-air site/farmer  [75] | 1730±90  SR-209 | AD 236 – 464 | - | 3 | 4 – 5 | 1 | - Nkope type pottery. |
| **MAT**  Mozambique | **Matola IV**  River terrace midden site/farmer  [50, 51] | 1720±110  St-8547 | AD 242 – 518 | - | 2 | 4 – 6 | 6 | - Matola 1 pottery: fluted rims, single bands of horizontal line incisions with diagonal or cross-hatched incisions on shoulder and body, thickened rims, 7-8mm wall-thickness, discontinuous motif on body. - Fe artefacts, Fe slag |
| **JKKM**  South Africa | **Jakkalsberg M**  Inland open-air site/hunter-herder  [38] | 1740±75  GX-32760  OES | AD 246 – 417 | - | 3  Date has not been corrected. | 4 – 5 | 3 | - Pottery with horizontal incised decoration, 115 sherds have red slip, 3 lugs and 2 bosses present. - 1 (M) sheep, 2 (M) sheep/goat - Upper grindstone ochre stained, lower grindstone |
| **CAE**  Botswana | **Cae Cae (Xai-Xai)**  Inland open-air pan site/hunter-gather with farmer and herder items  [76, 77] | 1765±55 | AD 247 – 372 | - | 3 | 0 | 5 | - Early thin-walled ware: charcoal and calcrete temper with incised decoration [77]; Yellen and Brooks [78] note that the pottery is largely undiagnostic and thumbnail sized. - Fe bead (the bead is not directly associated with the radiocarbon date and has possibly been displaced in the deposit) |
| **KAMU**  Zimbabwe | **Kamukombe**  Inland open-air site/farmer [78] | 1785±15  Pta-9642 | AD248 – 338 | - | 2 | 4 – 5 | 1 | - Kadzi ware: thick profiles, out-turned lips (decorated), rims with comb-stamping. |
| **SIT**  Zambia | **Situmpa**  Inland open-air site/farmer  [16, 17] | 1710±100  SR-40 | AD 248 – 517 | - | 3 | 4 – 6 | 1 | - Situmpa ware (Channel Decorated Ware): thick-walled, generally bag-shaped with a band of channel decoration below thickened, rolled-over rim. |
| **KBDe**  South Africa | **Kasteeberg De**  Coastal boulder site/hunter-herder  [47, 48] | 1760±50  Pta-8095 | AD 249 – 370 | - | 3 | 4 – 5 | 3 | - Pottery that has 6mm wall-thickness, jars, spout, incised decoration, less undecorated sherds, spouts and lugs present, red slip. - Total at site: 963 (N) sheep and sheep/goat, 57 (N) cattle (livestock occur in all levels at the site) |
| **DRL**  South Africa | **Driel Shelter**  Inland LSA rock shelter/hunter-gatherer  [10, 11, 79] | 1775±40  Pta-1381 | AD 249 – 356 | - | 2 | 4 – 5 | 1 | - Early thin-walled ware: black to grey in colour, 8.21 mm thick, some are bag-shaped, undecorated. |
| **SIL**  South Africa | **Silver Leaves**  Inland open-air site/farmer  [80, 81, 82] | 1760±50  Pta-2360 | AD 249 – 370 | - | 2 | 4 – 5 | 7i | - Silver Leaves: fluted bowls, beveled jars, single line punctates or incision on lip and rim, discontinuous motifs on body of pot. Pot with impressed bulrush millet* (pennisetum) - Fe artefact (unidentified), Fe slag, tuyere - Bulrush millet* - Pits, daga (no identifiable floor or feature) |
| **ERO**  Namibia | **Eros**  Inland LSA rock shelter/hunter-gatherer  [35, 36] | 1745±35  GrN-5297 | AD 253 – 385 | - | 3 | 5 | 1 | - Early thin-walled pottery |
| **MAL**  Mozambique | **Malessane**  Inland rock shelter with farmer pottery  [54, 83] | 1740±40  ICEN-132 | AD 253 – 390 | - | 3 | 5 | 1 | - Nkope pottery - Fe slag |
| **SHH**  Lesotho | **Sehonghong**  Inland LSA rock shelter/hunter-herder  [84] | 1710±50  Pta-6063 | AD 254 – 425 | - | 2 | 5 | 1 | - Early thin-walled pottery |
| **ORU**  Namibia | **Orunwanje 95/1**  LSA rockshelter/herding  [22, 23] | 1731±43  KN-4850  1731±43  KN-4850 | AD 255 – 409 | - | 2 | 5 | 3 | - Ripple rim ware(?) with coarse quartz inclusion, 5.7mm thick, incised lines, red to brown in colour. - Goat |
| **DUI**  South Africa | **Duiker Eiland**  Coastal shell midden site/herder  [35, 85, 86] | 1700±50  Pta-1581  Shell | AD 255 – 439 | - | 3  Pottery and shell (both from the surface) were not associated in the layer and it could be that pottery post-dates shell. | 5 | 1i? | - No description of pottery besides a spout present - Stone features: Feature G from Duiker Eiland comprises a ring of stones (most likely a hearth feature) a date underlying the feature was given 1970±75 (Pta-1707). Another stone feature possibly a floor perhaps associated with a charcoal sample from a hearth in the centre (2280±45 (Pta-1709). The correlation between dates and stone features in the area however are not secure. |
| **OMU**  Namibia | **Omungunda 99/1**  Inland LSA rock shelter/herding  [23] | 1739±23  KIA-16046  1705±35  KN-5641 | AD 257 – 385  AD 343 – 422 | - | 2 | 5 | 5 | - Ripple ware, quartz inclusion, thin-walled (5.5mm), horizontal incisions, black to grey and red sherds. - Fe blade and awl. |
| **OBP**  South Africa | **Olieboomspoort**  Inland LSA rock shelter/hunter-gatherer  [32] | 1700±45  Pta-7798 | AD 257 – 434 | - | 2/3 | 5 | 1 | - Bambata A pottery: thin-walled; jars; spout and bosses, grey to black in colour with red slip, comb-stamped and incision, ripple rims. Happy Rest, Eiland, Broadhurst and Icon/Moloko pottery were found in the same stratigraphic levels as Bambata A pottery at different frequencies. |
| **DIK1**  South Africa | **Dikbosch 1**  Inland LSA rock shelter/hunter-herder/ pastoralist  [87] | 1720±40  Pta-3413 | AD 258 – 415 | - | 3 | 5 | 3? | - Doornfontein type pottery: grit tempered, relatively thin-walled (6-7mm), undecorated - Sheep/goat |
| **LMR1/2**  South Africa | **Limerock 1&2**  Inland LSA small rock shelter/hunter-herder/ pastoralist  [87, 88] | 1720±40  Pta-2095 (LMR2)  1620±50  Pta-1621 (LMR1) | AD 258 – 415  AD 422 – 536 | - | 3 | 5 – 6 | 3ii? | - Doornfontein type pottery: grit tempered, relatively thin-walled (6-7mm), undecorated and ‘undulating effect’ (ripple ware?). - 2 (M) sheep/goat - Sheep/goat enclosure (association to radiocarbon determination not clear), low stone walling (association to radiocarbon determination not clear) |
| **CLH**  South Africa | **Collingham Shelter**  Inland LSA rock shelter/hunter-gatherer  [10, 11, 88] | 1880±45  Pta-5101  1810±60  Pta-5262  1800±50  Pta-5096  1770±50  Pta-5274 | AD 125 – 228  AD 205 – 360  AD 227 – 356  AD 248 – 362 | AD 264 | 2 | 5 | 5 | - Early thin-walled pottery: bag shaped; black to grey in colour, undecorated, thin-walled (5.88mm). - Fe bead, Cu bead |
| **TOR**  South Africa | **Tortoise Cave**  Coastal LSA cave site/hunter-gatherer.  [72] | 1680±50  Pta-3312  1620±50  Pta-3310  1610±50  Pta-3311 | AD 361 – 517  AD 422 – 536  AD 425 - 541 | - | 2 | 5 – 6 | 3 | - Undecorated pottery, 5.5 – 12.5mm thick; black-brown to red in colour. - Total at site: 13(M) sheep |
| **LUS**  Zambia | **Lusu**  Inland LSA site with farmer pottery  [16, 17] | 2025±230  C-830 | After 355 BC – AD 317 | - | 3 | 5? | 1 | - Situmpa Ware or Channel Decorated Ware. |
| **LOT**  Botswana | **Lotshitshi**  Inland open-air site/hunter-herder  [90, 91] | 1660±100 | AD 340 – 572 | - | 3 | 5 – 6 | 3 | - Bambata A pottery: 4-6mm thick; mostly jars; necked vessels and bowls, black to grey in colour, multiple bands of alternating designs, comb-stamping more common than incision, cross-hatching and punctates. - Cattle |
| **PHO**  Malawi | **Phopo Hill**  Open-air site/Farmer  [55, 56] | 1655±95  SR-128 | AD 344-572 | - | 2 | 5 – 6 | 6i | - Phopo ware: globular pots with everted rims and hemispherical bowls with in-turned rims, graphite burnish is present and channel decoration; black to grey in colour, 5 -10mm thick. - Fe bangle, Fe slag, tuyere - Daga (no identifiable floor or feature) - Human figurine (not associated to radiocarbon date). |
| **SMF**  Zambia | **Samfya Forest**  Inland open-air site/farmer  [92] | 1600±80  N-1935 | AD 357 – 544 | - | 3 | 5 – 6 | 6i | - Kalambo type ware (Channel Decorated Ware). - Cu wire, Fe slag - Pits, post-hole impressed daga |
| **SIL**  South Africa | **Silver Leaves**  Inland open-air site/farmer  [80, 81, 82] | 1680±60  Pta-901 | AD 359 – 520 | - | 2 | 5 – 6 | 7i | - Silver Leaves: fluted bowls, beveled jars, single line punctates or incision on lip and rim, discontinuous motifs on bod of poty. Pot with impressed bulrush millet (pennisetum) - Fe artefact (unidentified), Fe slag, tuyere - Bulrush millet - Pits, daga (no identifiable floor or feature) |
| **PBA**  South Africa | **Pearly Beach Area**  Coastal midden site/strandlooper  [93] | 1680±50 | AD 361 – 517 | - | 3 | 5 – 6 | 3i | - Undescribed pottery - Sheep present (no faunal list provided) - Stone feature (circular probable hearth) - Upper grindstones |
| **NAU**  South Africa | **Nauga 1**  Inland ochre mine site  [94] | 1680±50  Pta-4506 | AD 361 – 517 | - | 3 | 5 – 6 | 1 | - Doornfontein type pottery: grit tempered, relatively thin-walled (6-7mm), undecorated. |
| **EIL**  South Africa | **Eiland Salt Works**  Inland open-air salt working site/farmer  [52, 95] | 1680±40  Pta-1524 | AD 363 – 471 | - | 2 | 5 – 6 | 1i | - Mzonjani pottery - Daga |
| **KBE**  South Africa | **Kasteelberg E**  Coastal boulder site/hunter-herder/pastoralist  [48] | 1670±60  Pta-7452 | AD 368 – 520 | - | 3 | 5 – 6 | 1 | - Pottery with 6mm wall-thickness, jar, spout, incised decoration; less undecorated sherds; red slip (Cape coastal pottery). |
| **APL**  Namibia | **Apollo 11 Rockshelter**  Inland LSA cave site/hunter-gatherer  [35, 36] | 1670±55  KN-1870 | AD 369 – 519 | - | 2 | 5 – 6 | 1 | - Early thin-walled pottery |
| **CHO**  Zambia/Malawi Border | **Chowo River**  Open-air river site/ farmer  [96] | 1670±40  Beta-182951  1600±40  Beta-182957 | AD 373 – 516  AD 435 – 543 | - | 2 | 5 – 6 | 7i | - Chifumbaze Complex type pottery: simple stamped impressions in horizontal bands, thickened rounded rims. - Fe rusted clumps - Sorghum bicolor - Daga |
| **MZJ**  South Africa | **Mzonjani**  Coastal midden site/farmer  [97] | 1670±40  Pta-1980 | AD 373 - 516 | - | 3 | 5 – 6 | 6i | - Mzonjani pottery: pots with straight or everted necks, single horizontal grooved decoration (impressions or incisions) below lip; discontinuous body decoration (‘wavy’, zig-zag, chevron designs), bowls: thick, sub-carinated with horizontal grooved decoration below carination. - Fe slag, tuyere - Pit lined with potsherds |
| **MAU**  Botswana | **Maunatlala**  Inland open-air site/farmer  [90] | 1570±140 | AD 381 – 651 | - | 2 | 5 – 6 | 7i | - Bambata B pottery - Metal use - Pole-impressed daga structures, grain-bins   Carbonized cucurbits present at the site. |
| **SNR**  Namibia | **Snake Rock**  Inland LSA rock shelter/hunter-herders  [21, 57] | 1640±70  Wits-1249 | AD 385 – 543 | - | 3 | 5 – 6 | 3i | - Ripple ware(?): globular pots and bowls; undecorated and decorated with punctates or incisions. - 1 (M) sheep - Stone-lined pits.   Slivers of suspected sheep bone found (possibly used as decoration). |
| **RUU2**  Namibia | **Ruuga 2**  Inland open-air site/Iron working groups  [98, 99] | 1602±123  KIA-28854  **charcoal extracted from pottery** | AD 385 – 630 | - | 2 | 5 – 6 | 6 | - Pottery similar to Divuyu and Bambata - Fe working |
| **DK1**  South Africa | **Die Kelders**  Coastal LSA rock shelter/pastoralist  [29] | 1600±120  GaK-3955  1590±80  Gak-3959  1480±55  UW-291  1465±100  Gx-1685 | AD 387 – 630  AD 424 – 586  AD 576 – 656  AD 520 – 765 | - | 3 | 5 – 6 | 1 | - Thin-walled pottery: quartz inclusion, globular or ba- shaped, undecorated, black with red-slip. - 18 (M) sheep, 2 (M) cattle   The dates provided here were obtained from the more recent layers at the site, however, directly dated sheep remains from an older layer (7) are dated earliest to 1325±60 (OxA-3860) and calibrated to AD 670 – 840 (Group 7). The relationship between these 4 radiocarbon determinations and the material is not secure. |
| **KAM**  Zambia | **Kamnama**  Inland open-air site/farmer  [100] | 1600±110  KN-908 | AD 390 – 627 | - | 3 | 5 – 6 | 6i | - Kamnama type ware (only a few potsherds): grooved or stamped ware with some traces of burnishing. Mostly necked vessels. - Fe strip, Fe slag, tuyere - Daga (no identifiable floor or feature) - Lower grindstone |
| **GZP1a**  Zimbabwe | **Great Zimbabwe P1a**  Inland stone walling site  [52] | 1630±75  M913 | AD 390 – 570 | - | 2 | 5– 6 | 1i | - Bambata B pottery: 6-10mm thick, multiple band decoration on neck and shoulder and Ziwa pottery present. - Daga |
| **NKP**  Malawi | **Nkope Bay**  Open-air site/farmer  [56, 101, 102] | 1590±120  SR-174 | AD 392 – 636 | - | 3 | 5 – 6 | 9 | - Nkope pottery: globular-shaped pot with a narrow thickened and everted rim. Decoration, apart from on the rim-band or a band of channeling immediately below it is rare but may occur on the shoulder. Angular motifs are usual, curvilinear designs are less common and when they do occur they tend to be restrained in form. The bowls represent about 80 % of the total vessels at Nkope Bay (63 %), thickened and in-turned (33 %) and rounded. The two former types are usually decorated. This particularly applies to the in-turned rims, which are nearly always covered with horizontal channeling or fluting, usually combined with impressed or incised decoration on or below the actual rim. In addition, molded bosses sometimes occur. - Fe smelting - 1 (M) cattle (single tooth)   Hunting and fishing are the main activities at the site; LSA stone tools are present. |
| **ENK**  South Africa | **Enkwazini**  Coastal midden site/farmer  [103] | 1650±50  Pta-1847  1540±60  Pta-1977 | AD 394 – 525  AD 520 – 641 | - | 2 | 5 – 6 | 1i | - Mzonjani pottery: pots with straight or everted necks, single horizontal grooved decoration (impressions or incision) below lip, discontinuous body decoration (‘wavy’, zig-zag, chevron designs), bowls: thick, sub-carinated with horizontal grooved decoration below carination - Pit |
| **KBA**  South Africa | **Kasteelberg A**  Coastal boulder site/hunter-herder/Pastoralist  [36,44, 45, 46, 47, 48] | 1630±60  OxA-3864  **sheep AMS** | AD 410 – 543 | - | 1 | 5 – 6 | 3 | - Pottery with 6mm wall-thickness, jar, spout, incised decoration, less undecorated sherds, spouts and lugs present, red slip (Cape coastal pottery) - Sheep - Upper grindstones stained with ochre |
| **RIA**  Mozambique | **Rian Rockshelter**  Inland rock shelter with farmer pottery  [54] | 1485±170  ST-9021 | AD 410 – 770 | - | 3 | 5 – 7 | 5 | - Early farmer pottery (no description except that it belongs to Kwale Tradition). - Fe artefact |
| **BPS**  South Africa | **Boomplaas**  Inland LSA cave site/hunter-gatherer and herder  [104, 105] | Between  1700±55  UW-338  1630±50  UW-337 and  1510±75  UW-307 | Between AD 254 – 463,AD 417 – 531 and AD 521 – 655 | - | 2 | 5 – 6 | 4i | - Undecorated and few decorated potsherds, spouts. - Cu bead - 12 (M) sheep - LSA storage pits, stone feature (hearth) |
| **VSS**  South Africa | **Vonk se Stal**  [48] | 1630±60  Pta-4243 | AD 410 – 543 | - | 3 | 5 – 6 | 1 | - No description of pottery but one sherd with zig-zag double line decoration. |
| **TOT1**  Botswana | **Toteng 1**  Inland open-air site/hunter-herder/pastoralist  [6, 25, 26, 27] | 1639±33  **charcoal from pottery**  1560±60  Beta-184609  1480±40  Beta-186670  **cow AMS** | AD 419 – 518  AD 474 – 628  AD 594 – 648 | - | 1 | 5 – 6 | 3 | - Bambata A pottery: 4-6mm thick, mostly jars, necked vessels and bowls, black to grey in colour, multiple bands of alternating designs, comb-stamping more common than incision, cross-hatching and punctates are present. - Sheep, cattle |
| **KLA**  South Africa | **Klein Afrika**  Inland open-air site/farmer  [52, 106] | 1630±45  Pta-1168 | AD 420 – 526 | - | 2 | 5 – 6 | 9 | - Mzonjani pottery [52] or Gokomere and Happy Rest pottery types [106]. - Fe slag, tuyere, Fe ore - Cattle, goat - Pits, pole-impressed daga |
| **OBP**  South Africa | **Olieboomspoort**  Inland LSA rock shelter/hunter-gatherer  [32] | 1610±50  Pta-8027  1600±50  Pta-8032  1530±40  Pta-7799  1520±40  Pta-7778 | AD 425 – 541  AD 431 – 586  AD 546 – 631  AD 550 – 636 | AD 544 | 2/3 | 5 – 6 | 1 | - Bambata A pottery: thin-walled, jars, spout and bosses, grey to black in colour with red slip, comb-stamped and incised, ripple rims. Happy Rest, Eiland, Broedhurst and Icon/Moloko pottery were found in the same stratigraphic levels as Bambata A pottery at different frequencies. - Sheep/goat are present but only in more recent occupation levels. |
| **UNC**  Mozambique | **University Campus**  Open-air site site/farmer  [71] | 1590±75  St-9838 | AD 427 – 583 | - |  | 5 – 6 | 6i | Total at site   - Matola 1 pottery: fluted rims, single bands of horizontal line incisions with diagonal or cross-hatched incisions on shoulder and body, thickened rims, 7-8mm wall-thickness, discontinuous motif on body of pot. - Fe artefacts, Fe slag - Pits associated with smelting debris, daga remains (no identifiable floor or feature). |
| **KSBD**  Namibia | **!Khuiseb Delta (Liz’s Midden)**  Shell midden site/hunter-herder/pastoralist  [57, 107] | 1600±50  Pta-4004 | AD 431 – 568 | - | 3 | 5 - 6 | 1 | - Pottery comprised small globular vessels, highly burnished and bearing in some cases elaborate decoration on the neck and rim. |
| **KN41**  South Africa | **KN2005/041**  Coastal shell midden/hunter-herder  [108] | 1625±25  OxA-22933  **cow horn core AMS** | AD 431 – 519 | - | 1 | 5 – 6 | 2 | - 1 (M) cattle |
| **BOK**  South Africa | **Bokvasmaak 3**  Inland open-air midden site/herder  [74, 94, 109] | 1620±35  Pta-4877  OES  120±50 (spit 2)  Pta-4872 | AD 431 – 525  AD 1700 – 1945 | - | 3  Date has not been corrected. | 5 – 6 | 1 | - Doornfontein type pottery: grit tempered, relatively thin-walled (6-7mm), undecorated. - 3 (N) sheep, 10 (N) cattle (associated with spit 2 radiocarbon date). |
| **NAMB**  Malawi | **Namaso Bay**  Open-air site/farmer  [110] | 1555±110  GX-14812 | AD 432 – 639 | - | 2 | 5 – 6 | 5i | - Nkope pottery - Fe corroded strip, Cu spirally wound wire - Post-hole daga - Upper grindstone, lower grindstone with oval groove mark |
| **HPR**  South Africa | **Happy Rest**  Inland open-air site/farmer  [52, 111] | 1600±45  Pta-2421 | AD 433 – 545 | - | 3 | 5 – 6 | 9i | - Mzonjani pottery and/or Happy Rest pottery: everted or thickened rim pots with incised, stamped or grooved decoration on the rim and neck of vessel. - Fe slag   Total livestock at site   - 8 (M) sheep, 7 (M) cattle, 4 (M) goat, 8 (M) sheep/goat - Daga |
| **KLF**  Zambia | **Kalambo Falls (Site C)**  Inland open-air hill site/farmer  [112, 113] | 1605±40  GrN-4646  1520±40  GrN-4647 | AD 434 – 539  AD 550 – 636 | - | 2 | 5 – 6 | 6i | - Kalambo pottery: channel decorated, mostly shouldered pots, hemispherical bowls, rounded rims common, channeled bands and incised cross-hatch also occur around the lip, rim and neck. Lugs, spouts and handles are not present in this ware. Two dimple bases were found from Site C. - Fe tanged or barbless spearhead or knife, Fe slag, tuyere - Pits (with bottomless pots), pole-impressed daga, hut floor - Upper grindstone, flat tabular lower grindstones |
| **OMU**  Namibia | **Omungunda 99/1**  Inland LSA rock shelter/herding  [23] | 1602±29  KIA-22463  1600±35  KN-5285 | AD 439 – 540  AD 439 – 542 | - | 2 | 5 – 6 | 5 | - Ripple ware with quartz inclusion, thin-walled (5.5mm), horizontal incisions, black to grey and red sherds. - Fe blade and awl |
| **TOR**  South Africa | **Tortoise Cave**  Coastal LSA cave site/hunter-gatherer  [73] | 1590±50  Pta-5817  1580±50  Pta-3309 | AD 440 – 575  AD 443 – 584 | - | 2 | 5 – 6 | 3 | - Undecorated pottery; 5.5 – 12.5mm thick, black-brown to red in colour.   Total livestock at site   - 13 (M) sheep |
| **KN54**  South Africa | **KN2005/054**  Coastal small shell midden  [38] | 1598±25  OxA-22932 | AD 442 – 543 | - | 3 | 5 – 6 | 1 | - Comb-stamped pottery, necked-pot, 7.08mm wall-thickness. |
| **IND**  South Africa | **Inanda Quarry**  Inland open-air site/farmer  [114] | 1580±50  Pta-5492 | AD 443 – 584 | - | 3 | 5 – 6 | 6i | - Mzonjani pottery: pots with straight or everted necks, single horizontal grooved decoration (impressions or incision) below lip, discontinuous body decoration (‘wavy’, zig-zag, chevron designs), bowls: thick, sub-carinated with horizontal grooved decoration below carination. - Fe slag - Pit - Upper grindstone, lower grindstone with elliptical grooves |
| **CLK**  South Africa | **Clarke’s Shelter**  Inland LSA rock shelter/hunter-gatherer  [9, 10, 11] | 1580±50  Pta-2973 | AD 443 – 587 | - | 2 | 5 – 6 | 1 | - Early thin-walled pottery: 7.78mm thick, bag-shaped, undecorated. |
| **CHD**  Zambia | **Chondwe**  Open-air site/farmer  [115] | 1440±160  N-998 | AD 443 – 841 | - | 3 | 5 – 7 | 1 | - Chondwe group pottery: the lower levels yielded potsherds of Early Iron Age type characterized by thick fabric, gritty or sandy paste, and thickened or undifferentiated rims. The most frequent decorative motifs are lines of false-relief chevron-stamping and designs composed of segmental blocks of comb-stamping delineated by broad grooves. A limited number of incised designs also occur. Undecorated vessels are rare. |
| **CIG**  Zimbabwe | **Cigwa Hilll**  Inland open-air site/farmer  [52] | 1540±95  SR-119 | AD 444 – 645 | - | 3 | 5 – 6 | 5 | - Gokomere pottery - Fe artefacts |
| **HAA**  South Africa | **Haaskraal Shelter**  Inland rock shelter/hunter- herder  [7, 41] | 1570±50  Gr-A13541  **Fibre in pottery** | AD 468 – 595 | - | 1 | 6 | 1 | - Pottery with mineral and grass inclusion |
| **BJP**  South Africa | **Biesje Poort**  Inland open-air site/herder  [74, 94, 109] | 1580±40  Pta-9569  1550±50  Pta-5578 | AD 472 – 582  AD 520 – 634 | - | 2 | 6 | 1 | - Pottery assemblage consisted of two spout fragments present, two decorated with incised lines and row of punctates. Burnished sherds are present, some grass tempering, relatively thin-walled (5.3mm). |
| **SAM**  Zimbabwe | **Samakande**  Open-air site/farmer  [116] | 1580±35  Ua-37563 | AD 475 – 581 | - | 2 | 6 | 6i | - Ziwa pottery - Cu beads, Fe slag - Daga (burnt) - Infant pot burial |
| **DIK1&2**  South Africa | **Dikbosch 1&2**  Inland small LSA rock shelter/hunter-herder/pastoralist  [87] | 1570±40  Pta-3412 | AD 478 – 592 | - | 3 | 6 | 3? | - Undecorated pottery - Sheep/goat |
| **174**  South Africa | **2931 CA174**  Coastal midden site/farmer | 1560±50  Pta-7577 | AD 479 – 626 | - | 3 | 6 – 7 | 1 | - Mzonjani pottery: pots with straight or everted necks, single horizontal grooved decoration (impressions or incision) below lip; discontinuous body decoration (‘wavy’, zig-zag, chevron designs), bowls: thick, sub-carinated with horizontal grooved decoration below carination. |
| **GUN**  Zambia | **Gundu**  Inland open-air site/farmer  [52, 117] | 1510±85  GX-1114 | AD 480 – 658 | - | 3 | 6 – 7 | 1i | - Kamangoza type pottery showing affiliation to Kalundu, Dambwa and Kumadzulo ware and has origins with Naviundu pottery in the Congo - Pit (?), hut floor |
| **KBJ**  South Africa | **Kabeljous**  Coastal shell midden/pastoralist  [118] | 1560±40  Pta-5982 | AD 481 – 601 | - | 3 | 6 | 3 | - No description of pottery - Sheep |
| **OMU**  Namibia | **Omungunda 99/1**  Inland LSA rock shelter/herding  [23] | 1565±35  KN-5640  1530±30  KN-5642 | AD 482 – 596  AD 548 – 629 | - | 2 | 6 | 5 | - Ripple rim ware with coarse quartz inclusion, 5.7mm thick, incised lines, red to brown sherds with some grooved horizontal and diagonal lined pottery, channel ware, incised lines, occasional cross-hatching, oblique parallel lines, black to grey to brown, 6.3mm wall-thickness, fine to medium sized quartz inclusions - Fe blade and awl |
| **KN12**  South Africa | **KN2004/012**  Coastal LSA shell midden  [38] | 1579±24  OxA-22977 | AD 483 – 580 | - | 3 | 6 | 1 | - Two decorated pot sherds: thin-walled (4.79 mm), quartz inclusion, parallel lines or horizontal diagonal incised lines, two rows of vertical impressions. |
| **EBC**  South Africa | **Elands Bay Cave**  Coastal LSA cave site/hunter-gatherer  [119, 120, 121] | 1520±80  Gak-4337 | AD 497 – 652 | - | 2 | 6 | 1 | - Lower KBB and KBA style pottery including beveled lips, shell edge-stamped decorations and spouts. |
| **SMF**  Zambia | **Samfya Forest**  Inland open-air site/farmer  [92] | 1520±80  N-1934 | AD 497 – 652 | - | 3 | 6 | 6i | - Kalambo type ware (Channel Decorated Ware) - Cu wire, Fe slag - Pits, post-hole impressed daga (?) |
| **MAV**  Zimbabwe | **Mabveni**  Inland old Kraal site/farmer  [52, 122, 123, 124] | 1770±120  SR-43  1380±110  SR-79 | AD 130 – 429  AD 599 – 857 | AD 504 | 3 | 6 | 9i | - Bambata B pottery: 6-10mm thick, multiple band decoration on neck and shoulder and Gokomere type pottery [52] or only Gokomere type pottery [121]. - Fe beads, Cu beads and rings, Fe slag - Cattle, sheep/goat - Pits, pole-impressed daga, daga floor (irregular in shape but thought to be circular), raised storage bin (uncertain) - Clay human figurine |
| **KBDe**  South Africa | **Kasteelberg De**  Coastal boulder site/hunter-herder/ Pastoralist  [46] | 1550±50  Pta-8004 | AD 520 – 634 | - | 3 | 6 | 3 | - Pottery with 6mm wall-thickness, jars, incised decoration, less undecorated sherds, spouts and lugs present, red slip and shell-stamped pottery (Cape coastal pottery).   Total livestock at site:   - 963 (N) sheep and sheep/goat, 57 (N) cattle (livestock occur in all levels at the site) |
| **MIR**  Namibia | **Mirabib Hill**  Inland LSA rock shelter site  [125] | 1550±50  Pta-1535 | AD 520 – 634 | - | 3 | 6 | 2 | - Sheep hair from dung floor - 10-20 cm dung floors |
| **PHO**  Malawi | **Phopo Hill**  Open-air site/farmer  [55, 56] | 1445±120  SR-161 | AD 520 – 771 | - | 2 | 6 – 7 | 6i | - Phopo ware: globular pots with everted rims and hemispherical bowls with in-turned rims, graphite burnish is present and channel decoration, black to grey in colour, 5 -10mm thick. - Fe bangle, Fe slag, tuyere, daga (no identifiable floor or feature) - Human figurine (not associated to radiocarbon date). |
| **KLD**  Zambia | **Kalundo Mound**  Inland open-air mound site/farmer  [17] | 1650±90  SR-65  1495±95  SR-123 | AD 361 – 572  AD 480 – 676 | AD 523 | 2 | 6 | 8i? | - Kalundo Tradition pottery: multiple band decoration on neck, shouldered vessels, thin-walled, globular shaped pots, bowls: thick-walled, spouts, lugs and bosses are present (but not numerous), burnishing present (mostly graphite around necked pots and bowls), comb-stamping common feature of decoration, increase in bag-shaped pots with increase in livestock at site. - Fe strip, Cu bracelet, Fe slag (small amount), tuyere (1 piece), bellow nozzles   Total livestock at site:   - 3(N) cattle, sheep/goat (number not provided) - Pits, daga (no identifiable floor), grain-bin (?) - Human burials with no grave goods - Clay humped cattle figurines |
| **NAMB**  Malawi | **Namaso Bay**  Open-air site/farmer  [110] | 1300±260  GX-14811 | AD 524 – 1039 | - | 2 | 6 - 8 | 5i | - Namaso pottery: sub-spherical bowls with slightly constricted mouths, open hemispherical bowls, both highly decorated with single line-impressions or comb-stamped near rim or towards shoulder in a mostly crisscross (stich) motif. - Fe corroded strip, Cu spirally wound wire - Post-hole daga - Upper grindstone, lower grindstone with oval groove mark |
| **JUB**  South Africa | **Jubilee Shelter**  Inland LSA rock shelter/hunter-gatherer  [58] | 1550±40  Wits-1381 | AD 525 – 629 | - | 2 | 6 | 1 | - Bambata A pottery: thin-walled (3.2-.9mm), 114 undecorated, 7 decorated with stamped- impression, occasionally cross-hatching. |
| **LUM**  Mozambique | **Lumbi**  Open-air site/farmer  [60] | 1558±30  Ua-40067 | AD 526 – 596 | - | 2 | 6 | 9 | - Matola type pottery: fluted rims, single bands of horizontal line incisions with diagonal or cross-hatched incisions on shoulder and body, thickened rims 7-8mm, discontinuous motif on body of pot. - Fe arrowheads, Fe knives, Fe bangle, Fe slag, - 1 (N) cow (identification is not secure), 5 (N) sheep/goat (identification is not secure) |
| **LMR1&2**  South Africa | **Limerock 1&2**  Inland LSA small rock shelter/hunter-herder/pastoralist  [87, 88] | 1620±50  Pta-1621  1430±50  Pta-1759 | AD 422 – 536  AD 600 – 680 | - | 3 | 6 – 7 | 3ii | - Doornfontein type pottery: grit tempered, relatively thin-walled (6-7mm), undecorated with ‘undulating effect’ (Ripple ware?). - 2 (M) sheep/goat, - Sheep/goat enclosure (association to radiocarbon determination is unknown), low stone walling (association to radiocarbon determination is unknown). |
| **SIM**  Zambia | **Sioma Mission**  Inland open-air site/farmer  [126] | 1498±75  N-1498 | AD 530 – 660 | - | 3 | 6 – 7 | 6 | - Early farmer pottery - Fe working, Fe smelting |
| **KLA**  South Africa | **Klein Afrika**  Inland open-air site/farmer  [52, 106, 127] | 1540±50  Pta-2415  1450±45  Pta-1321  1430±40  Pta-1320  1410±50  Pta-2420 | AD 530 – 635  AD 601 – 665  AD 630 – 678  AD 631 – 763 | - | 2 | 6 – 7 | 9i | - Happy Rest pottery - Fe slag, tuyere, Fe ore   Total livestock at site:   - 24 (N) cattle, 93 (N) sheep/goat - Pole-impressed daga (no structure identified) |
| **KAP**  Namibia | **Kapako**  Inland surface site/Iron working groups  [99] | 1550±30  POZ-20679  **charcoal extracted from pottery** | AD 531 – 600 | - | 3 | 6 | 1 | - No description of pottery |
| **CHA**  Zambia | **Chalaka**  Inland open-air site (Pit)/farmer  [61] | 1520±65  N-4995 | AD 532 - 646 | - | 3 | 6 | 1 | - Pottery is similar to the site, M’teteshi. |
| **RUU2**  Namibia | **Ruuga 2**  Inland open-air site/Iron working groups  [99] | 1565±20  KIA-28853  **charcoal extracted from pottery** | AD 534 – 580 | - | 2 | 6 | 6 | - Pottery similar to Divuyu and Bambata - Fe working |
| **LON**  Zambia | **Lonze Forest**  Inland open-air site/farmer  [16, 128] | 1481±82  C-660 | AD 535 – 676 | - | 3 | 6 – 7 | 1 | - Situmpa ware (Channel Decorated Ware) |
| **RVS**  South Africa | **Riverside**  Inland open-air site/farmer  [52, 129] | 1540±40  Pta-7591 | AD 536 – 630 | - | 2 | 6 | 3ii | - Mzonjani/Broederstroom pottery - Cattle - Pits dung lined (storage), livestock byre |
| **KAMU**  Zimbabwe | **Kamukombe**  Inland open-air site/farmer [78] | 1520±60  Pta-9638 | AD 537 – 644 | - | 2 | 6 | 3 | - Kadzi pottery: thick profiles, out-turned lips (decorated), rims with comb-stamping. - Sheep/goat (no count provided) |
| **MAT**  Mozambique | **Matola IV**  River terrace midden site/farmer  [50, 51] | 1470±80  St-8546 | AD 539 – 678 | - | 2 | 6 – 7 | 6 | - Matola type pottery: fluted rims, single bands of horizontal line incisions with diagonal or cross-hatched incisions on shoulder and body, thickened rims, 7-8mm wall-thickness, discontinuous motif on body of pot. - Fe artefacts, Fe slag |
| **GOK**  Zimbabwe | **Gokomere Tunnel Site**  Inland rock shelter site/farmer  [122, 130, 52] | 1420±120  SR-26 | AD 540 – 836 | - | 3 | 6 – 7 | 6i | - Gokomere pottery: typically, stamped ware - Fe artefacts, Cu strip, Fe slag - Pole-impressed daga - Human burial (spouted bowl found with burial) - Clay female human figurines   Wilton Type tools found in earlier excavation. |
| **LHC**  Zambia | **Leopards Hill Cave**  Inland LSA Cave site with farmer pottery  [131] | 1415±125  SR-126 | AD 541 – 842 | - | 3 | 6 – 7 | 1 | - Channel Decorated Ware |
| **KMZ**  Zambia | **Kumadzulo**  Inland open-air site/ farmer  [132] | 1529±110  N-409  1400±110  N-411  1390±110  N-410  1330±110  N-412  1260±110  N-414 | AD 439 – 650  AD 579 – 841  AD 589 – 843  AD 654 – 862  AD 682 – 957 | AD 545 | 2 | 6 | 9i | - Kumadzulo pottery with similarities to Gundu and Ndonde sites - Fe spearheads and arrowheads, one small Fe hoe, Cu ‘crosses’, Fe slag   Total livestock at site:   - 2 (M) cattle - Pits, pole-impressed daga, hut floors (sub-rectangular) - Clay figurine |
| **ZIT**  Mozambique | **Zitundo**  Coastal hilltop site/farmer  [2, 50] | 1435±105  St-8910  1490±50  **Pottery AMS** | AD 547 – 767  AD 576 – 650 | - | 2 | 6 – 7 | 6 | - Matola 2 phase pottery: larger range of decoration than Matola 1, alternating bands of incision and cross-hatching (similar to Mzonjani and Lydenburg Heads sites). - Fe slag |
| **KWC**  Zimbabwe | **Kwali Camp**  Inland open-air Fe smelting site/farmer  [133] | 1490±60  Beta-200558 | AD 548 – 653 | - | 2 | 6 | 9i | - Ziwa and Gokomere pottery - Fe blade, Fe slag, tuyere, Fe ore, furnace clay - 3 (M) sheep, 8 (M) cattle |
| **OVI**  Namibia | **Ovizorombuku 96/1**  Inland LSA rock shelter  [23] | 1525±35  KN-5295  1442±25  KIA-11985 | AD 549 – 633  AD 634 – 664 | - | 3 | 6 – 7 | 1 | - Pottery with 5.5mm wall-thickness, quartz grit, well fired, some burnished sherds (ochre and black), incised horizontal and/or oblique lines, some cross-hatching and one sherd with herringbone-like motif and another with possible finger-nail impressions. |
| **BLY**  South Africa | **Blydefontein**  Inland large rock shelter/hunter- herder  [62, 63, 64, 65, 66, 67, 68] | 1505±50  GrA-15195 | AD 552 – 645 | - | 1 | 6 | 1 | - (1) Fibre tempered pottery: described as grass tempered plain ware mostly shaped into bowls or bag-shaped pots and undecorated. (2) Undecorated thin-walled ware described as silt tempered, coil-built pottery with grass and silt temper. (3) Khoe pottery occurs in more recent layers. |
| **DK1**  South Africa | **Die Kelders**  Coastal LSA rock shelter/pastoralist  [29] | 1480±55  UW-291 | AD 576 – 656 | - | 2 | 6 – 7 | 3 | - Thin-walled pottery with quartz inclusion, globular or bag-shaped- undecorated, black with red-slip. - 1 (M) sheep |
| **SPR**  South Africa | **Spoegrivier**  Coastal LSA cave site/pastoralist  [13, 14, 15] | 1490±50  GrA-9030  **sheep AMS**  1450±50  Pta-6750  1390±50  Pta-4753 | AD 576 – 650  AD 599 – 668  AD 643 – 765 | - | 1 | 6 – 7 | 3 | - Thin-walled pottery with quartz inclusion, some horizontal grooved sherds and undecorated sherds. - 12 (M) sheep |
| **KAB**  Zambia | **Kabondo Kumbo**  Open-air river site/farmer  [134] | 1430±80  N-1918 | AD 579 – 765 | - | 3 | 6 – 7 | 6i | - Kamangoza**,** Dambwa and Kumadzulo type ware. - Fe hoes, Fe arrowheads, Fe spearheads, small Fe axe, Fe knives, Fe bodkins, Fe razors, Fe bracelet, Cu bangles, Fe slag, tuyere - Pits, pole-impressed daga, hut floor (sub-rectangular in shape) - Clay cattle figurine (?) |
| **BRD**  South Africa | **Broederstoom**  Inland open-air site/farmer  [135, 136, 137,129, 52] | 1600±50  KN-2645  1520±110  RL-351  1570±65  KN-2644  1450±100  Fra-82  1540±40  Pta-1375  1530±50  Wits-871  1490±50  UCLA-1791B  1440±50  Wits-870 | AD 431 – 568  AD 439 – 654  AD 441 – 596  AD 536 – 765  AD 536-630  AD 540 – 635  AD 576 – 650  AD 601 – 676 | AD 580 | 2 | 6 | 8i | - Mzonjani/Broederstroom pottery (reclassified from a Kalundu (western stream) origin. - Fe artefacts, Cu beads and chain links, Fe slag, tuyere, Fe working (forges), Fe ore   Total livestock at site:   - 1 (M) cow, 42 (M) sheep/goat - Pits dung-lined, post-hole impressed daga, hut floors (but no complete residential unit found), grain-bins - Human burial with grave goods, human burial with dental alteration, infant pot burial - Upper grindstones, lower grindstones with lenticular grooves |
| **HPR**  South Africa | **Happy Rest**  Inland open-air site/farmer  [52, 111] | 1480±50  Pta-2414 | AD 583 – 652 | - | 3 | 6 | 9i | - Happy Rest pottery: everted or thickened rim pots with incised, stamped or grooved decoration on the rim and neck of vessel - Fe slag   Total livestock at site:   - 8 (M) sheep, 7 (M) cattle, 4 (M) goat, 8 (M) sheep/goat - Daga - Human burial with grave goods (ivory bracelet)   A diet based mainly on herded animals. |
| **Ma38**  South Africa | **Ma38**  Inland open-air site/farmer  [138, 139, 52] | 1480±50  Pta-3725 | AD 583 – 652 | - | 3 | 6 | 3 | - Mzonjani/Broederstroom ceramic: pots with straight or everted necks, single horizontal grooved decoration (impressions or incision) below lip, discontinuous body decoration (‘wavy’, zig-zag, chevron designs), bowls: thick, sub-carinated with horizontal grooved decoration below carination. - 1 (M) sheep/goat |
| **KPW**  Zambia | **Kapwirimbwe**  Inland open-air site/farmer  [140, 141] | 1525±110  GX1013a  1540±85  GX1013b  1445±95  GX-1912 | AD 439 – 652  AD 475 – 643  AD 541 – 765 | AD 586 | 2 | 6 | 9i | - Early farmer pottery: classified as Kapwirimbwe ware with necked vessels and hemispherical bowls forming the majority of the assemblage. Decorated with horizontal bands of incised hatchings or bands of chevron designs. Rims are thickened. - Fe razor, Fe spear point and ring, large quantities of Fe slag - 2 (N) cattle - Pits, daga, post-hole impressed daga (structures could not be determined). |
| **KSBD**  Namibia | **!Khuiseb Delta (End Midden)**  Shell midden site/Hunter-herder/pastoralist  [57] | 1470±45  Pta-4049 | AD 587 – 650 | - | 3 | 6 | 1 | - The pottery comprised small globular vessels, highly burnished and bearing in some cases elaborate decoration on the neck and rim. |
| **KWK**  South Africa | **Klein Witkrans**  Inland small LSA rock shelter  [87, 88] | 1490±40  Pta-2447 | AD 589 – 645 | - | 3 | 6 | 1 | - Undecorated pottery, black in colour, grit-tempered. |
| **APL**  Namibia | **Apollo 11 Rockshelter**  Inland LSA cave site/hunter-gatherer  [18 35, 36] | 1460±55  KN-1846 | AD 590 – 666 | - | 2 | 6 – 7 | 1 | - Early thin-walled pottery |
| **CAC**  Swaziland | **Castle Cavern**  Inland Iron ore mining site  [142, 143, 144, 145] | 1550±60  Y-1712  1550±30  GRN-5315  1535±30  GRN-5022  1430±100  Y-1995 | AD 482 – 635  AD 531 - 600  AD 545 – 626  AD 536 – 765 | AD 591 | 2 | 6 | 6 | - Silver Leaves: fluted bowls, beveled jars, single line punctates or incision on lip and rim, discontinuous motifs on body of pot. - Fe smelting debris. - A few tools of LSA type [143]. |
| **LYD**  South Africa | **Lydenburg Head site**  Inland open-air site/farmer  [97, 146, 147, 148] | 1460±50  Pta-328  1410±50  Pta-1634 | AD 592 – 662  AD 631 – 763 | - | 3 | 6 – 7 | 9i | - Kalundo [146] or Matola [97, 148] type pottery - Fe beads, Fe rods, Cu beads, Fe slag, tuyere - 2 (M) sheep, 4 (M) cattle, 10 (M) sheep/goat - Dung lined pits, pole-impressed daga (no structure identified) - Human burial with dental alteration - Clay sculptured heads (therianthropic) |
| **CHO**  Zambia/Malawi Border | **Chowo River**  Open-air river site/farmer  [96] | 1480±40  Beta-182957 | AD 594 – 648 | - | 2 | 6 | 7i | - Chifumbaze Complex type pottery: simple stamped impressions in horizontal bands, thickened rounded rims. - Fe rusted clumps - Sorghum bicolor - Daga |
| **LMH**  Malawi | **Lumbule Hill**  Open-air site/farmer  [55, 56] | 1385±100  SR-147 | AD 597 – 839 | - | 3 | 6 – 7 | 6i | - Mwavarambo type ware - Fe artefacts, Fe smelting - Daga (structures not identified) |
| **KBA**  South Africa | **Kasteelberg A**  Coastal boulder site/hunter-herder /Pastoralist  [36, 44, 45, 46, 47, 48] | 1430±55  OxA-3865  **sheep AMS** | AD 598 – 681 | - | 1 | 6 | 3 | - Pottery with 6mm wall-thickness, jars, spout, incised decoration; less undecorated sherds; spouts and some lugs present, red slip, and some shell-stamped pottery (Cape coastal pottery)   Total livestock at site:   - 29 (M) sheep, 2 (M) cattle (mostly in the upper levels). |
| **EIL**  South Africa | **Eiland Salt Works**  Inland open-air salt working site/farmer  [52, 95] | 1460±40  Wits 764 | AD 599 – 654 | - | 2 | 6 | 1i | - Mzonjani pottery - Daga |
| **JP7**  South Africa | **Jagt Pan 7**  Inland open-air stone feature site/hunter-herder  [74, 94, 109] | 1430±50  Pta-4300 | AD 600 – 680 | - | 3 | 6 – 7 | 1 | - Undecorated pottery, black in colour, thin-walled (4mm). |
| **HAR**  Namibia | **Hartmann Valley 95/4**  Inland small LSA rock shelter  [23] | 1480±30  KN-5287  1407±50  KN-4851 | AD 600 – 645  AD 632 – 764 | - | 2 | 6 – 7 | 1 | - Single sherd with incised lines, other sherds show cross-hatching, black to grey to brown in colour, 5.75mm wall-thickness, fine to medium sized quartz inclusions. Most sherds recovered are undecorated. |
| **Tsh1**  South Africa | **Tsh1**  Inland open-air site/farmer  [52, 139] | 1440±50  Pta-3825 | AD 601 – 676 |  | 3 | 6 – 7 | 3 | - Mzonjani/Broederstroom pottery (reclassified from a Kalundu origin) - 29 (M) sheep/goat |
| **BES**  Namibia | **Big Elephant Shelter**  Inland LSA rock shelter/hunter-gatherer  [149, 150] | 1400 $\pm$80  UCLA-724B | AD 605 - 768 | - | 3 | 6 - 7 | 1 | - Pottery with vertical row of impressions and horizontal nail impressions, 6 -7 mm thick, mostly undecorated, greyish in colour. Spout present. |
| **ORU**  Namibia | **Orunwanje 95/1**  Inland LSA rock shelter/herding  [22, 23] | 1520±32  KN-5297  1511±33  KN-5938  1419±40  KN-5302 | AD 573 – 635  AD 580 – 637  AD 634 – 681 | AD 624 | 2 | 6 | 1 | - Ripple rim ware with coarse quartz inclusion, 5.7mm thick, incised lines, red to brown sherds. |
| **MAK**  Zambia | **Makwe**  Inland LSA rock shelter with farmer pottery  [100, 151] | 1730±110  GX-1551  1200±80  SR-107 | AD 229 – 517  AD 772 – 983 | AD 625 | 3 | 6 | 4i | - Kamnama type pottery: necked vessels, open bowls, well fired, comb-stamped, horizontal band of parallel grooves - Fe broken rods   Total at livestock at site:   - 10 (N) cattle, 2 (N) goat, - Lower grindstone |
| **RUU39**  Namibia | **Ruuga N98/39**  Inland open-air site/Iron working groups/ceramic later stone age  [99, 152] | 1440±35  KN-5577 | AD 630 – 673 | - | 3 | 6 – 7 | 6 | - Pottery similar to Divuyu and Bambata. - Fe working |
| **KAD**  Zimbabwe | **Kadzi**  Inland open-air riverside site/farmer  [78, 153, 154] | 1475±70  Ua-4106  1445±80  Ua-4105 | AD 548 – 671  AD 548 – 762 | AD 633 | 2 | 6 | 8 | - Kadzi pottery: thick profiles, out-turned lips (decorated), rims with comb-stamping - Fe artefacts, Cu artefacts, Fe slag, tuyere - 1 (M) sheep, 1 (M) cattle, c.f. 1 (M) goat, 3 (M) sheep/goat - Finger millet (Eleusine coracana) |
| **MON**  Zambia | **Mondake**  Inland open-air site (Pit)/farmer  [61] | 1510±105  NZ-4997  1355±60  NZ-4998 | AD 444 – 665  AD 656 – 768 | AD 634 | 2 | 6 | 7 | - Pottery similar to M’teteshi site - Fe ring, Fe spear, Fe bangle fragments, Fe rod, Fe razor, Fe flat fragment, Fe blade fragments, **(2b**) Cu clips - Sorghum sp. |
| **HAA**  South Africa | **Haaskraal Shelter**  Inland rock shelter/hunter- herder  [7, 41] | 1400±50  Gr-A13540  **Fibre from pot** | AD 636 – 765 | - | 1 | 6 – 7 | 1 | - Pottery with mineral and grass inclusion. |
| **BAN**  Malawi | **Banda Hill**  Open-air site/farmer  [101, 155] | 1400±50  Pta-1602 | AD 636 – 765 | - | 3 | 6 – 7 | 6i | - Nkope pottery: carinated and sub-carinated pots, 10-20mm thick, fine-grit. - Fe slag - Pits, daga (no structures identified). |
| **MLP**  South Africa | **Mhlopeni**  Inland open-air riverside site/farmer  [156, 157] | 1400±50  Pta-2878 | AD 636 – 765 | - | 3 | 6 – 7 | 9i | - Msuluzi type pottery: everted and curved necked pots with bands of decoration on neck (alternating cross-hatching, herringbone designs), body decoration is present but less common than Mzonjani ware, some burnish with red or black, bosses present, bowls are sub-carinated and thick, mostly undecorated. - Fe beads, Fe slag, 3 (M) sheep, 1 (M) sheep/goat - Human burial with sheep mandible, human burials with grindstones - Lower grindstones - Clay human figurines decorated or showing scarification, clay human figurine (hand with fingers). |
| **KGG**  South Africa | **KwaGandaganda**  Inland open-air riverside site/farmer  [158, 159, 160] | 1395±60  Wits-1918 | AD 639 – 765 | - | 2 | 6 – 7 | 9ii | - Msuluzi type pottery: everted and curved necked pots with bands of decoration on neck (alternating cross-hatching, herringbone designs), body decoration is present but less common than Mzonjani ware, some burnish with red or black, bosses present, bowls are sub-carinated and thick, mostly undecorated. - Fe beads, Cu beads, Fe slag, Fe tuyere, Fe ore - 175 (N) sheep, 63 (N) cattle, 9 (N) goat, 175 (N) sheep/goat - Pit, daga (no structure identified), livestock byre (dung deposit). “The relationship between central and residential areas suggests that the settlement was organized according to the CCP and therefore supports the position that the CCP and its associated worldview were present in South Africa by about the eighth century AD” [159: 39]. - Infant pot burial - Upper grindstones, lower grindstones - Clay horn figurines, clay human figurines, clay human figurines some decorated or scarification. |
| **CHO**  Zambia/Malawi Border | **Chowo River**  Inland open-air river site/farmer  [96] | 1410±40  Beta-182953  1370±90  Beta - 182954 | AD 640 – 755  AD 635 – 843 | - | 2 | 6 – 7 | 6i | - Chifumbaze Complex type pottery: simple stamped impressions in horizontal bands, thickened rounded rims. - Fe rusted clumps - Daga |
| **NMK**  Zambia | **Namakala**  Open-air site/ farmer  [161, 162] | 1360±80  N-2317 | AD 643 – 839 | - | 3 | 6 – 7 | 5 | - Early farming pottery - Cu wound bangle |
| **MAM**  South Africa | **Mamba**  Inland open-air riverside site/farmer  [163, 164] | 1390±50  Pta-4093  1320±50  Pta-3716 | AD 643 – 765  AD 675 – 839 | - | 2 | 6 – 7 | 9ii | - Msuluzi type pottery: everted and curved necked pots with bands of decoration on neck (alternating cross-hatching; herringbone designs); body decoration is present but less common than Mzonjani ware, some burnish with red or black, bosses present, bowls are sub-carinated and thick, mostly undecorated and Ndondondwane type pottery with similarities to Msuluzi: narrow multiple bands of decoration on neck, less decoration on body and shoulder of pot, cross-hatching more common. - Fe slag, tuyere, Fe ore, furnace wall and base - 10 (M) sheep, 37 (M) cattle, 1 (M) goat, 25 (M) sheep/goat - Pit, pole-impressed daga, livestock byre - Lower grindstone with elliptical grooves - Sculptured clay heads |
| **UNC**  Mozambique | **University Campus**  Open-air site/farmer  [71] | 1355±100  St-9837 | AD 644 – 857 | - | 2 | 6 – 8 | 6i | - Matola type pottery: fluted rims, single bands of horizontal line incisions with diagonal or cross-hatched incisions on shoulder and body, thickened rims, 7-8mm wall-thickness, discontinuous motif on body of pot. - Fe artefacts, Fe slag - Pits associated with smelting debris, daga remains (no identifiable floor or feature). |
| **JKKB**  South Africa | **Jakkalsberg B**  Inland open-air site /pastoralist  [13, 165, 166, 167] | 1380±50  Pta-6101 | AD 648 – 765 | - | 2 | 6 – 7 | 4 | - Majority of pottery undecorated with quartz inclusion, red in colour, some decoration on rim. - Fe corroded fragments - 4 (M) sheep, 12 (M) sheep/goat |
| **WIT**  South Africa | **Witklip**  Coastal LSA rock shelter/hunter-herder  [121] | 1380±50  Pta-4608 | AD 648 – 765 | - | 3 | 6 – 7 | 3 | - Shell-stamped pottery: spouts and some lugs, jars, 6mm thick, some incised decoration. - Sheep: "A small number of sheep (from levels BSWS and OMSW) and grey duiker bones are represented but as they are from the same size class, it is uncertain which might dominate among the small medium bovids” [121: 75]. - Ochre stained upper grindstone |
| **MGR**  Botswana | **Magarape**  Inland open-air riverside site/farmer  [168, 169] | 1350±80  KN-2641 | AD 648 – 841 | - | 3 | 6 – 7 | 7i | - Mzonjani type pottery [169] or Kalundu type pottery [168] - Fe fragments, Fe slag - Carbonized sorghum - Pits, burnt post-hole, 65 burnt hut floors (possibly arranged in circular pattern), stone floors, cairn.   LSA micro-lithics present at site. |
| **MES**  Namibia | **Messum 1**  Inland LSA rock shelter  [3, 18, 170] | 1370±50  Pta-2681 | AD 652 – 765 | - | 2 | 6 – 7 | 5 | - No description of pottery - Fe artefact |
| **BYN**  South Africa | **Bynekranskop**  Coastal LSA cave site/pastoralist  [15, 171] | 1370±60  OxA-3863  **sheep AMS** | AD 652 – 765 | - | 1 | 6 – 7 | 3 | - Mostly undecorated pottery, spouts present.   Total livestock at site:   - 11 (M) sheep |
| **WOS**  South Africa | **Wosi**  Inland open-air riverside site/farmer  [172, 173, 174] | 1460±50  Pta-4100  1430±60  Pta-4104 | AD 592 – 662  AD 595 – 757 | AD 652 | 2 | 6 | 9 | - Msuluzi type pottery: everted and curved necked pots with bands of decoration on neck (alternating cross-hatching, herringbone designs), body decoration is present but less common than Mzonjani ware, some burnish with red or black, bosses present, bowls are sub-carinated and thick, mostly undecorated. - Little evidence of Fe working - 20 (M) sheep, 2 (M) cattle, 2 (M) goat, 45 (M) sheep/goat - Pits, - Upper grindstones, lower grindstones |
| **MSC**  South Africa | **Msuluzi Confluence**  Inland open-air riverside site/farmer  [174, 175] | 1370±30  Pta-2193  1310±40  Pta-2195 | AD 654 – 763  AD 680 – 835 |  | 2 | 6 – 7 | 9i | - Msuluzi type pottery: everted and curved necked pots with bands of decoration on neck (alternating cross-hatching, herringbone designs), body decoration is present but less common than Mzonjani ware, some burnish with red or black, bosses present, bowls are sub-carinated and thick, mostly undecorated. - Fe slag, tuyere, Fe ore - 4 (M) sheep, 2 (M) cattle, 4 (M) sheep/goat - Pits, - Human burial with pot - Upper grindstone, lower grindstone (U-shaped) - Clay ‘horn’/cattle figurines (?) |
| **BIS**  Botswana | **Bisoli**  Inland open-air site/farmer  [52, 90, 168] | 1340±60  Wits-1099  1240±80  I-12708 | AD 657 – 830  AD 692 – 966 | - | 2 | 7 | 3 | - Bisoli pottery: rolled rims, parallel broad lines on neck and decoration on shoulder (origins with Bambata type pottery) and/or possibly Ziwa/Gokomere type pottery. - Domestic animals are present but no count provided nor type of domesticate. |
| **MPM**  South Africa | **Mpame**  Coastal open-air site/farmer  [176] | 1340±60  Pta-2019  1230±40  Pta-2045 | AD 657 – 830  AD 773 – 892 | - | 2 | 7– 8 | 3 | - Mzuluzi type pottery - Cattle (?) (not analysed) |
| **KBE**  South Africa | **Kasteelberg E**  Coastal boulder site/hunter-herder/pastoralist  [48] | 1360±50  Pta-7455 | AD 659 – 765 | - | 2 | 7 | 1 | - Shell-stamped pottery: spouts, jars, 6mm thick, some incised decoration. |
| **WEL**  South Africa | **Welgeluk**  Inland LSA rock shelter/hunter-gatherer  [59] | 1360±50  Pta-3960 | AD 659 – 765 | - | 3 | 7 | 1 | - Albany ware: well-baked, thin-walled, globular shaped pots with shallow grooved or impressed bands, red burnish occurs on some pots. |
| **TMB**  Malawi | **Tambala**  Open-air site/farmer  [177] | 1360±50  Pta-927 | AD 659 – 765 | - | 3 | 7 | 7i? | - Nkope pottery - Fe arrowheads, Fe razor, Cu ring, Cu anklet, Fe slag - Daga - Basket grain-storage bin(?) |
| **MAG**  South Africa | **Magogo**  Inland open-air riverside site/farmer  [156, 157] | 1360±50  Pta-2874  1320±50  Pta-3716 | AD 659 – 765  AD 675 – 839 |  | 2 | 7 | 8i | - Msuluzi type pottery - Fe beads and blade, Fe slag, tuyere, furnace walls (?) - 2(M) sheep, 13 (M) cattle, 28 (M) sheep/goat - Bulrush millet (eleusine corocana), melon, - Pits, daga (no structure identified besides possible furnace) - Upper grindstone, lower grindstone - Clay ‘horn’ figurines, Clay human figurines with decoration or scarification. |
| **JKKA**  South Africa | **Jakkalsberg A**  Inland open-air site/pastoralist  [13, 165, 166, 167] | 1330±60  Pta-5958  1300±25  Pta-6100 | AD 663 – 838  AD 684 - 836 | - | 2 | 7 | 3 | - Majority of pottery is undecorated, red colour sherds, lug present   Total livestock at site:   - 29 (M) sheep |
| **DK1**  South Africa | **Die Kelders**  Coastal LSA rock shelter/pastoralist  [29] | 1325±60  OxA-3860  **sheep AMS**  1290±60  OxA-3961  **sheep AMS**  1465±100  Gx-1685 | AD 670 – 840  AD 685 – 860  AD 520 -765 |  | 1 | 7 | 3 | - Thin-walled pottery with quartz inclusion, globular or bag-shaped, undecorated, black with red-slip and shell-stamped pottery as well as mostly undecorated pottery with pointed base, spout and bosses, 5.9mm thick. - 2 (M) sheep, 2 (M) cattle (?) (not associated with radiocarbon determination). |
| **SBC**  South Africa | **Steenberg’s Cove**  Coastal boulder site/hunter-gatherer  [48] | 1350±50  Pta-4010  1280±50  Pta-4012  marine shell (corrected) | AD 670 – 766  AD 691 – 876 | - | 3 | 7 | 3 | - Shell-stamped pottery with spouts and some lugs, mostly jars, 6mm thick, some incised decoration. One sherd with two horizontal lines. - 2 (M) sheep |
| **OBP**  South Africa | **Olieboomspoort**  Inland LSA rock shelter/hunter-gatherer  [32] | 1330±50  Pta-7418  1310±40  Pta-7780 | AD 671 – 831  AD 680 – 835 | AD 754 | 2/3 | 7 | 3 | - Bambata A pottery: thin-walled, jars, spout and bosses, grey to black with red slip, comb-stamped and incision, ripple rims. Happy Rest, Eiland, Broedhurst and Icon/Moloko pottery were found in the same stratigraphic levels as Bambata A pottery at different frequencies. - Sheep/goat are present but only in more recent occupation levels. |
| **LIK**  Lesotho | **Likoaeng**  Inland LSA rock shelter/hunter-herder  [66, 67, 178] | 1285±40  GrA-23237  **sheep AMS** | AD 675 – 767 | - | 1 | 7 | 2 | - 1 (M) sheep, 1 (M) sheep/goat |
| **PLT**  South Africa | **Plaston**  Inland open-air riverside site/farmer  [179] | 1315±50  Pta-1635 | AD 677 – 965 | - | 3 | 7 – 8 | 1 | - Mzonjani type pottery - Lower grindstone (grooved) |
| **BLNM**  South Africa | **Balerno Main**  Inland LSA rockshelter/hunter-gatherer  [180] | 1340±40  Pta-8603 | AD 678 – 765 | - | 3 | 7 | 1 | - Bambata A pottery: thin-walled, jars, spout and bosses, grey to black with red slip, comb-stamped and incision, ripple rims and Mzonjani pottery and Happy Rest pottery present. |
| **CHO**  Zambia/Malawi Border | **Chowo River**  Inland open-air river site/farmer  [96] | 1340±40  Beta-182952 | AD 678 – 765 | - | 2 | 7 | 6i | - Chifumbaze Complex type pottery: simple stamped impressions in horizontal bands, thickened rounded rims. - Fe rusted clumps - Daga |
| **KBB**  South Africa | **Kasteelberg B**  Coastal boulder site/hunter-herder/Pastoralist  [36, 44, 45, 46, 47, 48] | 1310±50  Pta-4373  1300±60  Pta-3995  1220±45  Pta-3998  1200±45  Pta-3994 | AD 679 – 841  AD 682 – 857  AD 774 – 960  AD 790 – 974 | - | 2 |  | 3 | - Shell-stamped pottery with spouts and some lugs, jars, 6mm thick, some incised decoration (Cape coastal pottery).   Total livestock at site:   - 138 (M) sheep, 7 (M) cattle, - Grooved (boat-shaped) lower grindstones |
| **CHU**  Zambia | **Chundu**  Inland open-air site/farmer  [181, 182] | 1290±100  N-1138  1160±160  N-1139 | AD 680 – 881  AD 688 – 1044 | - | 2 | 7 – 8 | 4 | - Kumadzulo and Kamangoza type pottery - Fe hoes, Fe arrowheads, Fe axe, Cu bangles - 2 (M) cattle - Human burial with livestock remains (2 cattle skulls) - Clay cattle figurine (?) |
| **ORU**  Namibia | **Orunwanje 95/1**  Inland LSA rock shelter/Herding  [22, 23] | 1306±36  UtC-5946  1280±28  KN-5004  1255±40  UtC-5948  1252±36  UtC-5945 | AD 681 – 838  AD 766 – 895  AD 773 – 879  AD 773 – 880 | - | 2 | 7 – 8 | 3 | - Pottery with grooved horizontal and diagonal lines, incised lines, occasional cross-hatching, oblique parallel lines, black to grey to brown in colour, 6.3mm wall-thickness, fine to medium sized quartz inclusions, spout present. - Sheep, goat |
| **BLY**  South Africa | **Blydefontein**  Inland large rock shelter/hunter- herder  [62, 63, 64, 65, 66, 67, 68] | 1305±31  SMU-1850 (HG)  1255±109  SMU-1925 (CPB) | AD 682 – 837  AD 683 – 960 | - | 2 | 7 – 8 | 1 | - (1) Fibre tempered pottery: described as grass tempered plain ware mostly shaped into bowls or bag-shaped pots and undecorated. (2) Undecorated thin-walled ware described as silt tempered, coil-built pottery with grass and silt temper. (3) Khoe pottery occurs in more recent layers. |
| **LEB**  South Africa | **Lebalelo**  Inland open-air site/farmer  [183] | 1300±50  Pta-8772 | AD 682 – 857 | - | 3 | 7 | 3i | - Mzonjani/Broederstroom pottery - 1 (M) sheep/goat - Pits lined with dung, daga |
| **LPK**  Zimbabwe | **Leopard’s Kopje**  Inland open-air site/farmer  [52, 184, 185, 186] | 1280±90  SR-225  1250±110  SR-55  1130±95  I-4862 | AD 682 – 885  AD 684 – 964  AD 780 – 1037 | - | 2 | 7 – 8 | 8ii? | - Zhizo pottery characterized with the appearance of beakers, graphite burnish on bowls (some red haematite used on vessels), similar to Gokomere pottery. - Cu bangles, Fe slag, - 79 (M) cattle, 162 (M) sheep/goat - Cow peas (although the relationship to radiocarbon dates and early occupation is tenuous due to disturbance at the site) - Pits, daga (structures were not identified at the site), **(5f)** sheep/goat byre |
| **TAU**  Botswana | **Taukome**  Inland open-air site/farmer  [40] | 1265±80  I-11407  1240±80  I-11410 | AD 684 – 889  AD 692 – 966 | - | 2 | 7 – 8 | 3ii | - Zhizo/Taukome pottery - Cattle, sheep/goat (Total at site: 149 M) - Livestock byre |
| **RSF**  South Africa | **Roosfontein**  Inland LSA rockshelter/Hunter-gatherer/pastoralist  [187, 188] | 1290±50  Pta-5931 | AD 685 – 858 | - | 2 | 7 – 8 | 1 | - Grit tempered, undecorated pottery: unburnished and buff-brown-grey in colour; 6 -8 mm thick. |
| **MATL**  Botswana | **Matlapaneng**  Inland open-air site/farmer  [76, 90, 189] | 1270±80  1260±60  1190±80  1120±110 | AD 685 – 886  AD 690 – 888  AD 778 – 988  AD 793 – 1136 | - | 2 | 7 – 8 | 8 | - Pottery similar to Kumadzulo and Dambwa in Zambia - Few Fe artefacts - 38 (M) cattle, 42 (M) sheep/goat - Sorghum |
| **NTS**  South Africa | **Ntsitsana**  Inland open-air riverside site/farmer  [190] | 1290±50  Pta-4684  1180 ± 50  Pta-4687  1180 ± 50  Pta-4695 | AD 685 – 858  AD 876 – 989  AD 876 – 989 | - | 2 | 7 – 8 | 9ii | - Mzuluzi and Ndondondwane pottery - Fe slag, tuyere - Pits, daga, burnt hut floors, livestock byre - Upper grindstones - Clay human figurines, clay human figurine with decoration |
| **KAD**  Zimbabwe | **Kadzi**  Inland open-air riverside site/farmer  [78, 153, 154] | 1290±50  Ua-3195 | AD 685 – 858 | - | 2 | 7 – 8 | 8 | - Kadzi pottery - Fe artefacts, Cu artefacts, Fe slag, tuyere - 1 (M) sheep, 1 (M) cattle, c.f. 1 (M) goat, 3 (M) sheep/goat - Finger millet (Eleusine coracana) |
| **NAN**  South Africa | **Nanda**  Inland open-air riverside site/farmer  [191, 192, 193] | 1275±60  Wits-1917 | AD 690 – 880 | - | 3 | 7 – 8 | 9i | - Msuluzi pottery: everted and curved necked pots with bands of decoration on neck (alternating cross-hatching, herringbone designs), body decoration is present but less common than Mzonjani ware, some burnish with red or black, bosses present, bowls are sub-carinated and thick, mostly undecorated. - Fe slag, tuyere, Fe ore, - 16 (M) sheep, 14 (M) cattle, 5 (M) goat, 34 (M) sheep/goat - Pits, daga (no structure identified) - Human burials with pots - Clay human figurines, clay ‘horn’ figurines |
| **NGA**  Zambia | **Nanga**  Inland open-air site/farmer  [161, 162, 194] | 1240±100  Birm-836  1190±100  Birm-835 | AD 690 – 967  AD 773 – 991 | - | 3 | 7 – 8 | 3 | - Two phases of Early farmer ware were recognized at the site - 23 (M) cattle, 3 (M) sheep/goat - Clay human figurines |
| **MWE**  Zimbabwe | **Mwenezi**  Inland hillside midden/farmer  [195] | 1250±75  Ua-11802 | AD 690 – 952 | - | 2 | 7 – 8 | 2 | - 1 (M) sheep, 1 (M) cow, 1 (M) goat, 1 (M) sheep/goat (all from level 3 associated with radiocarbon date) (accessed through one report) |
| **KBC**  South Africa | **Kasteelberg C**  Coastal LSA rock shelter/hunter-herder/pastoralist  [48] | 1270±50  Pta-3785 | AD 692 – 883 | - | 2 | 7 – 8 | 3 | - Shell-stamped pottery with spouts, jars, 6mm thick, some incised decoration. - 4 (M) sheep |
| **SPR**  South Africa | **Spoegrivier**  Coastal LSA cave site/pastoralist  [13, 14, 15] | 1260±50  GrA-9027  **sheep AMS**  1390±50  Pta-4753 | AD 694 – 888  AD 643 – 765 | - | 1 | 7 – 8 | 3 | - Thin-walled pottery with quartz inclusion, horizontal grooves and undecorated. - sheep |
| **AB1**  South Africa | **Atlantic Beach 1**  Coastal shell midden site/herder  [86] | 1825±15  Pta-7792  shell  1800±50  Pta-7792  shell | AD 695 – 798  AD 703 – 729 | - | 2 | 7 | 3i | - Shell-stamped pottery similar to Die Kelders and Kasteelberg: parallel grooved sherd, spout, red slip. - 3 (M) sheep, stone feature (circular possible stone packed hearth) association to dates however are not secure. - Broken lower grindstone with ochre staining |
| **KMG**  Zambia | **Kamangoza**  Inland open-air site/farmer  [196] | 1015±105  N-419  1040±105  N-421  975±105  N-457  940±105  N-423  900±105  N-426  900±105  N-424  870±105  N-425 | AD 987 – 1185  AD 908 – 1176  AD 1023 – 1207  AD 1035 – 1218  AD 1046 – 1271  AD 1046 – 1271  AD 1048 - 1285 | AD 700 – 800 | 3  ‘All of these samples came from the contact zone between the lowest part of the mound deposit and the sterile sand and should have dated the foundation of the midden. In actual fact, these dates are among the latest and obviously influenced by the extreme cultural mixture seen in the artifact distributions.’ [196: 85] | 7 – 8 | 5i | - Kamangoza pottery showing affiliation to Kalundu, Dambwa and Kumadzulo ware. - Fe arrowheads, Fe knife, Fe chisel, Fe razor, Fe bodkin, Fe needle, Cu wire, Cu bangle frag, Cu ring (?) - Pits |
| **ZMF**  Zambia | **Zambezi Farm**  Inland open-air site/farmer  [182] | 1410±130  N-1140 | AD 544 – 856 | AD 700  Noted by the excavator for when the site was most likely occupied. | 3 | 7 | 4i | - Pottery similar to Kalundu, Dambwa and Kumadzulo ware. - Fe hoe, Fe bangle, Fe arrowheads, Fe bodkins, Fe chisel, Cu strip and wire - Cattle (count not provided) - Pits, post-holes with daga (no floors) sub-rectangular in shape. |
| **BOS**  Botswana | **Bosutswe**  Inland hilltop open air site/farmer  [40, 197, 198] | 1400±60 (East)  TX-6991  1370±60 (East)  TX-6994  (Test Pit excavations) | AD 635 – 765  AD 652 – 765 | AD 705 | 2 | 7 | 4ii? | - Zhizo/Taukome pottery - Cu beads and helix - 2 (M) sheep, 10 (M) cattle, 2 (M) goat, 9 (M) sheep/goat. Cattle outnumber or are equal to the number of sheep present during this time period. Hunted game make up 60% of the faunal assemblage. - Central shallow dung deposit (livestock byre) |
| **DIV**  Botswana | **Divuyu**  Inland open-air site/specularite miners/farmer  [189, 199] | 1400±70  Beta-13260  1370±60  Beta-13269  1330±60  Beta-13264  1330±60  Beta-13265  1220±70  Beta-13267  1190±70  Beta-13266 | AD 635 – 766  AD 652 – 765  AD 663 – 838  AD 663 – 838  AD 772 – 967  AD 780 – 987 | AD 723 | 2 | 7 | 9i | - Divuyu pottery related to Spaced Curvilnear Ware with origins from Congo and northern/central Angola and possibly associated with matrilineal groups: most vessels are small, diameters between 10cm and 16 cm, globular jars, straight sided bowls, in turned rim bowls, shouldered neck bowls, hemispherical bowls. The majority of pots have multiple bands of cross-hatch, comb-stamping, punctates or incisions, charcoal tempered. Four bowls with spouts or lugs**.** Similarities are also found with the pottery at Nqoma. - Fe jewelry, Cu jewelry, Fe slag, Fe ore (?)   Total livestock at site:   - 76 (M) sheep, 6 (M) cattle, 10 (M) goat, 62 (M) sheep/goat - Pole-impressed daga - Human burial with no grave goods |
| **OVI**  Namibia | **Ovizorombuku 96/1**  Inland LSA rock shelter site  [23] | 1275±30  KN-5373 | AD 768 – 874 | - | 2 | 7 – 8 | 1 | - Pottery with 5.5mm wall-thickness, quartz grit, well-fired, some burnished sherds (ochre and black), incised horizontal and/or oblique lines, some cross-hatching and one sherd with herringbone-like motif and another with possible finger-nail impressions. |
| **IGI**  Zambia | **Ingombe Ilede**  Inland hilltop site/farmer  [128] | 1120±200  SR-22  1150±100  SR-23 | AD 767 – 1159  AD 778 – 1025 | - | 2 | 7 – 8 | 8i | - Kangila type pottery: straight necked bowls with round bodies and globular necked pots with a band of decoration (mostly incised or comp-stamped), some graphite burnish, lugs and spouts are present but rare. - Fe gongs, Fe hoes (ceremonial and functional), Fe axe, Fe razors, Fe chisels, Fe fish-hooks, Fe rings, Fe bangles and tubes, Cu bangles, Cu crosses (ingot), Cu razor, Cu needles, Cu spindle whorls, Fe slag (not common) - 6 (N) cattle, 30 (N) sheep/goat (total livestock for level one is 122 (N) - Sorghum - Pits, daga (no structure identified) - 31 human burials with grave goods including human burial with dental alteration and human burials with livestock remains. - Upper grindstone, lower grindstone - Clay human figurines with decoration. |
| **DIA**  South Africa | **Diamant**  Inland open-air site/farmer  [52, 200] | 1250±45  Pta-5216  1240±50  Pta-3620 | AD 770 – 885  AD 769 – 948 | - | 2 | 7 – 8 | 3ii | - Diamant type pottery - 24 (N) cattle, 93 (N) sheep/goat - Pits lined with dung, daga, livestock byre |
| **KNO**  Namibia | **Kanono Mulapo**  Dune shell midden site with farmer pottery/Hunter-gatherer  [201] | 1230±50  Pta-8656  **Charcoal extracted from pottery** | AD 770 - 960 | - | 1 | 7 – 8 | 1 | Total at site:   - Kalomo pottery showing similarities to Kalundu and Gundu (Naviundu from Congo (western origin)) is an ancestor to Gundu pottery. Five vessels with comb-stamping on the rims and band of triangular impressions. Some incised motifs are present. The pottery was associated with shell. |
| **CHB**  Mozambique | **Chibuene**  Coastal trading station/farmer  [202, 203, 204] | 1410±75  Ua-16272  1400±85  St-8495  1270±80  St-8494  1235±50  Ua-16270  1225±70  Ua-12267 | AD 601 – 765  AD 601 – 769  AD 685 – 886  AD 770 – 946  AD 770 – 966 | AD 776 | 2 | 7 | 3 | - (1) Matola pottery and (2) Triangular Incised Ware (origins from East African coast) - 1 (N) sheep, 6 (N) cattle, 3 (N) goat, 8 (N) sheep/goat   The Matola classified pottery does not correlate with the radiocarbon dates. |
| **BRD**  South Africa | **Broederstoom**  Inland open-air site/farmer  [135, 136, 137, 129, 52] | 1350±80  KN-2641  1320±100  Fra-88  1335±60  Wits-1475 | AD 648 – 841  AD 667 – 862  AD 660 – 833 | AD 752 | 2 | 7 | 8ii | - Mzonjani/Broederstroom pottery reclassified from a Kalundu (western stream) origin [129]. - Fe artefacts, Cu beads and chain links, Fe slag, tuyere, **F**e working (forges), Fe ore   Total livestock at site:   - 1 (M) cow, 42 (M) sheep/goat - Pits dung lined, post-hole impressed daga, hut floors (no complete residential unit found), grain-bins, cattle byre - Human burial with grave goods, human burial with dental alteration, infant pot burial - Upper grindstones, lower grindstones with lenticular grooves |
| **KBA**  South Africa | **Kasteelberg A**  Coastal boulder site/hunter-herder/Pastoralist  [36, 44, 45, 46, 47, 48] | 1230±40  Pta-5937 | AD 771 – 956 | - | 2 | 7 – 8 | 3 | - Shell-stamped pottery with spouts and some lugs, 6mm wall-thickness, jars (Cape coastal pottery)**.**   Total livestock at site:   - 34 (M) sheep, 2 (M) cattle (mostly in the upper levels) |
| **KKH**  South Africa | **Klein Kliphuis**  Inland LSA rock shelter site/hunter-gatherer  [205, 206] | 1230±50  Pta-4672 | AD 771 – 954 | - | 3 | 7 – 8 | 3 | - Pottery with fine quartz temper, black to grey in colour, some ochre burnish (largely undiagnostic) - 1 (M) sheep - Upper grindstone |
| **CHD**  Zambia | **Chondwe**  Inland open-air site/farmer  [115, 128, 207] | 1135±130  GX-1009  1150±145  N-997 | AD 773 – 1127  AD 767 – 1130 | - | 2 | 7 – 8 | 6i | - Chondwe Group: the lower levels yielded potsherds of Early Iron Age type characterized by thick fabric, gritty or sandy paste, and thickened or undifferentiated rims. The most frequent decorative motifs are lines of false-relief chevron stamping and designs composed of segmental blocks of comb-stamping delineated by broad grooves. A limited number of incised designs also occur. Undecorated vessels are rare. Two sherds with applied bosses. - Fe bead, Cu strips, Fe slag, tuyere - Daga (no pole-impressions identified or hut floors) - Lower grindstone with lenticular grooves (surface find).   Chipped quartz artefacts of LSA type recovered. |
| **KUL**  South Africa  (Most southerly spread of farmers) | **Kulubele**  Inland open-air riverside site/farmer  [208] | 1250±40  Pta-5865 | AD 773 – 881 | - | 3 | 7 – 8 | 1i | - Mzuluzi pottery and/or Ndondondwane pottery - Pits, daga (no structured identified) |
| **SCO**  South Africa | **Scott’s Cave**  Coastal small rock shelter/hunters-with-sheep  [209, 210, 211] | 1190±100  SR-82 | AD 773 – 991 | - | 3 | 7 – 8 | 2 | - 1 (M) sheep |
| **BJP**  South Africa | **Biesje Poort**  Inland open-air site/herder  [74, 94, 109] | 1210±70  Pta-4772 | AD 774 – 970 | - | 2 | 7 – 8 | 1 | - Pottery assemblage found with two spout fragments, two decorated with incised lines and row of punctates. Burnished sherds, some grass tempering, relatively thin-walled (5.3mm). |
| **MGR**  Botswana | **Magagarape**  Inland open-air site/farmer  [168] | 1220±50  Beta-30641 | AD 774 – 961 | - | 3 | 7 – 8 | 7i | - Baratani pottery - Fe artefacts, Fe slag - Sorghum - Pits, post-holes, 65 burnt daga features (hut floors) (no definite layout could be discerned but possibly circular), stone floors and stone cairn.   LSA microlithics and ostrich eggshell beads were recovered from the site. |
| **NDD**  South Africa | **Ndondondwane**  Inland open-air riverside site/farmer  [174, 212, 213, 214] | 1220±50  Pta-238 | AD 774 – 819 | - | 2 | 7 | 8ii | - Ndondondwane pottery with similarities to Msuluzi pottery: narrow multiple bands of decoration on neck, less decoration on body and shoulder of pot, cross-hatching more common. - Fe rolled strip, Cu bead - 459 (N) sheep, 960 (N) cattle, 47 (N) goat, 1458 (N) sheep/goat - Bulrush millet - Burnt daga floors (one possibly beehive shaped and possibly men’s hut due to its location near iron working area and byre), livestock byre, furnace remains - Infant burial in pot - Upper grindstone, lower grindstone - Clay sculpted heads |
| **KANG**  Zambia | **Kangonga**  Inland open-air site/farmer  [207, 215] | 1185±85  GX-1327 | AD 776 – 990 | - | 3 | 7– 8 | 6i | - Chondwe Group: the lower levels yielded potsherds of Early Iron Age type characterized by thick fabric, gritty or sandy paste, and thickened or undifferentiated rims. The most frequent decorative motifs are lines of false-relief chevron stamping and designs composed of segmental blocks of comb-stamping delineated by broad grooves. A limited number of incised designs also occur. Undecorated vessels are rare. - Fe slag - Pole-impressed daga |
| **WON**  South Africa | **Wonderwerk**  Inland LSA Cave site/hunter-herders/pastoralist  [87] | After 1210±50  Pta-2546 | After AD 777 - 967 | - | 3 | 7 – 8 | 3? | - Undecorated pottery, 5.6mm thick-walled, black. - sheep/goat (?) |
| **SK17**  South Africa | **SK17**  Inland open-air site/farmer  [138, 139, 52] | 1210±50  Pta-3507 | AD 777 – 967 | - | 3 | 7 – 8 | 3 | - Garonga pottery [52] or Kalundu (Ndondondwane/Lydenburg) pottery [138,139] - 1 (M) cattle, 5 (M) sheep/goat |
| **QOG**  Botswana | **Qogana**  Inland open-air site/farmer  [40, 90] | 1190±80 | AD 778 – 988 | - | 3 | 7 – 8 | 1i | - Pottery similar to Matlapaneng - Burnt reed-impressed clay hut |
| **WOS**  South Africa | **Wosi**  Inland open-air riverside site/farmer  [172, 173, 174] | 1290±50  Pta-4094  1270±60  Pta-4095 | AD 685 – 858  AD 690 – 883 | AD 779 | 2 | 7 | 9i | - Ndondondwane pottery with similarities to Msuluzi pottery: narrow multiple bands of decoration on neck, less decoration on body and shoulder of pot, cross-hatching more common. - Fe artefacts, Fe slag - 80 (M) sheep, 36 (M) cattle, 6 (M) goat - Pits, pole-impressed daga, hut floor - Human burial without grave goods - Upper grindstones, lower grindstones - Hollow clay sculpted heads (therianthropic ‘horns’) |
| **KLD**  Zambia | **Kalundo Mound**  Inland open-air mound site/farmer  [17] | 1160±90  SR-41 | AD 780 – 1020 | - | 2 | 7 – 8 | 8i? | - Kalundo pottery: multiple band decoration on neck, shouldered vessels, thin-walled, globular shaped pots, bowls, thick-walled, spouts, lugs and bosses are present (but not numerous), burnishing present (mostly graphite around necked pots and bowls), comb-stamping common feature of decoration, increase in bag-shaped pots with increase in livestock at site. - Fe strip, Cu bracelet, Fe slag (small amount), Fe tuyere (1 piece), bellow nozzles   Total livestock at site:   - 3(N) cattle, sheep/goat (number not provided) - Pits, daga (no identifiable floor), grain-bin (?) - Human burials with no grave goods - Clay humped cattle figurines |
| **KLF**  Zambia | **Kalambo Falls (Site A)**  Inland open-air hill site/farmer  [112, 113] | 1080±180  L-395B | AD 780 – 1184 | - | 2 | 7 – 8 | 6i | - Kalambo pottery: channel decorated with the majority of vessels from Site A being undecorated body sherds. Some thin-walled dark grey sherds were also present. Three different types of ceramics identified**.** - Fe fragments, Cu bracelet or anklet, Cu conical object, Fe slag - Pit, daga (no identified structure) - Upper grindstone, lower grindstone |
| **DC19**  Swaziland | **2531DC19**  Inland open-air site. Pottery from survey and test pit excavation/farmer  [216] | AD780±40  X-186  **Pottery OSL** | AD780±40 | - | 1 | 7 | 1 | - Mzonjani pottery: pots with straight or everted necks, single horizontal grooved decoration (impressions or incision) below lip, discontinuous body decoration on pot, ‘wavy’, zig-zag, chevron designs, bowls: thick, sub-carinated with horizontal grooved decoration below carination. |
| **DAM**  Zambia | **Dambwa**  Inland open-air site/farmer  [128] | 1350±100  SR-106  1330±110  SR-62  1290±120  SR-119  1200±95  SR-97  1170±90  SR-98 | AD 646 – 857  AD 654 – 862  AD 665 – 947  AD 773 – 987  AD 779 – 1015 | AD 819 | 2 | 7 | 6i | - Dambwa pottery: comb-stamping, multiple bands around neck of carinated pot, graphite burnish, thin- walled. Carinated bowls also occur. Larger thick-walled vessels with decoration on rim and body. - Fe arrowheads, Cu wire, Fe slag - Pits, post-hole impressed daga - Human burials without grave goods. |
| **FIB**  Zambia | **Fibobe 2**  Inland open-air site/farmer  [61] | 1450±110  NZ-4999  1200±65  NZ-5000  1175±85  NZ-5004 | AD 550 – 837  AD779 – 978  AD777 – 995 | AD 819 | 2 | 7 | 5i | - Pottery with bangle impressed pendant loops, comb-stamped diagonal bands. - Fe hoe shaft fragment, Fe razor (no information of where at the site the material derived). - Cu bangle fragment (no information of where at the site the material derived) - Pit, daga (units B, F and J), possible posts (unit F). |
| **NQM**  Botswana | **Nqoma**  Inland open-air site/famer  [76, 199, 217] | 1290±60  Beta-13257  1220±70  Beta-13256 | AD 685 – 860  AD 772 – 967 | AD 820 | 2 | 7 | 4i | - Divuyu and Xaro type pottery: small, diameters between 10cm and 16 cm, globular jars, straight sided bowls, in turned rim bowls, shouldered neck bowls, hemispherical bowls. The majority of pots have multiple bands of cross-hatch, comb-stamping, punctates or incisions. - Fe jewelry and few tools, Cu jewelry - 73 (N) sheep, 137 (N) cattle (some of which are of a humped-back variety), 2 (N) goat, 19 (N) sheep/goat (central area/society area and includes upper levels with Nqoma type pottery). - Daga - Infant burial with no grave goods, human burial with Fe and Cu jewelry and burial (Bantu-language speaker) with Nqoma style vessel, human burial (Khoisan) with stone cairn. - The site contains a pre-AD700 occupation dating possibly to AD 200 – 600 and is described by the presence of thin-walled pottery similar to other sites in the area – Depression Shelter, Ancestors’ Cave and Rhino cave 9 [217]. |
| **KGG**  South Africa | **KwaGandaganda**  Inland open-air riverside site/farmer  [158, 159, 160] | 1260±60  Wits-1937  1245±60  Wits-1919 | AD 690 – 888  AD 766 – 957 | AD 825 | 2 | 7 | 9ii | - Ndondondwane pottery with similarities to Msuluzi pottery: narrow multiple bands of decoration on neck, less decoration on body and shoulder of pot, cross-hatching more common. - Fe beads and rods, Cu beads and earring (?), Fe slag - 62 (N) sheep, 599 (N) cattle, 22 (N) goat, 1225 (N) sheep/goat - Pits, pole-impressed daga, grain-bins, livestock byres - Upper grindstone, lower grindstone - Clay human figurines (decorated or scarification), small ceramic head |
| **DOM**  Botswana | **Dombashaba**  Inland hilltop site/farmer  [6, 52] | 1150±80  I-13746 | AD 859 – 1024 | - | 3 | 8 | 1 | - Bisoli pottery: rolled rims, parallel broad lines on neck and decoration on shoulder (origins with Bambata pottery). |
| **MAG**  South Africa | **Magogo**  Inland open-air riverside site/farmer  [156, 157] | 1190±50  Pta-2875 | AD 861 – 986 | - | 2 | 8 | 8i | - Ndondondwane pottery - 2 (M) sheep, 28 (M) cattle, 13 (M) sheep/goat - Sorghum (?), bulrush millet, finger millet - Pits |
| **NDD**  South Africa | **Ndondondwane**  Inland open-air riverside site/farmer  [212; 213 214,174] | 1190±50  Pta-2389 | AD 861 – 986 | - | 2 | 8 | 8ii | - Ndondondwane pottery with similarities to Msuluzi pottery: narrow multiple bands of decoration on neck, less decoration on body and shoulder of pot, cross-hatching more common. - Fe rolled strip, Cu bead - 459 (N) sheep, 960 (N) cattle, 47 (N) goat, 1458 (N) sheep/goat - Bulrush millet - Burnt daga floors (one possibly beehive shaped and possibly men’s hut due to its location near iron working area and byre), livestock byre, furnace remains - Infant burial in pot - Upper grindstone, lower grindstone - Clay sculpted heads |
| **MAM**  South Africa | **Mamba**  Inland open-air riverside site/farmer  [163, 164] | 1170±60  Pta-4164 | AD 862 – 994 | - | 2 | 8 | 9ii | - Ndondondwane pottery with similarities to Msuluzi pottery: narrow multiple bands of decoration on neck, less decoration on body and shoulder of pot, cross-hatching more common. - Fe slag, tuyere, Fe ore, furnace wall - 10 (M) sheep, 37 (M) cattle, 1 (M) goat, 25 (M) sheep/goat - Pit, pole-impressed daga, livestock byre - Lower grindstone with elliptical grooves - Sculptured clay heads |
| **KZ1**  Zambia | **Kazindu**  Inland open-air riverside site/farmer  [161] | 1150±75  N-2315 | AD 863 – 1023 | - | 3 | 8 | 6 | - Early farmer pottery - Fe slag |
| **EBC**  South Africa | **Elands Bay Cave**  Coastal LSA cave site/hunter-gatherer  [119, 120, 121] | 1120±85  Gak-4335 | AD 876 – 1127 |  | 2 | 8 | 3 | - KBB/KBA style, including beveled lips, shell-edge-stamped decorations and spouts. - 1 (M) sheep |
| **ORU**  Namibia | **Orunwanje 95/1**  Inland LSA rock shelter/Herding  [22, 23] | 1194±32  UtC-5943  1178±32  UtC-5586  1172±32  UtC-5939  1120±55  KN-5299 | AD 879 – 970  AD 888 – 970  AD 892 – 970  AD 895 - 1021 | - | 2 | 8 | 1 | - Pottery with grooved horizontal and diagonal lines, incised lines, occasional cross-hatching, oblique parallel lines, black to grey to brown in colour, 6.3mm wall-thickness, fine to medium sized quartz inclusions, spout present. |
| **NQM**  Botswana | **Nqoma**  Inland open-air site/farmer  [77, 199, 217] | 1100±80  Iso-11411  1000±60  Beta-13260 | AD 880 – 1127  AD 1028 – 1150 | - | 2 | 8 | 8i | - Nqoma type pottery: variations of pendant and interlocking triangles filled with incised or comb-stamped parallel bands or cross-hatched lines. Vessels have red ochre burnish. - Fe jewelry (few tools), Cu jewelry, tuyere, - 19 (N) sheep, 62 (N) cattle (some of which are of a humped-back variety), 6 (N) sheep/goat (faunal count does not include upper Nqoma levels from central/society area). - Sorghum - Burnt daga wall fragments with pole-impressions (shape could not be determined). |
| **BOS**  **Botswana** | **Bosutswe**  Inland hilltop open-air site/farmer  [40, 197, 198] | 1230±60 (Central)  TX-6997  1200±60 (West)  TX-6992  1170±80 (Central)  TX-6982 | AD 770 – 960  AD 779 – 975  AD 785 – 1014 | AD 881 | 2 | 8 | 4ii | - Toutswe type pottery - Cu beads and helix - 5 (M) sheep, 52 (M) cattle, 2 (M) goat, 39 (M) sheep/goat [197]. Cattle outnumber sheep during this time period [198]. - Thatched pole and daga structures, livestock byres present |
| **CAE**  Botswana | **Cae Cae (Xai-Xai)**  Inland open-air pan site with farmer and pastoralist items  [76, 77] | 1150±60 | AD 886 - 1013 | - | 3 | 8 | 9 | - Pottery - Metal use - 1 (N) cow |
| **SHO**  South Africa | **Shongweni South**  Inland Cave site with a farmer occupation  [218] | 1170±50  Pta-1059 | AD 886 – 987 | - | 3 | 8 | 10 | - Early farmer pottery - Sorghum - Dung floor present |
| **NBC**  South Africa | **Nelson Bay Cave**  Coastal cave site/hunter-gatherer  [15, 35, 219] | 1100±80  OxA-873  **sheep AMS** | AD 888 – 1127 | - | 1 | 8 | 3 | - Undecorated pottery - 3 (M) sheep |
| **KSBD**  Namibia | **!Khuiseb Delta (Ward’s Midden)**  Shell midden site/hunter- herder/pastoralist  [57, 107] | 1170±50  KN-3598 | AD 890 – 981 | - | 3 | 8 | 1 | - The pottery comprised small globular vessels, highly burnished and bearing in some cases, elaborate decoration on the neck and rim. |
| **GNE**  South Africa | **Glen Elliot**  Inland rock shelter/hunter-herder  [220] | 1120±70  Pta-3402/75  **Fibre from pottery** | AD 890 – 1025 | - | 3 | 8 | 1 | - Fibre tempered pottery (Class A) with some (Class B) grit temper pottery. |
| **Le6**  South Africa | **Le6**  Inland open-air site/farmer  [138, 139, 52] | 1160±50  Pta-3491 | AD 891 – 1020 | - | 3 | 8 | 3 | - Malapati Facies [52] or Gokomere/Ziwa [139]. - 14 (M) cattle, 1 (M) sheep/goat |
| **BKK**  South Africa | **Blinklipkop**  Inland specularite mining site/hunter-herder  [53, 87] | 1160±50  Pta-2840  1150±40  Pta-2835 | AD 891 – 987  AD 894 – 988 | - | 3 | 8 | 3 | - Ripple Ware: 6.3mm wall-thickness and undecorated pottery - 1 (M) sheep/goat |
| **SCH**  South Africa | **Schroda**  Inland open-air site/farmer  [127, 221] | 1160±50 Area 5  Pta-1967  1170±20  Pta-7666  1140±45  Pta-7664  1110±50  Pta-1819  1060±20  Pta-7650  1030±45  Pta-7659 | AD 891 – 987  AD 893 – 970  AD 894 – 993  AD 898 – 1024  AD 995 – 1029  AD 1016 – 1145 | - | 2 | 8 | 8i? | - Zhizo pottery: stamped decorated ware, globular pots with everted rim with stamped decoration under the rim and/or neck. - Fe beads (all levels and areas), Fe sweat scrapers, Fe adze, Fe awl (Area 4), Fe bangles (Area 2 and 5) Fe hoe, Fe adze (Area 5), Cu beads, Cu ferrule, Cu spiral, Fe slag (surface), tuyere.   Total livestock at site:   - 201 (M) cattle, 263 sheep/goat. - Possible charred sorghum (Areas 5 and 6) - 2 Pits (Area 2), burnt daga, post-holes, circular/oval hut floor, stone-walling (Area 2)   Total human burials at site:   - 5: human burial without grave goods, human burial with grave goods - Upper grindstones/hammerstones, lower grindstone with elongated hollow (Area 6), complete grindstone with hut remains (Area 2). - Cattle clay figurines, sculpted female figurines, sculpted human figurines with scarification and/or elongated, numerous sculpted clay fragments recovered from the site including birds and giraffe. |
| **KAB**  Zambia | **Kabondo Kumbo**  Inland open-air river site/farmer  [134] | 1240±65  N-1922  1160±85  N-1920  1150±85  N-1919 | AD 766 – 964  AD 785 – 1020  AD 790 – 1024 | AD 892 | 2 | 8 | 6i | - Kamangoza, Dambwa and Kumadzulo type pottery - Fe hoes, Fe arrowheads, Fe spearheads, small Fe axe, Fe knives, Fe bodkins, Fe razors, FE bracelet, Cu bangles, Fe slag, tuyere - Pits (one pit with bottomless pot), pole-impressed daga, hut-floor (sub-rectangular. - Clay cattle figurine (?) |
| **NTKE**  South Africa | **Ntshekane**  Inland open-air riverside site/farmer  [222] | 1150±45  Pta-1058  1100±50  Pta-1057 | AD 893 – 989  AD 899 – 1028 | - | 2 | 8 | 9i | - Ntshekane type pottery: tall necked vessels and mostly open-mouthed bowls. Bowls are generally undecorated. Burnishing occurs (red ochre, graphite and black). Continuous bands of decoration around the neck of vessels with parallel grooves or incision. Sometimes pendent shaped decoration on body of pot. - Fe slag, tuyere, Fe ore (?) - 20 (M) sheep, 14 (M) cattle - Pits, pole-impressed daga, hut floor (dimension could not be identified) - Upper grindstones, lower grindstones (long oval grooves) - Clay cattle figurine |
| **OBP**  South Africa | **Olieboomspoort**  Inland LSA rock shelter/Hunter-gatherer  [32] | 1140±40  Pta-7416 | AD 895 – 992 | - | 2/3 | 8 | 3? | - Bambata A pottery: thin-walled, jars, spout and bosses, grey to black with red slip, comb-stamped and incision, ripple rims. Happy Rest, Eiland, Broedhurst and Icon/Moloko pottery were found in the same stratigraphic levels as Bambata A pottery at different frequencies. - sheep/goat are present but only in more recent occupation levels. |
| **TAF**  Zimbabwe | **Tafuna Hill**  Inland mining site/farmer  [223, 224] | 1070±105  N-1148 | AD 895 – 1150 | - | 3 | 8 | 6i | - Coronation type pottery - Fe razor, Fe blade, Fe slag, tuyere - Pole-impressed daga (no structure identified) |
| **THD**  Zambia | **Thandwe**  Inland LSA rock shelter with farmer pottery  [100] | 1060±110  N-906 | AD 896 – 1155 | - | 3 | 8 | 1 | - Early farmer pottery |
| **LRY**  Zimbabwe | **Lanlory**  Inland open-air site/farmer  [225, 226] | 1130±50  Pta-606 | AD 896 – 1016 | - | 3 | 8 | 6i | - Early farmer pottery similar to Kapwirimbwe in Zambia - Fe bangle, Cu bangle, Fe slag, tuyere - Pole-impressed daga - Human burial with grave good, human burial with dental alteration. |
| **KAP**  Namibia | **Kapako**  Inland open-air surface site/Iron working groups  [99, 227] | 1110±50  Pta-234 | AD 898 – 1024 | - | 3 | 8 | 6i | - Pottery is brittle and 10 mm thick. A large necked bowl is present with a thickened lip. Decoration consists of a broad band of incised horizontal lines, herringbone, cross-hatching or a combination of these elements. - Fe ring and point, Fe slag - Pits |
| **TOT3**  Botswana | **Toteng 3**  Inland open-air site/hunter-herder/pastoralist  [6, 25, 26, 27] | 1130±40  Beta-186671  **sheep AMS** | AD 897 – 1014 | - | 1 | 8 | 2 | - sheep |
| **LE 7**  South Africa | **Le7a**  Inland open-air site/farmer  [138, 139, 52] | 1120±50  Pta-3478 | AD 897 – 1020 | - | 3 | 8 | 3 | - Malapati ware [52] or Eiland/Matola [138, 139] - 12 (M) cattle, 3 (M) sheep/goat |
| **PTD**  South Africa | **Pont Drift**  Inland open-air site/farmer  [52] | 1110±50  Pta-1961 | AD 898 – 1024 | - | 3 | 8 | 3 | - Zhizo pottery characterized with the appearance of beakers, graphite burnish on bowls (some red haematite used on vessels). Similar to Gokomere pottery. - 59 (M) cattle, 119 (M) sheep/goat |
| **KBB**  South Africa | **Kasteelberg B**  Coastal boulder site/hunter-herder/Pastoralist  [36, 44, 45, 46, 47, 48] | 1100±50  Pta-5083  990±35  Pta-4500 | AD 899 – 928  AD 1036 – 1147 | - | 2 | 8 | 3 | - (1) Shell-stamped pottery with spouts with some lugs, 6mm, jars (Cape coastal pottery), (2) undecorated, lugged pottery.   Total livestock at site:   - 138 (M) sheep, 7 (M) cattle - Grooved (boat-shaped) grindstones |
| **DOF** | **Doornfontein**  Inland cave site (ancient working site)/pastoralists  [35, 228] | 1120±40  Pta-186 | AD 900 – 1018 | - | 3 | 8 | 1 | - Doornfontein type pottery: thin-walled with horizontally pierced lugs (some Iron Age pottery found). |
| **NAMB**  Malawi | **Namaso Bay**  Open-air site/farmer  [110] | 1075±75  GX-14813 | AD 900 – 1140 | - | 2 | 8 | 5i | - Namaso pottery: sub-spherical bowls with slightly constricted mouths, open hemispherical bowls both highly decorated with single line-impressions or comb-stamp near rim or towards shoulder in a mostly crisscross (stich) motif**.** - Fe corroded strip, Cu spirally wound wire - Post-hole daga - Upper grindstone, lower grindstone with oval groove mark |
| **KGG**  South Africa | **KwaGandaganda**  Inland open-air riverside site/farmer  [158, 159, 160] | 1080±60  Wits-1920 | AD 900 – 1130 | - | 2 | 8 | 9i | - Ntshekane pottery - Fe slag, tuyere - 18 (N) cattle, 37 (N) sheep/goat - Pits (bottomless pot present in pit), daga, pole-impressed daga, hut floors (roughly encircled around a central area). - Lower grindstones |
| **BES**  Namibia | **Big Elephant Shelter**  Inland LSA rock shelter/hunter-gatherer  [149, 150] | 1080±50  Pta-1558 | AD 901 – 1105 | - | 3 | 8 | 3 | - Undecorated pottery, one decorated with two vertical lines of horizontal nail impressions (similar to Dama or Nama pottery), one spout present. - 4 (M) sheep, 1 (M) c.f. cattle |
| **CHD**  Zambia | **Chondwe**  Inland open-air site/farmer  [115, 207, 229] | 1060±95  GX-1010 | AD 903 – 1150 | - | 2 | 8 | 6i | - Chondwe Group: the lower levels yielded potsherds of Early Iron Age type characterized by thick fabric, gritty or sandy paste, and thickened or undifferentiated rims. The most frequent decorative motifs are lines of false-relief chevron stamping and designs composed of segmental blocks of comb-stamping delineated by broad grooves. A limited number of incised designs also occur. Undecorated vessels are rare. Two sherds with applied bosses. - Fe bead, Cu strips, Fe slag, tuyere - Daga (no pole-impressions identified or hut floors) - Lower grindstone with lenticular grooves (surface find). - Chipped quartz artefacts of LSA type recovered. |
| **KLD**  Zambia | **Kalundo Mound**  Inland open-air mound site/farmer  [17] | 1050±100  SR-66 | AD 904 – 1156 | - | 2 | 8 | 8i? | - Kalundo Tradition pottery: multiple band decoration on neck, shouldered vessels, thin-walled, globular shaped pots; bowls, thick-walled, spouts, lugs and bosses are present (but not numerous), burnishing present (mostly graphite around necked pots and bowls), comb-stamping common feature of decoration, increase in bag-shaped pots with increase in livestock at site. - Fe strip, Cu bracelet, Fe slag (small amount), tuyere (1 piece), bellow nozzles   Total livestock at site:   - 3(N) cattle; sheep/goat (number not provided) - Pits, daga (no identifiable floor), grain-bin (?) - Human burials with no grave goods - Clay humped cattle figurines |
| **LUB**  Zambia | **Lubusi**  Inland open-air site/farmer  [100] | 1070±60  SR-216 | AD 909 – 1139 | - | 3 | 8 | 6 | - Early farmer pottery (similar to some Angolan ceramic assemblages) - Fe working |
| **CHB**  Mozambique | **Chibuene**  Coastal trading station/farmer  [202, 203, 204] | 1180±50  R-1325  1155±85  St-8496  1080±80  St-8495 | AD 876 – 989  AD 891 – 989  AD 896 – 1140 | AD 964 | 2 | 8 | 3 | - (1) Matola type pottery and (2) Triangular Incised Ware (origins from East African coast) - 1 (N) sheep, 6 (N) cattle, 3 (N) goat, 8 (N) sheep/goat - The Matola classified pottery does not correlate with the radiocarbon dates. |
| **CHO**  Zambia | **Chowo River**  Inland open-air site/farmer  [96] | 1010±130  Beta-182955 | AD 974 – 1216 | - | 3 | 8 | 10 | - Early farming pottery - Sorghum |
| **RVS**  South Africa | **Riverside**  Inland open-air site/farmer  [129, 52] | 1070±40  Pta-7670 | AD 981 – 1127 | - | 2 | 8 | 3ii | - Klingbeil type pottery - Cattle present - Pits, livestock byre |
| **OVI**  Namibia | **Ovizorombuku 96/1**  Inland LSA rock shelter  [23] | 1070±35  KN-5184  1042±33  UtC-8103 | AD 986 – 1040  AD 995 – 1131 | - | 2 | 8 | 1 | - Pottery with 5.5mm thick-walls, quartz grit, well fired; some burnished sherds (ochre and black), incised horizontal and/or oblique lines, some cross-hatching and one sherd with herringbone-like motif and another with possible finger-nail impressions. |
| **KMG**  Zambia | **Kamangoza**  Inland open-air site/farmer  [196] | 1015±105  N-419  1040±105  N-421  975±105  N-457  940±105  N-423  900±105  N-426  900±105  N-424  870±105  N-425 | AD 987 – 1185  AD 908 – 1176  AD 1023 – 1207  AD 1035 – 1218  AD 1046 – 1271  AD 1046 – 1271  AD 1048 – 1285 | - | 3 | 8 | 5i | - Kamangoza pottery showing affiliation to Kalundu, Dambwa and Kumadzulo ware. - Fe arrowheads, Fe knife, Fe chisel, Fe razor, Fe bodkin, Fe needle, Cu wire, Cu bangle frag; Cu ring (?) - Pits, traces of hut floors |
| **ZYF**  South Africa | **Zaayfontein**  Inland rock shelter/hunter-herder  [220] | 1050±60  Pta-3393  **Fibre from pottery** | AD 988 – 1145 | - | 3 | 8 | 1 | - Fibre tempered pottery (Class A) with some (Class B) grit temper pottery |
| **DSM**  South Africa | **Drie Susters Main**  Coastal shell midden site/pastoralist  [121] | 1050±60  Pta-5478 | AD 988 – 1145 | - | 3 | 8 | 3 | - Undecoratedd and lugged pottery - 2 (M) cattle, 1 (M) sheep/goat |
| **KAB**  Zambia | **Kabondo Kumbo**  Inland open-air river site/farmer  [134] | 1020±95  N-1917 | AD 990 – 1175 | - | 2 | 8 | 6i | - Shongwe pottery - Fe hoes, Fe slag, tuyere - Post-hole impressed daga, hut floor - Clay cattle figurine, clay human figurine. |
| **KNO**  Namibia | **Kanono Mulapo**  Dune shell midden site with farmer pottery/hunter-gatherer  [201] | 1050±20  Pta-8647  **Charcoal extracted from pottery** | AD 995 – 1035 | - | 1 | 8 | 1 | Total at site:   - Kalomo pottery showing similarities to Kalundu and Gundu (Naviundu from Congo (western origin) is an ancestor to Gundu pottery [117] Total at site: 5 vessels with comb-stamping on the rims and band of triangular impressions. Some incised motifs are present. The pottery was associated with shell. |
| **ZMF**  Zambia | **Zambezi Farm**  Open-air site/farmer  [182] | 910±160  N-1143 | AD 1020 – 1285 | - | 3 | 8 | 4i | - Pottery similar to Kamangoza, Dambwa and Kumadzulo ware. - Fe hoe, Fe bangle, Fe arrowheads, Fe bodkins, Fe chisel, Cu strip and wire - Cattle (count not provided) - Pits, post-holes with daga (no floors but suggested to be sub-rectangular in shape). |
| **AB3**  South Africa | **Atlantic Beach 1**  Coastal shell midden site/herder  [86] | 1490±50  Pta-8797  Shell (not corrected) | AD 1026 – 1122  Shell corrected | - | 3 | 8 | 3 | - Spouted ware - 2 (M) sheep |
| **URU**  Namibia | **Ururu**  Inland LSA rock shelter  [18; 170] | 1000±60  Pta-2664 | AD 1028 – 1150 | - | 3 | 8 | 1 | - Undescribed pottery |
| **GUN**  Zambia | **Gundu**  Inland open-air mound site/farmer  [117] | 965±100  N-428 | AD 1029 – 1204 | - | 3 | 8 | 1i | - Kamangoza type pottery showing affiliation to Kalundu, Dambwa and Kumadzulo ware - Pit, hut floor |
| **ISP**  Zambia | **Isamu Pati**  Inland open-air mound site/farmer  [17] | 910±90  SR-31  890±50  UCLA-176  910±90  SR-80  960±70  UCLA-177  995±61  R874 | AD 1046 – 1265  AD 1154 – 1263  AD 1046 – 1265  AD 1041 – 1181  AD 1030 – 1150 | - | 2 | 8 | 9i | - Kalomo pottery showing similarities to Kalundu and Gundu (Naviundu from Congo (western origin) is an ancestor to Gundu pottery [117] - Fe arrowheads, Fe spearheads, Fe bangle, 1 small Fe ho, Cu bangles, Fe slag, tuyere - 1 (N) cattle, 29 (N) sheep/goat - Sorghum - Pits, pole-impressed daga, hut floor - Human burials with some grave goods (Fe bangle, Cu anklet and pottery), one human burial with dental alteration. - Upper grindstone, lower grindstone - 33 cattle figurines found at the site. |
| **KBA**  South Africa | **Kasteelberg A**  Coastal boulder site/hunter-herder/Pastoralist  [36, 44, 45, 46, 47, 48] | 970±50  Pta-4336 | AD 1043 – 1158 | - | 2 | 8 | 3 | - Undecorated, lugged pottery   Total livestock at site:   - 34 (M) sheep, 2 (M) cattle (mostly in the upper levels) |
| **KFB**  South Africa | **Kreeftebaai**  Coastal shell midden  [44, 120] | 970±50  Pta-5517 | AD 1043 – 1158 |  | 3 | 8 | 1 | - Undecorated, lugged pottery |
| **KN15**  South Africa | **KN2004/015E**  Coastal deflated scatter  [39] | 973±24  OxA-22930 | AD 1045 – 1155 | - | 3 | 8 | 1 | - Pottery with relatively thin-walls (5.76mm), vertical necked vessels |
| **NPL**  Zambia | **Nakapapula Rockshelter**  Inland LSA rock shelter with farmer pottery  [230] | 910±85  GX-767 | AD 1046 – 1265 | - | 3 | 8 | 1 | - Kalambo type pottery |
| **TWK**  Zambia | **Twickenham Road**  Inland open-air site/farmer  [231] | 895±110  GX-662 | AD 1046 – 1274 | - | 3 | 8 | 9i | - Early farmer pottery similar to Kapwirimbe - Fe slag - 1 (M) goat horn - Pit - Lower grindstone |
| **CHI**  Malawi | **Chia Lagoon**  Open-air site/farmer  [232] | 935±50  Pta-2147 | AD 1047 – 1215 | - | 3 | 8 | 1 | - Nkope pottery |
| **BLY**  South Africa | **Blydefontein**  Inland large rockshelter/hunter– herder  [62, 63, 64, 65, 66, 67, 68] | 844±119  SMU-1902 | AD 1048 – 1375 | - | 2 | 8 | 1 | - Khoe pottery |
| **NDN**  Zambia | **Ndonde**  Inland open-air mound site/farmer  [117] | 870±105  N-433 | AD 1048 – 1285 | - | 3 | 8 | 1i | - Kangila type pottery: straight necked bowls with round bodies and globular necked pots with a band of decoration (mostly incised or comb-stamped, some graphite burnish, lugs and spouts are present but rare. - Pits, daga, hut floor - Upper grindstone, lower grindstone |
| **AUS**  Namibia | **Austerlitz**  Inland surface site  [18] | 910±55  KN-I635 | AD 1050 – 1228 | - | 3 | 8 | 1 | - No description of pottery |
| **BJP**  South Africa | **Biesje Poort**  Inland open-air site/herder  [74, 94, 109] | 930±40  Pta-9506 | AD 1051 – 1214 | - | 2 | 8 | 1 | - Pottery assemblage found with two spout fragments, two decorated with incised lines and row of punctates. Burnished sherds, some grass tempering, relatively thin-walled (5.3mm) |

References

1. Pleurdeau D, Imalwa E, Détroit F, Lesur J, Veldman A, et al. “Of Sheep and Men”: Earliest Direct Dated Evidence of Caprine Domestication in Southern Africa at Leopard Cave (Erongo, Namibia). PlosOne. 2012; 7. Available from <https://doi.org/10.1371/journal.pone.0040340>.
2. Kohtamäki M. Transitions: A landscape approach to social and cultural changes in southern Mozambique. Ph.D. Thesis, Uppsala University. 2014.
3. Richter J. Messum 1: A Later Stone Age Pattern of Mobility in the Namib Desert. Cimbebasia B. 1991; 4: 1- 11.
4. Walker NJ. The significance of an early date for pottery and sheep in Zimbabwe. S. Afr. Archaeol. Bull.1983; 38: 88 – 92.
5. Walker NJ. Late Pleistocene and Holocene Hunter-gatherers of the Matopos: An archaeological study of change and continuity in Zimbabwe. Uppsala: Studies in African Archaeology 10; 1995.
6. Huffman TN. The stylistic origin of Bambata and the spread of mixed farming in southern Africa. S. Afr. Hum. 2005 Jan 1;17(1):57-79.
7. Sampson CG, Hart TJ, Wallsmith DL, Blagg JD. The ceramic sequence in the upper Seacow valley: problems and implications. S. Afr. Archaeol. Bull. 1989 Jun 1:3-16.
8. Sadr K, Sampson CG. Through thick and thin: early pottery in southern Africa. J. Afr. Archaeol. 2006; 4: 235 – 252.
9. Mazel AD. Diamond I and Clarke's Shelter: report on excavations in the northern Drakensberg, Natal, South Africa. Annl. Natal Mus. 1984; 26: 25 -70.
10. Mazel AD. Early pottery from the eastern part of southern Africa. S. Afr. Archaeol. Bull. 1992; 47: 3 – 7.
11. Lander F, Russell T. Fat-tailed sheep and thin-walled pots: contextualising rock art and pre-agriculturist pottery within the last 3000 years in KwaZulu-Natal, South Africa. S. Afr. Hum. 2015 Jan 1;27(1):113-63.
12. Leslie-Brooker M. An archaeological study of the Uniondale rockshelter, Albany district, Eastern Cape. Ph.D. Thesis, Stellenbosch University. 1987.
13. Webley LE. The History and Archaeology of Pastoralist and Hunter-Gatherer Settlement in the North-Western Cape, South Africa. Ph.D. Thesis, University of Cape Town. 1992.
14. Webley LE. The Re-Excavation of Spoegrivier Cave on the West Coast of South Africa. Ann. Eastern Cape Mus. 2002, 2: 19 – 49.
15. Sealy J, Yates R. The chronology of the introduction of pastoralism to the Cape, South Africa. Antiquity. 1994 Mar;68(258):58-67.
16. Clark JD, Fagan BM. Charcoal, sands, and channel decorated pottery from Northern Rhodesia. Am. Anthropol. 1965; 67: 354 – 371.
17. Fagan BM. Iron Age cultures in Zambia (Kalomo and Kangila). London: Chatto & Windus; 1967.
18. Wendt WE. Preliminary Report on an Archaeological Research Programme in South West Africa. Cimbebasia. 1972; 2: 2 – 42.
19. Richter J. Messum 1: a Later Stone Age pattern of mobility in the Namib Desert. Cimbebasia. 1984;4 (1-7):1-11.
20. Kinahan J. The stratigraphy and lithic assemblages of Falls Rock Shelter, western Damaraland, Namibia. Cimbebasia B. 1984;4(1-7):13-27.
21. Kinahan J. Archaeological evidence of domestic sheep in the Namib Desert during the first millennium AD. J. Afr. Archaeol. 2016 Nov 1;14(1):7-17.
22. Albrecht M, Berke H, Eichhorn B, Frank T, Kuper R, et al. Oruwanje 95/1: a late Holocene stratigraphy in northwestern Namibia. Cimbebasia. 2001; 17: 1 – 22.
23. Vogelsang R, Eichhorn B. Under the Mopane Tree: Holocene Settlement in Northern Namibia. Köln: Heinrich Barth Institut; 2011.
24. Ossenforf G. Two Holocene Later Stone Age stratigraphies from the Sesfontein area, northwestern Namibia. Azania. 2017; 52: 233 – 266. Available from <http://doi.org/10.1080/0067270X.2017.1296626>.
25. Campbell AC. Southern Okavango Integrated Water Development Study: Archaeological Survey of Proposed Maun Reservoir. Department of Water Affairs, Botswana. November 1992.
26. Robbins LH, Campbell AC, Murphy ML, Brook GA, Liang F, Skaggs SA, et al. Recent archaeological research at Toteng, Botswana: early domesticated livestock in the Kalahari. J. Afr. Archaeol. 2008; 6: 131 – 149.
27. Robbins LH, Campbell AC, Murphy ML, Brook GA, Srivastava P, et al. The Advent of herding in Southern Africa: early AMS dates on domestic livestock from the Kalahari Desert. Curr. Anthropol. 2005; 46: 671 – 677.
28. Beaumont PB. Border Cave. MA Thesis, University of Cape Town. 1978.
29. Schweitzer FR. Excavations at Die Kelders, Cape Province. Annl. S. Afr. Mus. 1979; 78: 101 – 232.
30. Smith AB, Yates R, Miller D, Jacobson L, Evans G. Excavations at Geduld and the appearance of early domestic stock in Namibia. S. Afr. Archaeol. Bull. 1995; 50: 3 – 20.
31. Smith AB, Yates R, Jacobson L. Geduld contra Kinahan. S. Afr. Archaeol. Bull. 1996; 51: 36 – 39.
32. Van der Ryst MM. Seeking shelter: Later Stone Age hunters, gatherers and fishers of Olieboomspoort in the western Waterberg, south of the Limpopo. Ph.D. Thesis, University of the Witwatersrand. 2006.
33. Henshilwood CS. Holocene archaeology of the coastal Garcia State Forest, southern Cape, South Africa. Ph.D. Thesis, University of Cambridge. 1995.
34. Sealy J, Yates, R. Direct radiocarbon dating of early sheep bones: two further results. S. Afr. Archaeol. Bull. 1996; 51:109-110.
35. Klein RG. The prehistory of Stone Age herders in the Cape Province of South Africa. S. Afr. Archaeol. Soc.: Goodwin Series. 1986; 5: 5 – 12.
36. Sadr K. An Ageless View of First Millennium AD Southern African Ceramics. J. Afr. Archaeol. 2008a; 6: 103 – 129. doi 10.3213/1612-1651-10105.
37. Kaplan J. Settlement and subsistence at Renbaan Cave. In: Parkington JE, Hall MJ, editors. Papers in the prehistory of the Western Cape, South Africa. Oxford: British Archaeological Reports International Series; 1987. pp 350-372.
38. Orton JD. Late Holocene archaeology in Namaqualand, South Africa: hunter-gatherers and herders in a semi-arid environment. Ph.D. Thesis, University of Oxford. 2012.
39. Robbins LH, Campbell AC. The Depression Rock Shelter site, Tsodilo Hills. Botswana Notes and Records. 1989 Jan 1;20:1-3.
40. Denbow J. Congo to Kalahari: data and hypotheses about the political economy of the western stream of the Early Iron Age. Afr. Archaeol. Rev. 1990 Dec 1;8(1):139-75.
41. Sampson CG. Chronology and dynamics of Later Stone Age herders in the upper Seacow River valley, South Africa. J. Arid Environ. 2010 Jul 1;74(7):842-8.
42. Avery GA. Systematic investigation of open shell-midden sites along the south-western Cape coast. MA Thesis, University of Cape Town. 1976.
43. Avery G. Discussion on the age and use of tidal fish traps (visvywers). S. Afr. Archaeol. Bull. 1975; 30: 105-113.
44. Sadr K, Smith AB. On ceramic variation in the south-western Cape, South Africa. S. Afr. Archaeol. Bull. 1991 Dec 1:107-14.
45. Sadr K. The first herders at the Cape of Good Hope. Afr. Archaeol. Rev. 1998 Jun 1;15(2):101-32.
46. Sadr K. Invisible herders? The archaeology of Khoekhoe pastoralists. S. Afr. Hum. 2008b Dec 1;20(1):179-203.
47. Sadr K. Radiocarbon dates, stone tools and the origin of herding on the West Coast of South Africa. Frankfurt: Africa Magna Verlag; 2014 Aug 12.
48. Smith AB. Excavations at Kasteelberg and the origins of the Khoekhoen in the Western Cape, South Africa. Oxford: British Archaeological Reports International Series 1537; 2006.
49. Opperman H. The Later Stone Age of the Drakensberg Range and its foothills. Oxford: British Archaeological Reports International Series 339; 1987.
50. Morais J. The early farming communities of southern Mozambique. Sweden: Central Board of National Antiquities; 1988.
51. Silva TC, da Cruz E. First indications of Early Iron Age in southern Mozambique: Matola IV 1/68. In Leakey RE, Ogot, BE, editors. Proceedings of the 8th Pan African Congress of Prehistory and Quaternary Studies, Nairobi 1977. pp. 349-35.
52. Huffman TN. Handbook to the Iron Age: the archaeology of pre-colonial farming societies in southern Africa. KwaZulu-Natal: University of KwaZulu-Natal Press; 2007.
53. Raymond C, Sadr K. Rippled ware at Blinklipkop, Northern Cape. S. Afr. Archaeol. Bull.2010;65(192):196.
54. Senna-Martinez JC, Martins AC, Castelo I, Evangelista BM. Early Iron Age spread into northern Mozambique: revisiting the data from Mozambique Anthropological Missions (1949). Science in the Tropics Proceedings. 2013: 1 – 15.
55. Robinson KR, Sandelowsky B. The Iron Age of northern Malawi: recent work. AZANIA. 1968 Jan 1;3(1):107-46. <https://doi.org/10.1080/00672706809511489>.
56. Sheppard JG, Swart ER. Rhodesian radiocarbon measurements IV. Radiocarbon. 1971; 13: 420 - 431.
57. Kinahan J. Pastoral nomads of the Namib Desert: The people history forgot. Windhoek: Capital Press; 1991.
58. Wadley L. Later Stone Age hunters and gatherers of the southern Transvaal: social and ecological interpretation. Oxford: British Archaeological Reports International Series 25; 1987.
59. Hall SL. Pastoral adaptations and forager reactions in the Eastern Cape. S. Afr. Archeol. Soc.:Goodwin Series. 1986; 5: 42 – 49.
60. Madiquida H. Archaeological and Historical Reconstructions of the Foraging and Farming Communities of the Lower Zambezi: From the mid-Holocene to the second Millennium AD. Uppsala : Department of Archaeology and Ancient History. 2015.
61. Robertson JH. Early Iron Age archaeology in central Zambia. AZANIA. 2000 Jan 1;35(1):147-82.
62. Bousman CB. Holocene paleoecology and Later Stone Age hunter-gatherer adaptations in the South African interior plateau. Ph.D. Thesis, Southern Methodist University.1991.
63. Bousman CB. Coping with risk: Later Stone Age technological strategies at Blydefontein rock shelter, South Africa. J. Anthropol. Archaeol. 2005 Sep 1;24(3):193-226.
64. Horsburgh KA, Moreno-Mayar JV. Molecular identification of sheep at Blydefontein rock shelter, South Africa. S. Afr. Hum. 2015 Jan 1;27(1):65-80.
65. Bousman BC, Mauldin R, Zoppi U, Higham T, Scott L, Brink J. The quest for evidence of domestic stock at Blydefontein rock shelter. S. Afr. Hum. 2016 Jan 1;28(1):39-60.
66. Horsburgh KA, Orton J, Klein RG. Beware the springbok in sheep’s clothing: how secure are the faunal identifications upon which we build our models?. Afr. Archaeol. Rev. 2016 Dec 1;33(4):353-61.
67. Plug I. Reply to Horsburgh et al. 2016: Revisiting the Kalahari debate in the highlands.. Azania. 2017 Oct 20:1-6. Available from <https://doi.org/10.1080/0067270X.2017.1377957>
68. Scott K, Plug I. Osteomorphology and osteometry versus aDNA in taxonomic identification of fragmentary sheep and sheep/goat bones from archaeological deposits: Blydefontein Shelter, Karoo, South Africa. S. Afr.Hum.. 2016 Oct 4;28:61-79.
69. dos Santos Júnior JR, Ervedosa CM. A estação arqueológica de Benfica: Luanda-Angola. Ciencias Biologicas. 1970; 1(1): 31-51.
70. Vogel JC, Morais M. Pretoria Radiocarbon dates I. Radiocarbon. 1971; 13: 378 – 394.
71. Sinclair P, Nydolf NG, Wickman-Nydolf G. Excavations at the University Campus, Maputo, Mozambique, 1984-85. Maputo: Eduardo Mondlane University; 1987.
72. Jerardino AMS. Changing social landscapes of the Western Cape coast of southern Africa over the last 4500 years. Ph.D Thesis, University of Cape Town. 1996.
73. Parsons I. Later Stone Age open-air sites on Bloubos, Northern Cape. Southern African Field Archaeology. 2000; 9: 55 – 67.
74. Parsons I. Later Stone Age socio-economic variability during the last 2000 years in the Northern Cape, South Africa. Ph.D Thesis, University of Cambridge. 2007.
75. Sutton JEG. New radiocarbon dates for eastern and southern Africa. J. Afr. Hist. 1972; 13: 1 – 24.
76. Wilmsen EN. The Antecedents of Contemporary Pastoralism in Western Ngamiland. Botswana Notes and Records. 1989 Jan 1;20:29-39.
77. Yellen JE, Brooks AS. The Late Stone Age archaeology in the/Xai/Xai region: a response to Wilmsen. Botswana Notes and Records. 1990 Jan 1;22:17-9.
78. Katsamudanga S, Pwiti G. Chronology of Early Farming Communities of northern Zimbabwe: a reappraisal. In: Manyanga M, Chirikure S, editors. Archives, objects, places and landscapes: Multidisciplinary approaches to decolonised Zimbabwean pasts. Mankon: Langaa Research & Publishing CIG; 2017. pp. 119 – 136.
79. Maggs T, Ward V. Driel Shelter: rescue at a Late Stone Age site on the Tugela River. Annl. Natal Mus. 1980; 24: 35–70.
80. Klapwijk M. A preliminary report on pottery from the north-eastern Transvaal, South Africa. S. Afr. Archaeol. Bull. 1974; 29: 19 – 23.
81. Klapwijk M, Huffman TN. Excavations at Silver Leaves: a final report. S. Afr. Archaeol. Bull. 1996; 51: 84 – 93.
82. Hall M, Vogel JC. Some recent radiocarbon dates from southern Africa. J. Afr. Hist. 1980 Oct;21(4):431-55.
83. Rodrigues MD. O primeiro sítio com vestígios de utilização do ferro e cerâmica “tradicional” da Early Iron Age localizado em Moçambique–província da Zambézia. Revista Portuguesa de Arqueologia. 2006;9(2):415-49.
84. Mitchell P. Sehonghong: The late Holocene assemblages with pottery. S. Afr. Archaeol. Bull. 1996; 51: 17 – 25.
85. Robertshaw PT. Excavations at Duiker Eiland, Vredenburg District, Cape Province. Annl. Cape Prov. Mus. (Human Sciences). 1979; 1: 1 – 26.
86. Sealy J, Maggs T, Jerardino A, Kaplan J. Excavations at Melkbosstrand: Variability among Herder Sites on Table Bay, South Africa. S. Afr. Archaeol. Bull. 2004; 59: 17 – 28.
87. Humphreys AJB, Thackeray AI. Ghaap and Gariep: Later Stone Age studies in the Northern Cape. South African Archaeological Society Monograph Series 2. 1983.
88. Beaumont PB, Vogel JC. Spatial Patterning of the Ceramic Later Stone Age in the Northern Cape Province, South Africa. In: Hall M, Avery G, Avery DM, Wilson ML, Humphreys AJB, editors. Frontiers: Southern African Archaeology Today. Oxford: British Archaeological Reports International Series 207. 1984.pp. 80 – 95.
89. Mazel AD. Collingham Shelter: the excavation of late Holocene deposits, Natal, South Africa. Natal Mus. J. Hum. 1992b; 4: 1 – 15.
90. Denbow JR, Wilmsen EN. Advent and course of pastoralism in the Kalahari. Science. 1986; 234: 1509 – 1515.
91. Reid A, Sadr K, Hanson-James N. Herding Traditions. In: Lane P, Reid A, Segobye A, editors. Ditswa Mmung: The Archaeology of Botswana. Botswana: Pula Press, University of Botswana, The Botswana Society; 1998. pp. 81 – 100.
92. Derricourt R. Samfya Forest and the chronology of the Iron Age of northern Zambia. Azania. 1976; 11: 153 – 159. Available from <https://doi.org/10.1080/00672707609511235>.
93. Avery G. Open station shell midden sites and associated features from the Pearly Beach Area, South-Western Cape. S. Afr. Archaeol. Bull. 1974; 29: 104 – 114.
94. Beaumont PB, Smith AB, Vogel JC. Before the Eniqua: the archaeology of the frontier zone. In: Smith AB, editor. Einiqualand: Studies of the Orange River Frontier. Rondebosch: UCT Press; 1995. pp. 236 – 265.
95. Evers TM. Recent Iron Age research in the Eastern Transvaal, South Africa. S. Afr. Archaeol. Bull. 1975; 30: 71 – 83.
96. Barham L, Jarman CL. New radiocarbon dates for the Early Iron Age in the Luangwa Valley, eastern Zambia. Azania. 2005; 40: 114 – 121. https://doi.org/10.1080/00672700509480417.
97. Maggs T. Mzonjani and the beginning of the Iron Age in Natal. Annl. Natal Mus. 1980a; 24:71 – 96.
98. Kose E, Richter J. The prehistory of the Kavango people. Sprache und Geshichte in Afrika. 2007; 18: 103 – 129.
99. Kose E. New light on Ironworking groups along the Middle Kavango in northern Namibia. S. Afr. Archaeol. Bull. 2009; 64: 130 – 147.
100. Phillipson DW. The prehistory of Eastern Zambia. Nairobi: British Institute in Eastern Africa; 1976.
101. Robinson KR. A note on the spread of Early Iron Age ceramics in Malawi: tentative suggestions based on recent evidence. S. Afr. Archaeol. Bull. 1976 Dec 1;31(123/124):166-75.
102. Voigt EA. Iron Age herding: archaeological and ethnoarchaeological approaches to pastoral problems. S. Afr. Archaeol. Soc.: Goodwin Series. 1986 Jun 1:13-21.
103. Hall M. Enkwazini, an Iron Age site on the Zululand coast. Annl. Natal Mus. 1980; 24: 94 – 109.
104. Deacon HJ, Deacon J, Brooker M, Wilson ML. The evidence for herding at Boomplaas Cave in the southern Cape, South Africa. S. Afr. Archaeol. Bull. 1978 Jun 1:39-65.
105. Deacon HJ. Excavations at Boomplaas cave, a sequence through the upper Pleistocene and Holocene in South Africa. World Archaeol. 1979 Feb 1; 10(3):241-57.
106. Prinsloo HP. Early Iron Age site at Klein-Afrika near Wylliespoort-Soutpansberg-Mountains, South-Africa. South African Journal of Science. 1974 Jan 1;70(9):271-3.
107. Kinahan J. A new archaeological perspective on nomadic pastoralist expansion in south-western Africa. Azania. 1994 Jan 1;29(1):211-26.
108. Orton J, Mitchell P, Klein R, Steele T, Horsburgh KA. An early date for cattle from Namaqualand, South Africa: implications for the origins of herding in southern Africa. Antiquity. 2013 Mar;87(335):108-20.
109. Badenhorst S, Parsons I. Fauna from five Later Stone Age sites in the Bushmanland region of South Africa. Annl. Ditsong Nat. Mus. Nat. His. 2015 Mar 1;5(1):1-0.
110. Davison S. Namaso: A newly-defined cultural entity of the late first millennium AD, and its place in the Iron Age sequence of southern Malawi. AZANIA. 1991 Jan 1;26(1):13-62. Available from <https://doi.org/10.1080/00672709109511424>.
111. Voight EA. Happy rest: the earlies Iron Age fauna from the Soutpansberg. South African Journal of Science. 1984 May 1;80(5):221.
112. Clark JD. Kalambo Falls prehistoric site: volume 3, the earlier cultures: Middle and Earlier Stone Age. Cambridge: Cambridge University Press; 1969.
113. Fagan BM. Two channel-decorated-pottery sites from Northern Rhodesia. Man. 1964 Jan 1;64:15-6.
114. Whitelaw G, Moon M. The ceramics and distribution of pioneer agriculturists in KwaZulu-Natal. Natal Mus. J. Hum. 1996;8:53-79.
115. Mills EA, Filmer NT. Chondwe Iron Age site, Ndola, Zambia. AZANIA. 1972 Jan 1;7(1):129-45. Available from <https://doi.org/10.1080/00672707209511559>
116. Manyanga M, Shenjere P. The archaeology of the northern Nyanga lowlands and the unfolding farming community sequence in Northeastern Zimbabwe. S. Afr. Archaeol. Bull.. 2012 Dec 1;67(196):244-55.
117. Huffman TN. Iron Age migrations. Johannesburg: Witwatersrand University Press; 1989.
118. Binneman JNF. Archaeological research along the south-eastern Cape coast part 1: open-air shell middens. Southern African Field Archaeology. 2004; 13/14: 49 – 77.
119. Parkington JE. Seasonal mobility in the late Stone Age. African Studies. 1972 Jan 1;31(4):223-44.
120. Parkington J. Coastal settlement between the mouths of the Berg and Olifants Rivers, Cape Province. S. Afr. Archaeol. Bull. 1976 Dec 1;31(123/124):127-40.
121. Smith AB, Sadr K, Gribble J, Yates R. Excavations in the south-western Cape, South Africa, and the archaeological identity of prehistoric hunter-gatherers within the last 2000 years. S. Afr. Archaeol. Bull. 1991 Dec 1:71-91.
122. Robinson KR. An early Iron-Age site from the Chibi district, Southern Rhodesia. S. Afr. Archaeol. Bull. 1961; 16: 75 – 102.
123. Huffman TN. Cattle from Mabveni. S. Afr. Archaeol. Bull. 1975; 30: 23 – 24.
124. Huffman TN. Archaeology and ethnohistory of the African Iron Age. Annl. Rev. Anthropol. 1982; 11: 133 – 150.
125. Sandelowsky BH. Mirabib, an archaeological study in the Namib. Madoqua. 1977 Jan 1;10(4):221-53.
126. Vogel JO, Katanekwa NM. Early Iron Age pottery from western Zambia. AZANIA. 1976 Jan 1;11(1):160-7. Available from <https://doi.org/10.1080/00672707609511236>.
127. Vogel JC. Radiocarbon dating of the Iron Age sequence in the Limpopo Valley. S. Afr. Archaeol. Soc.: Goodwin Series. 2000 Dec 1:51-7.
128. Fagan BM, Phillipson DW, Daniels SGH. Iron Age cultures in Zambia: Dambwa, Ingombe Ilede, and the Tonga. London: Chatto & Windus; 1969.
129. Huffman TN. Presidential address: the antiquity of lobola. S. Afr. Archaeol. Bull. 1998 Dec 1:57-62.
130. Huffman TN. Gokomere pottery from the Tunnel site, Gokomere Mission. S. Afr. Archaeol. Bull. 1976 Jun 1;31(121/122):31-53.
131. Miller SF. The Nachikufan Industries of the Zambian Later Stone Age. Ph.D Thesis: University of California, Berkeley. 1969.
132. Vogel JO. Kumadzulo, an early Iron Age village site in southern Zambia. Oxford: Oxford University Press; 1972.
133. Swan LM. Early iron manufacturing industries in semi-arid, south-eastern Zimbabwe. J. Afr. Archaeol. 2007 Nov 1;5(2):315-38.
134. Vogel JO. Kabondo Kumbo and the Early Iron Age in Victoria Falls region. AZANIA. 1975 Jan 1;10(1):49-75. Available from <https://doi.org/10.1080/00672707509511613>
135. Mason RJ. Background to the Transvaal Iron Age-new discoveries at Olifantspoort and Broederstroom. Journal of the Southern African Institute of Mining and Metallurgy. 1974 Jan 1;74(6):211-6.
136. Huffman TN. Broederstroom and the origins of cattle‐keeping in Southern Africa. Afr. Stud. 1990 Jan 1;49(2):1-2.
137. Huffman TN. Broederstroom and the central cattle pattern. South African Journal of Science. 1993 May 1;89:220.
138. Meyer A. A profile of the iron age in the Kruger national park. In: Hall M, Avery G, Avery DM, Wilson ML, Humphreys AJB, editors. Frontiers: Southern African Archaeology Today. Oxford: British Archaeological Reports International Series 207. 1984. pp. 215-227.
139. Plug I. Aspects of life in the Kruger national park during the early Iron Age. S. Afr. Archaeol. Soc.: Goodwin Series. 1989 Jun 1:62-8.
140. Phillipson DW. The Early Iron Age in Zambia—regional variants and some tentative conclusions. J. Afr. Hist. 1968a Apr;9(2):191-211.
141. Phillipson DW. The Early Iron Age site at Kapwirimbwe, Lusaka. AZANIA. 1968b Jan 1;3(1):87-105. Available from <https://doi.org/10.1080/00672706809511488>
142. Dart RA. The antiquity of mining in southern Africa. South African Journal of Science. 1967; 2: 64.
143. Dart RA, Beaumont PB. Iron Age radiocarbon dates from western Swaziland. S. Afr. Archaeol. Bull. 1969 Jul 1;24(94):71.
144. Stuiver M, van der Merwe NJ. Radiocarbon chronology of the Iron Age in sub-Saharan Africa. Curr. Anthropol. 1968 Feb 1;9(1):54-8.
145. Beaumont PB, Vogel JC. On a new radiocarbon chronology for Africa south of the equator. Afr. Stud. 1972; 31: 65-89.
146. Evers TM, Voigt EA, de Villiers H. Excavations at the Lydenburg Heads site, eastern Transvaal, South Africa. S. Afr. Archaeol. Bull. 1982 Jun 1:16-33.
147. Evers TM. The recognition of groups in the Iron Age of southern Africa. Ph.D Thesis, University of the Witwatersrand. 1988.
148. Whitelaw G. Lydenburg revisited: another look at the Mpumalanga Early Iron Age sequence. S. Afr. Archaeol. Bull. 1996 Dec 1:75-83.
149. Wadley L. Big Elephant Shelter and its role in the Holocene prehistory of central South West Africa. Staatsmuseum; 1979.
150. Wadley L. Radiocarbon dates from Big Elephant Shelter, Erongo Mountains, South West Africa. S. Afr. Archaeol. Bull. 1976 Dec 1:146-.
151. Robinson JR, Rowan J. Holocene paleoenvironmental change in southeastern Africa (Makwe Rockshelter, Zambia): implications for the spread of pastoralism. Quat. Sci. Rev. 2017; 156: 57 – 68. Available from <http://dx.doi.org/10.1016/j.quascirev.2016.11.030>
152. Richter J. Archaeology along the Kavango River/Namibia. Southern African Field Archaeology. 2002;11(12):80-104.
153. Pwiti G. Settlement and subsistence of prehistoric farming communities in the mid-Zambezi valley, Northern Zimbabwe. The S. Afr. Archaeol. Bull. 1996 Jun 1:3-6.
154. Plug I. Early Iron Age buffalo hunters on the Kadzi river, Zimbabwe. Afr. Archaeol. Rev. 1997 Jun 1;14(2):85.
155. Robinson KR. Iron Age occupation north and east of the Mulanje Plateau, Malaŵi. Malawi: Malaŵi Government, Ministry of Local Government, Department of Antiquities; 1977.
156. Maggs T, Ward V. Early Iron Age sites in the Muden area of Natal. Annl. Natal Mus. 1984; 26(1): 105–40.
157. Voigt EA. The faunal remains from Magogo and Mhlopeni: small stock herding in the Early Iron Age of Natal. Annl. Natal Mus. 1984 Dec 1;26(1):141-63.
158. Whitelaw G. KwaGandaganda: settlement patterns in the Natal Early Iron Age. S. Afr. Hum. 1994a Oct 1;6(10):1-64.
159. Whitelaw G. Towards an Early Iron Age worldview: some ideas from KwaZulu-Natal. Azania. 1994b Jan 1;29(1):37-50. Available from <https://doi.org/10.1080/00672709409511660>
160. Miller D, Whitelaw G. Early Iron Age metal working from the site of KwaGandaganda, Natal, South Africa. S. Afr. Archaeol. Bull. 1994 Dec 1:79-89.
161. Katanekwa NM. Some Early Iron Age sites from the Machili valley of south western Zambia. AZANIA. 1978 Jan 1;13(1):135-66.
162. Plug I. Namakala and Nanga: faunal report on two Early Iron Age sites, Zambia. S. Afr. Archaeol. Bull. 1979 Dec 1:123-6.
163. Van Schalkwyk L. Mamba Confluence: a preliminary report on an Early Iron Age industrial centre in the lower Thukela Basin, Natal. S. Afr. Hum.. 1994a Oct 1;6(10):119-52.
164. Voigt EA, Peters J. The faunal assemblage from the Early Iron Age site of Mamba I in the Thukela Valley, Natal. Natal Mus. J. Hum. 1994:145-52.
165. Miller D, Webley L. The metallurgical analysis of artefacts from Jakkalsberg, Richtersveld, Northern Cape. Southern African Field Archaeology. 1994;3:82-93.
166. Brink J, Webley L. Faunal evidence for pastoralist settlement at Jakkalsberg, Richtersveld, Northern Cape Province. Southern African Field Archaeology. 1996; 5(2):70-8.
167. Webley L. Jakkalsberg A and B: the cultural material from two pastoralist sites in the Richtersveld, Northern Cape. Southern African Field Archaeology. 1997;6(1):3-20.
168. Campbell AC, van Waarden C, Holmberg G. Variation in the Early Iron Age of southeastern Botswana. Botswana Notes and Records. 1996 Jan 1:1-22.
169. Huffman TN. A cultural proxy for drought: ritual burning in the Iron Age of southern Africa. J. Archaeol . Sci.. 2009 Apr 30;36(4):991-1005.
170. Vogel JC, Visser E. Pretoria radiocarbon dates II. Radiocarbon. 1981 Jan;23(1):43-80.
171. Schweitzer FR, Wilson ML. Byneskranskop 1: a Late Quaternary living site in the southern Cape Province, South Africa. Annl. S. Afr. Mus. 1982;88.
172. Van Schalkwyk L. Wosi: an early Iron Age village in the lower Thukela Basin, Natal. S. Afr. Hum. 1994b Oct 1;6(10):65-117.
173. Voigt EA, Peters J. The faunal assemblage from Wosi in the Thukela Valley. Natal Mus. J. Hum. 1994b:105-17.
174. Arnold, E.R. A consideration of livestock exploitation during the Early Iron Age in the Thukela Valley, KwaZulu-Natal. In: Badenhorst S, Mitchell, P, Driver JC. Editors. Animals and people: archaeozoological papers in honour of Ina Plug. Oxford: British Archaeological Reports International Series 1349. 2008. pp. 152–68.
175. Maggs T. Msuluzi confluence: a seventh century Early Iron Age site on the Tugela River. Annl. Natal. Mus. 1980b Oct 1;24(1):111-45.
176. Vogel JC, Fuls A. Spatial distribution of radiocarbon dates for the Iron Age in southern Africa. S. Afr. Archaeol.l Bull. 1999 Dec 1:97-101.
177. Robinson KR. Iron Age sites in the Dedza district of Malaŵi. Malawi: Malaŵi Government, Ministry of Local Government, Department of Antiquities; 1973.
178. Mitchell P, Plug I, Bailey G, Woodborne S. Bringing the Kalahari debate to the mountains: late first millennium AD hunter-gatherer/farmer interaction in highland Lesotho. Before Farming. 2008 Jan 1;2008(2):1-22.
179. Evers TM. Plaston Early Iron Age Site, White River District, Eastern Transvaal, South Africa. S. Afr. Archaeol. Bull. 1977 Dec 1;32(126):170-8.
180. Van Doornum B. Sheltered from change: hunter-gatherer occupation of Balerno Main Shelter, Shashe-Limpopo confluence area, South Africa. S. Afr. Hum. 2008 Dec 1;20(2):249-84.
181. Vogel JO. Early Iron Age tools from Chundu Farm, Zambia. Azania. 1970 Jan 1;5(1):173-8. Available from <https://doi.org/10.1080/00672707009511531>
182. Vogel JO. Some Early Iron Age sites in southern and western Zambia. Azania. 1973 Jan 1;8(1):25-54. Available from <https://doi.org/10.1080/00672707309511571>
183. Huffman TN, Schoeman MH. Lebalelo: Early Iron Age pits near Burgersfort, South Africa. S. Afr. Archaeol. Bull. 2011 Dec;66(194):161.
184. Robinson KR. The Leopard's Kopje culture, its position in the Iron Age of Southern Rhodesia. S. Afr. Archaeol. Bull. 1966 Mar 1;21(81):5.
185. Huffman TN. Excavations at Leopard's Kopje Main Kraal: a preliminary report. S. Afr. Archaeol. Bull. 1971 Aug 1:85-9.
186. Voigt EA. Reconstructing Iron Age economies of the northern Transvaal: a preliminary report. S. Afr. Archaeol. Bull.. 1980 Jun 1:39-45.
187. Klatzow S. Roosfontein, a contact site in the eastern Orange Free State. S. Afr. Archaeol. Bull.. 1994 Jun 1:9-15.
188. Thorp CR. An hypothesized frontier between hunter-gatherers and farmers in the Eastern Free State. Ph.D. Thesis, University of the Witwatersrand, 1998.
189. Turner G. Early Iron Age herders in northwestern Botswana: the faunal evidence. Botswana Notes & Records. 1987 Jan 1;19(1):7-23.
190. Prins FE, Granger JE. Early farming communities in northern Transkei: the evidence from Ntsitsana and adjacent areas. Natal Mus. J. Hum. 1993;5(15):3-174.
191. Whitelaw G. Customs and settlement patterns in the first millennium AD: evidence from Nanda, an Early Iron Age site in the Mngeni Valley, Natal. S. Afr. Hum.. 1993 Oct 1;5(10):47-81.
192. Plug I. The faunal remains from Nanda, an Early Iron Age site in Natal. S. Afr. Hum. 1993 Oct 1;5(10):99-107
193. Morris AG. Human remains from the Early Iron Age sites of Nanda and KwaGandaganda, Mngeni Valley, Natal, South Africa. S. Afr. Hum. 1993 Oct 1;5(10):83-98.
194. Fraser LM, Badenhorst S. Livestock use in the Limpopo Valley of southern Africa during the Iron Age. S. Afr. Archaeol. Bull. 2014 Dec 1:192-8.
195. Manyanga M, Pikirayi I, Ndoro W. Coping with dryland environments: preliminary results from Mapungubwe and Zimbabwe phase sites in the Mateke Hills, south-eastern Zimbabwe. S. Afr. Archaeol. Soc.: Goodwin Series. 2000 Dec 1:69-77.
196. Vogel JO. Kamangoza: An introduction to the Iron Age cultures of the Victoria Falls region. Oxford: Oxford University Press; 1971.
197. Plug I. Seven centuries of Iron Age traditions at Bosutswe, Botswana: a faunal perspective. South African Journal of Science. 1996 Feb 1;92(2):91-7.
198. Denbow J, Smith J, Ndobochani NM, Atwood K, Miller D. Archaeological excavations at Bosutswe, Botswana: cultural chronology, paleo-ecology and economy. J. Archaeol. Sci. 2008 Feb 1;35(2):459-80.
199. Denbow J. Excavations at Divuyu, Tsodilo Hills. Botswana Notes and Records. 2011 Jan 1;43:76-94.
200. Badenhorst S. Measuring change: cattle and caprines from Iron Age farming sites in southern Africa. S. Afr. Archaeol. Bull. 2011 Dec 1;66(194):167-72.
201. Kinahan J. The Acquisition of ceramics by hunter-gatherers on the Middle Zambezi in the first and second millennium AD. J. Afr. Archaeol. 2013 Nov 11;11(2):197-209.
202. Sinclair P. Chibuene—an early trading site in southern Mozambique. Paideuma. 1982 Jan 1:149-64.
203. Sinclair P, Ekblom A, Wood M. Trade and society on the south-east African coast in the later first millennium AD: the case of Chibuene. Antiquity. 2012 Sep;86(333):723-37.
204. Badenhorst S, Sinclair P, Ekblom A, Plug I. Faunal remains from Chibuene, an Iron Age coastal trading station in central Mozambique. S. Afr. Hum. 2011b Jan 1;23(1):1-5.
205. Van Rijssen WJ, Avery G. The late Holocene deposits at Klein Kliphuis Shelter, Cedarberg, Western Cape Province. S. Afr. Archaeol. Bull. 1992 Jun 1:34-43.
206. Orton J, Mackay A. New excavations at Klein Kliphuis rock shelter, Cederberg Mountains, Western Cape, South Africa: the late Holocene deposits. S. Afr. Archaeol. Bull. 2008;63(187):69.
207. Phillipson DW. Early Iron Age sites on the Zambian copperbelt. Azania. 1972 Jan 1;7(1):93-128. Available from <https://doi.org/10.1080/00672707209511558>
208. Binneman JNF, Webley L, Biggs V. Notes and reports: preliminary notes on an Early Iron Age site in the Great Kei River valley, Eastern Cape. Southern African Field Archaeology. 1992 Nov 1;1(2):108-9.
209. Deacon HJ. Two radiocarbon dates from Scott's Cave, Gamtoos valley. S. Afr. Archaeol. Bull. 1967 Sep 1;22(86):51-2
210. Klein RG, Scott K. Fauna of Scotts Cave, Gamtoos Valley, southeastern Cape-province. South African Journal of Science. 1974 Jan 1;70(6):186-7.
211. Sadr K. The Neolithic of southern Africa. J. Afr. Hist. 2003 Jul;44(2):195-209.
212. Maggs T. Ndondondwane: a preliminary report on an Early Iron Age site on the lower Tugela River. Annl. Natal Mus. 1984 Dec 1;26(1):71-93.
213. Voigt EA, Von den Driesch A. Preliminary report on the faunal assemblage from Ndondondwane, Natal. S. Afr. Hum. 1984;26(1):95-104.
214. Van Schalkwyk LO, Greenfield H, Jongsma T. The early Iron Age site of Ndondondwane, Kwazulu-Natal, South Africa: preliminary report on the 1995 excavations. Southern African Field Archaeology. 1997;6(2):61-77.
215. Fagan BM. Radiocarbon dates for sub-Saharan Africa—IV. J. Afr. Hist. 1966 Nov;7(3):495-506.
216. Ohinata F. Archaeology of iron-using farming communities in Swaziland: pots, people and life during the first and second millennia AD. Ph.D Thesis, Oxford University. 2002.
217. Wilmsen EN. Nqoma: an abridged review. Botswana Notes and Records. 2011 Jan 1;43:95-114.
218. Davies O. Excavations at Shongweni South Cave: the oldest evidence to date for cultigens in southern Africa. Annl. Natal Mus. 1975 Nov 1;22(2):627-62.
219. Inskeep RR, Avery G. Nelson Bay Cave, Cape Province, South Africa: the Holocene levels. Oxford: British Archaeological Reports International Series 357. 1987.
220. Bollong CA, Vogel JC, Jacobson L, van der Westhuizen WA, Sampson CG. Direct dating and identity of fibre temper in pre-contact Bushman (Basarwa) pottery. J. Archaeol. Sci.. 1993 Jan 1;20(1):41-55.
221. Hanisch EO. An archaeological interpretation of certain Iron Age sites in the Limpopo/Shashi Valley. Ph.D. Thesis, University of Pretoria. 1980.
222. Maggs MA, Michael MA. Ntshekane: an Early Iron Age site in the Tugela Basin, Natal. Annl. Natal Mus. 1976 Nov 1;22(3):705-40
223. Garlake PS. An Early Iron Age site near Tafuna Hill, Mashonaland. S. Afr. Archaeol. Bull. 1971 Dec 1;26(103/104):155-63.
224. Huffman TN. Tafuna hill. S. Afr. Archaeol. Bull. 1974 Jun 1;29(113/114):65-6.
225. Soper RC. New radiocarbon dates for eastern and southern Africa. J. Afr. Hist. 1974 Apr;15(2):175-92.
226. Huffman TN. Test excavations at NaBa and Lanlory, northern Mashonaland. S. Afr. Archaeol. Soc.: Goodwin Series. 1979 Jan 1(3):14-46.
227. Sandelowsky BH. Kapako and Vungu Vungu: Iron Age sites on the Kavango River. S. Afr. Archaeol. Soc.: Goodwin Series. 1979 Jan 1(3):52-61.
228. Beaumont PB, Boshier AK. Report on test excavations in a prehistoric pigment mine near Postmasburg, Northern Cape. S. Afr. Archaeol. Bull. 1974 Jun 1:41-59.
229. Fagan BM. Radiocarbon dates for sub-Saharan Africa: VI. J. Afr. Hist. 1969 Jan;10(1):149-69.
230. Phillipson DW. The prehistoric sequence at Nakapapula rockshelter, Zambia. Proceedings of the Prehistoric Society. 1970a Feb; 35:172-202.
231. Phillipson DW. Excavations at Twickenham Road, Lusaka. Azania. 1970b Jan 1;5(1):77-118. Available from <https://doi.org/10.1080/00672707009511529>
232. Robinson KS. The Nkhotakota Lake Shore and Marginal Areas, Malaŵi: an Archaeological Reconnaissance. Malawi: Malaŵi Government, Department of Antiquities, Ministry of Local Government; 1979.
