# Supplementary material for: The archaeological evidence for the appearance of pastoralism and farming in southern Africa
Source: PLoS One. 2018 Jun 14;13(6):e0198941. doi: 10.1371/journal.pone.0198941 (PMC6002040; doi:10.1371/journal.pone.0198941)
Supplement: S3 Table — (DOCX) [file pone.0198941.s011.docx]

Table S3. List of Archaeological sites that were excluded from Database 1.

| **Site ID** | **Site Name** | **Country** | **Hunter-gatherer, pastoralist or farmer site** | **Radiocarbon date** | **Lab number** | **Reason for exclusion** | **References** |
| --- | --- | --- | --- | --- | --- | --- | --- |
| HRS | Highlands Rockshelter | South Africa | Hunter-gatherer | 3570±50 | Pta-563 | iii | [1] |
| MEL | Melkhoutboom | South Africa | Hunter-gatherer | 2870±90 250±70 | Pta-706 Gak-1537 | iii | [1] |
| MAJ | Anne Shaw, Middledrift | South Africa | Pastoralist/Hunter-gatherer | 2850±50 | Pta-1030 | iii | [2] |
| RIVM | Riversmead | South Africa | Hunter-gatherer | 2645±95 | GX-0723 | iii | [3] |
| FAI | Fairview | South Africa | Hunter-gatherer | 2450±55 | Pta-2587 | iii | [4] |
| WIL | Wilton | South Africa | Hunter-gatherer | 2270±100 | Gak-1540 | i | [5] |
| STBF | Steenbokfontein | South Africa | Hunter-gatherer | 2360±40  2200±50  2200±60 | Pta-6136 Pta-6424 Pta-6136 | iii | [6] |
| CAL | Calder's Cave | Zimbabwe | Farmer | 1970±80 | UCLA-929 | ii | [7] |
| FURI | Furi I Mine | Angola | Farmer | 1800±80 | UCLA-170 | ii | [8] |
| MUN | Munga Hill Site | Malawi | Farmer | 1750±60 | Pta-2752 | i | [9] |
| MAD2 | Madede 2 | Malawi | Farmer | 1680±50 | Pta-2943 | i | [9] |
| LANG | Langubhela | Swaziland | Farmer | 1620±50 | Pta-3346 | i | [10] |
| KAN | Kansanshi Hill | Zambia | Farmer | 1550±90 1320±85 1240±90 1190±85 | N-1289 N-1283 N-1607 N-1284 | i | [11, 12] |
| MAD1 | Madede 1 | Malawi | Farmer | 1470±50 | Pta-2740 | i | [9] |
| DIAN | Diana's Vow | Zimbabwe | Farmer | 1440±50 | Pta-2001 | i | [10] |
| KAMB | Kambari | Malawi | Farmer | 1410±50 | Pta-2744 | i | [10] |
| KINS | Kinsale Farm | Zimbabwe | Farmer | 1410±95 | SR-117 | ii | [13] |
| STS | Strauss | South Africa | Farmer | 1410±110 1390±70 | Wits-1531 Wits-1501 | ii | [14] |
| FIC | Ficus | South Africa | Farmer | 1400±40 | Wits-782 | Discovered too late for inclusion in this analysis | [14,15] |
| MUC | Mucuquesa 3 Mine | Angola | Farmer | 1330±80 | UCLA-711 | i | [8] |
| SINO | Sinoia Caves | Zimbabwe | Farmer | 1300±95 | SR-118 | i | [13] |
| MIL | Millbank | South Africa | Farmer | 1290±50 1250±50 1200±50 1190±50 1190±45 1170±40 1090±50 | Pta 6309 Pta 7468 Pta 6306 Pta 6307 Pta 7480 Pta 7478 Pta 6205 | Discovered too late for inclusion in this analysis | [16] |
| NHA | Nhachengue | Mozambique | Farmer | 1254±80 | ST-8497 | i | [17] |
| FET | Feti la Choya | Angola | Farmer | 1240±100 | Y-1240 | i | [18] |
| BAS | Basanga | Zambia | Farmer | 1220±120 1160±115 | N-595 N-594 | i | [19] |
| DUNA | Dundo Airfield | Angola | Farmer | 1190±80 | UCLA-716 | i | [8] |
| DZ13 | DZ13 | Malawi | Farmer | 1180±30 1150±70 | Pta-2028 HAR-2400 | ii | [9] |
| KLING | Klingbeil | South Africa | Farmer | 1160±50 1120±50 1000±40 | Pta-1633 Pta-2160 Pta-1747 | ii | [20] |
| BEU | Beauley | South Africa | Farmer | 1135±50 1120±50 | Pta-5951 Pta-5948 | ii | [14] |
| PITS | Pitsane | Botswana | Farmer | 1120±60 980±40 | Pta-1817 Pta-1957 | i | [20] |
| HOL | Hola Hola | Mozambique | Farmer | 1060±50 | R_1326 | i | [17] |
| MSIN | Massingir 1/72 | Mozambique | Farmer | 1030±40 | Pta-1640 | i | [17] |
| XAI | Xai-Xai | Mozambique | Farmer | 510±165 | St-8589 | ii | [17] |

References:

1. Deacon J. Where hunters gathered: a study of Holocene Stone Age people in the Eastern Cape. Claremont: South Africa Archaeological Society, 1976.
2. Derricourt RM. Prehistoric man in the Ciskei & Transkei. Cape Town: C. Struik; 1977**.**
3. Sampson G, Sampson M. Riversmead Shelter: excavations and analysis. Annls. Natal Mus. 1967.
4. Robertshaw PT.Excavations at Fairview rock shelter – a contribution to the prehistory of the Eastern Cape Province of South Africa. Annls. Cape Prov. Mus. 1982; 1 (2): 55–92.
5. Deacon J. Wilton: an assessment after fifty years. S. Afr. Archaeol. Bull. 1972 27(105/106):10-48.
6. Jerardino A, Yates R. Preliminary results from excavations at Steenbokfontein Cave: implications for past and future research. S. Afr. Archaeol. Bull. 1996 1:7-16.
7. Fagan BM. Radiocarbon dates for sub-Saharan Africa—IV. J. Afr. Hist. 1966 7(3):495-506.
8. De Maret P, van Noten F, Cahen D. Radiocarbon dates from west central Africa: a synthesis. J. Afr. Hist. 1977 18(4):481-505.
9. Mgomezulu GG. Recent archaeological research and radiocarbon dates from Eastern Africa. J. Afr. Hist. 1981 22(4):435-56.
10. Vogel JC, Fuls A, Visser E. Pretoria radiocarbon dates III. Radiocarbon. 1986 28(3):1133-72.
11. Soper RC. New radiocarbon dates for eastern and southern Africa. J. Afr. Hist. 1974 15(2):175-92.
12. Maggs T. Some recent radiocarbon dates from eastern and southern Africa. J. Afr. Hist. 1977 18(2):161-91.
13. Fagan BM. Radiocarbon dates for sub-Saharan Africa: VI. J. Afr. Hist. 1969 10(1):149-69.
14. Huffman TN. Handbook to the Iron Age: the archaeology of pre-colonial farming societies in southern Africa. KwaZulu-Natal: University of KwaZulu-Natal Press; 2007.
15. Partridge TC. Ficus Cave: an Iron Age living site in the central Transvaal. S. Afr. Archaeol. Bull. 1966 21(83):125-32.
16. Van Schalkwyk JA. Excavation of a Late Iron Age site in the Makgabeng, Northern Province. S. Afr. Field Archaeol. 2000 9:75-82.
17. Morais J. The early farming communities of southern Mozambique. Sweden: Central Board of National Antiquities; 1988.
18. Fagan BM. Radiocarbon Dates for Sub-Saharan Africa (from c. 1000 bc)—III. J. Afr. Hist. 1965 6(1):107-16.
19. Yamasaki F, Hamada T, Hamada C. Riken natural radiocarbon measurements VI. Radiocarbon. 1970 12(2):559-76.
20. Hall M, Vogel JC. Some recent radiocarbon dates from southern Africa. J. Afr. Hist. 1980 21(4):431-55.
