# Supplementary material for: The archaeological evidence for the appearance of pastoralism and farming in southern Africa
Source: PLoS One. 2018 Jun 14;13(6):e0198941. doi: 10.1371/journal.pone.0198941 (PMC6002040; doi:10.1371/journal.pone.0198941)
Supplement: S1 Text — (DOCX) [file pone.0198941.s012.docx]

Journals and reports consulted for the compilation of Database 1

1. African Archaeological Review
2. African Studies
3. American Anthropology
4. Annals of the Ditsong National Museum of Natural History
5. Annals of the Eastern Cape Museum
6. Annals of the South African Museum
7. Antiquity
8. Azania: Archaeological Research in Africa
9. Botswana Notes and Records
10. Ciencias Biologicas
11. Cimbebasia
12. Current Anthropology
13. Department of Water Affairs, Botswana (unpublished reports)
14. Eduardo Mondlane University Mozambique, Central Board of National Antiquities, Sweden
15. Journal of African Archaeology
16. Journal of African History
17. Journal of Archaeological Science
18. Journal of Arid Environments
19. Madoqua
20. Malawi, Ministry of Local Government, Department of Antiquities (published reports)
21. Man
22. Navorsinge van die Nasionale Museum
23. Nyame Akuma
24. Paideuma
25. PlosOne
26. Quaternary International
27. Radiocarbon
28. Revista Portuguesa de Arquelogia
29. Society of Malawi
30. Southern African Field Archaeology
31. South African Journal of Science
32. Southern African Humanities (formerly known as Annals of the Natal Museum and Natal Museum Journal of Humanities)
33. The South African Archaeological Bulletin
34. The South African Archaeological Society, Goodwin Series
