# Supplementary material for: The archaeological evidence for the appearance of pastoralism and farming in southern Africa
Source: PLoS One. 2018 Jun 14;13(6):e0198941. doi: 10.1371/journal.pone.0198941 (PMC6002040; doi:10.1371/journal.pone.0198941)
Supplement: S2 Text — (DOCX) [file pone.0198941.s013.docx]

Supplementary Text 2: The full electronic search strategy using an example.

**Step 1**: Ebsco Host Search database as accessed through web.b.ebscohost.com

The following Ebsco Host databases were consulted: Academic Search Complete and Africa-Wide Information. In this example we use Academic Search Complete.

.

**Step 2**: Enter the three search terms: ‘Archaeology’, country (e.g. ‘Botswana’), and additional identifier (e.g. ‘Iron Age’).

Set the following parameters:

1. Search Modes: Find all my search terms
2. Limit your results: Published date: January 1950 to December 2016
3. Special limiters for Academic Search Complete:

Publication type: all

Document type: all

Language: all

Special limiters for Africa-Wide Information:

Publication Type: all

Document type: all

Language: all

Data Contributor: all

**Step 3**:

In all the electronic database searches the total number of search results (i.e. published material) retrieved were inflated.

We excluded results that were:

(1) Related to other countries.

(2) Unrelated to the key terms of search.

(3) Too general.

(4) Not confined to a country but instead covered extensive geographic areas.

(5) Not relevant for the time period.

(6) Rock art publications. This is because we were interested in excavated sites.

In this example, in searching with the terms: ‘Iron Age’, ‘Botswana’ and ‘Archaeology, 58 results were returned.

These search results were read and many excluded (see points 1 to 6 above). In this way, the total number of search results was reduced. In this example, after filtering, a total of 42 publications remained relevant to this study. These publications were then read to determine their geographical focus. In publications where archaeological sites were referenced, the geographic coordinates of these sites were used for the mapping exercise. When larger areas were referenced, the midpoint of the area was estimated on Google Earth Pro. The total number of publications per geographic coordinate set was then counted.
