# Supplementary material for: The archaeological evidence for the appearance of pastoralism and farming in southern Africa
Source: PLoS One. 2018 Jun 14;13(6):e0198941. doi: 10.1371/journal.pone.0198941 (PMC6002040; doi:10.1371/journal.pone.0198941)
Supplement: S3 Text — (DOCX) [file pone.0198941.s014.docx]

Supplementary Text 1

A summary for the chronological appearance of livestock and directly dated pottery at southern African sites for the period 550 BC to AD 1056.

**Key**:

Black font refers to LSA sites; red font refers to farmer sites; green font refers to fibre and grit tempered pottery sites and purple font refers to Iron working sites. The text can be read in conjunction with Figures 1 to 8 and figures S1- S8.

**Group 1: 551 – 351 BC**

**Site ID and Site Name** **Database Code** **Livestock (conventional and AMS) and Direct date for pottery**

**LPC** Leopard Cave 3 sheep AMS

**ZIT**  Zitundo 1 carbon dated pottery

**UNC**  University Campus 1 carbon dated pottery

**Group 2: 350 – 150 BC**

**Site ID and Site Name** **Database Code** **Livestock (conventional and AMS) and Direct date for pottery**

**LPC** Leopard Cave 3 sheep AMS

**FAC** Fackeltrager 1 -

**BBA** Bambata A 4 sheep/goat

**CLK** Clarke’s Shelter 1 -

**UNID** Uniondale 1 -

**SPR** Spoegrivier 2 sheep AMS

**MES** Messum 1 5i -

**BOU** Boundary 1 fibre from pottery

**ZIT** Zitundo 1 carbon dated pottery

**UNC** University Campus 1 carbon dated pottery

**CHG2** Changalane 2 1 carbon dated pottery

**SIT** Situmpa 1 -

**Group 3: 149BC – AD 51**

**Site ID and Site Name Database Code** **Livestock (conventional and AMS) and Direct date for pottery**

**LPC** Leopard Cave 3 sheep AMS

**BBA** Bambata A 4 sheep/goat

**CLK** Clarke’s Shelter 1 -

**UNID** Uniondale 1 -

**SPR** Spoegrivier 2 sheep AMS

**MES** Messum 1 5i -

**FSR** Falls Rock Shelter 3 sheep

**ORU** Orunwanje 95/1 3 sheep/goat?

**N2005/2** 1 -

**TOT1** Toteng 1 3 sheep AMS and cattle AMS

**AIT** Ai Tomas 3i sheep/goat

**BOR** Border Cave 1 -

**DK1** Die Kelders 1 -

**OBP** Olieboomspoort 1 -

**BLM** Blombos 3 sheep AMS

**APL** Apollo Cave 1 -

**BOU** Boundary Shelter 1 fibre from pot

**HAA** Haaskraal 1 fibre from pot

**ZIT** Zitundo 1 carbon dated pottery

**UNC** University Campus 1 carbon dated pottery

**CHG2** Changalane 2 1 carbon dated pottery

**SIT** Situmpa 1 -

**Group 4: AD 52 – 252**

**Site ID and Site Name Database Code** **Livestock (conventional and AMS) and Direct date for pottery**

**FSR** Falls Rock Shelter 3 sheep

**TOT1** Toteng 1 3 sheep AMS and cattle AMS

**AIT** Ai Tomas 3 sheep/goat

**BOR** Border Cave 1 -

**DK1** Die Kelders 1 -

**OBP** Olieboomspoort 1 -

**BLM** Blombos 3 sheep AMS

**APL** Apollo Cave 1 -

**OMU** Omungunda 99/1 1 -

**REN** Renbaan 1 -

**BUZ** Buzz Shelter 2 sheep

**DEP** Depression Cave 1 -

**HAW** Hawston 3 sheep

**KBA** Kasteelberg A 3 sheep

**COL** Colwinton 1 -

**SKE** Skeurkrans 1 -

**N2** N2000/2 1 -

**SNR** Snake Rock 1 -

**EGH** Edgehill 1 -

**ORU** Orunwanje 95/1 1 -

**SPR** Spoegrivier 2 sheep AMS

**REC** Reception Shelter 1 -

**GED** Geduld 3 sheep/goat?

**BLY** Blydefontein 1 -

**TOR** Tortoise Cave 1 -

**BLO5** Bloubos 5 1 -

**JKKM** Jakkalsberg M 3 sheep and sheep/goat

**CAE** Cae Cae 1 -

**KBDe** Kasteelberg De 3 sheep

**DRL** Driel Shelter 1 -

**JUB** Jubilee Shelter 1 -

**HAA** Haaskraal 1 fibre from pot

**VOL** Volstruisfontein 1 fibre from pot

**SIT** Situmpa 1 -

**CHG1** Changalane 1 1 carbon dated pottery

**MAT** Matola IV 6 -

**ZIT** Zitundo 6 -

**RIA** Rian Rock Shelter 5 -

**PHO** Phopo Hill 6i -

**LUM** Lumbi 1 -

**BEF** Benfica 1 -

**UNC** University Campus 6i -

**MTGL** Mitongwe/Liwadzi 1 -

**KAMU** Kamukombe 1 -

**SIL** Silver Leaves 7i -

**MTT** M’teteshi 5 -

**Group 5: AD 253 – 453**

**Site ID and Site Name Database Code Livestock (conventional and AMS) and Direct date for pottery**

**DEP** Depression Cave 1 -

**HAW** Hawston 3 sheep

**KBA** Kasteelberg A 3 sheep AMS

**SNR** Snake Rock Shelter 3i sheep

**EGH** Edgehill 1 -

**TOT1** Toteng 1 3 sheep and cattle AMS and carbon dated pottery

**BLY** Blydefontein 1 -

**TOR** Tortoise Cave 3 sheep

**BLO5** Bloubos 5 1 -

**JKKM** Jakkalsberg M 3 sheep and sheep/goat

**CAE** Cae Cae 1 -

**KBDe** Kasteelberg De 3 sheep

**JUB** Jubilee Shelter 1 -

**DRL** Driel Shelter 1 -

**ERO** Eros 1 -

**SHH** Sehonghong 1 -

**ORU** Orunwanje 95/1 3 goat

**DUI** Duiker Eiland 1i -

**OMU** Omungunda 99/1 5 -

**OBP** Olieboomspoort 1 -

**SKE** Skeurkrans 1 -

**DIK** Dikbosch 3 sheep/goat?

**LMR** Limerock Shelter 3ii sheep/goat ?(byre not directly associated to date)

**CLH** Collingham Shelter 1 -

**GED** Geduld 3 sheep/goat?

**LOT** Lotshitshi 3 cattle

**PBA** Pearly Beach Area 3i sheep

**NAU** Nauga 1 -

**KBE** Kasteelberg E 1 -

**APL** Apollo Cave 1 -

**DK1** Die Kelders 1 -

**BPS** Boomplaas 4i sheep

**VSS** Vonk se Stal 1 -

**KSBD** !Khuseb Delta 1 -

**KN41** KN2005/41 2 cattle AMS

**BOK** Bokvasmaak 1 -

**KN54** KN2005/54 1 -

**CLK** Clarke’s Shelter 1 -

**HAA** Haaskraal 1 fibre from pot

**SIT** Situmpa 1 -

**ZIT** Zitundo 6 -

**PHO** Phopo Hill 6i -

**BEF** Benfica 1 -

**UNC** University Campus 6i -

**MTGL** Mitongwe/Liwadzi 1 -

**MAT** Matola IV 6 -

**KAMU** Kamukombe 1 -

**SIL** Silver Leaves 7i -

**MAL** Malessane 1 -

**LUS** Lusi 1 -

**SMF** Samfya Forest 6i -

**EIL** Eiland Salt Works 1i -

**CHO** Chowo River 7i -

**MZJ** Mzonjani 6i -

**MAU** Maunatlala 7i -

**KAM** Kamnama 6i -

**GZP1** Great Zimbabwe 1i -

**NKP** Nkope 9 cow tooth

**ENK** Enkwazini 1i -

**RIA** Rian Rock Shelter 5 -

**KLA** Klein Afrika 9 cattle and goat

**NAMB** Namaso Bay 6i -

**HPR** Happy Rest 9i sheep, goat, sheep/goat and cattle

**KLF** Kalambo Falls 6i -

**IND** Inanda Quarry 6i -

**CHD** Chondwe 1 -

**CIG** Cigwa 5 -

**RUU2** Ruuga 2 6 - charcoal from pottery

**Group 6: AD 454 – 654**

**Site ID and Site Name Database Code Livestock (conventional and AMS) and Direct date for pottery**

**TOR** Tortoise Cave 3 sheep

**LOT** Lotshitshi 3 cattle

**PBA** Pearly Beach Area 3i sheep

**NAU** Nauga 1 -

**KBE** Kasteelberg E 1 -

**APL** Apollo Cave 1 -

**SNR** Snake Rock Shelter 3i sheep

**DK1** Die Kelders 1 -

**KBA** Kasteelberg A 3 sheep AMS

**BPS** Boomplaas 4i sheep

**VSS** Vonk se Stahl 1 -

**TOT1** Toteng 1 3 sheep and cattle AMS

**OBP** Olieboomspoort 1 -

**KSBD** !Khuseb Delta 1 -

**OMU** Omungunda 99/1 5 -

**KN54** KN2005/54 1 -

**CLK** Clarke’s Shelter 1 -

**BJP** Biesje Poort 1 -

**DIK** Dikbosch 3 sheep/goat?

**KBJ** Kabeljous 3 sheep

**KN12** KN2004/012 1 -

**MIR** Mirabib 2 sheep hair and dung layer

**JKKB** Jakkalsberg B 4 sheep

**WIT** Witklip 3 sheep?

**MES** Messum 1 5 -

**BYN** Bynekranskop 3 sheep AMS

**JUB** Jubilee 1 -

**OVI** Ovizorombuku 96/1 1 -

**BLY** Blydefontein 1 -

**DK1** Die Kelders 1 -

**SPR** Spoegrivier 3 sheep AMS

**JP7** Jagt Pan 7 1 -

**HAR** Hartman Valley 1 -

**BES** Big Elephant Shelter 1 -

**ORU** Orunwanje 95/1 1 -

**KN41** KN2005/41 2 cattle AMS

**BOK** Bokvasmaak 1 -

**KWK** Klein Witkrans 1 -

**LMR** Limerock 3ii sheep/goat? (byre not directly associated to date)

**HAA** Haaskraal 1 fibre from pot

**ZIT** Zitundo 6 -

**MAV** Mabveni 9i cattle and sheep/goat

**PHO** Phopo 6i -

**MAT** Matola IV 6 -

**SIT** Situmpa 1 -

**SIL** Silver Leaves 7i -

**SMF** Samfya Forest 6i -

**EIL** Eiland Salt Works 1i -

**CHO** Chowo River 7i -

**MZJ** Mzonjani 6i -

**MAU** Maunatlala 7i -

**KAM** Kamnama 6i -

**GZP1a** Great Zimbabwe 1i -

**NKP** Nkope Bay 9 cow tooth

**ENK** Enkwazini 1i -

**RIA** Rian Rock Shelter 5 -

**KLA** Klein Afrika 9 cattle and goat

**UNC** University Campus 6i -

**NAMB** Namaso Bay 5i -

**HPR** Happy Rest 9i cattle, sheep/goat, sheep and goat

**KLF** Kalambo Falls 6i -

**IND** Inanda Quarry 6i -

**CHD** Chondwe 1 -

**CIG** Cigwa 5 -

**SAM** Samakande 6i -

**174** 2931 CA174 1 -

**GUN** Gundu 1i -

**KLD** Kalundu Mound 8i cattle and sheep/goat

**KAD** Kadzi 8 sheep, sheep/goat, goat (c.f.) and cattle

**MON** Mondake 7 -

**BAN** Banda 6i -

**KGG** KwaGandaganda 9ii sheep, goat, sheep/goat, cattle and livestock byre

**NMK** Namkala 5 -

**MAM** Mamba 9ii sheep, goat, sheep/goat, cattle and livestock byre

**MGR** Magarape 7i -

**WOS** Wosi 9 sheep, sheep/goat, goat and cattle

**LUM** Lumbi 6/9 sheep? and cattle?

**SIM** Sioma Mission 6 -

**CHA** Chalaka 1 -

**LON** Lonze Forest 1 -

**RIV** Riverside 3ii cattle

**KAMU** Kamukombe 3 sheep/goat

**GOK** Gokomere 6i -

**LHC** Leopard Hill Cave 1 -

**KMZ** Kumadzulo 9i cattle

**KAB** Kabondo Kambo 6i -

**MLP** Mhlopeni 9i sheep and sheep/goat

**BRD** Broederstroom 8i sheep/goat and single cow bone

**Ma38** Ma38 3 sheep/goat

**KPW** Kapwirimbwe 9i cattle

**CAC** Castle Cavern 6 -

**LYD** Lydenburg Head 9i sheep, sheep/goat and cattle

**LMH** Lumbule Hill 6i -

**KWC** Kwali Camp 9i sheep and cattle

**MSC** Msuluzi Confluence 9i sheep, sheep/goat and cattle

**MAK** Makwe 4i cattle and goat

**Tsh1** Tsh1 3 sheep/goat

**KWC** Kwali Camp 9i sheep and cattle

**RUU2** Ruuga 6 charcoal from pottery

**KAP** Kapako 1 surface find: charcoal extracted from pottery

**Group 7: AD 655 – 855**

**Site ID and Site name Database Code Livestock (conventional and AMS) and Direct date for pottery**

**LMR** Limerock 3ii sheep/goat? (byre not directly associated to date)

**JKKB** Jakkalsberg B 4 sheep

**WIT** Witklip 3 sheep

**MES** Messum 1 5 -

**BYN** Byneskranskop 3 sheep AMS

**BES** Big Elephant Shelter 1 -

**HAR** Hartman Valley 1 -

**JP7** Jagt Pan 7 1 -

**OVI** Ovizorombuku 96/1 1 -

**DK1** Die Kelders 3 sheep AMS

**SPR** Spoegrivier 3 sheep AMS

**APL** Apollo Cave 1 -

**KBE** Kasteelberg E 1 -

**WEL** Welgeluk 1 -

**JKKA** Jakkalsberg A 3 sheep

**SBC** Steenberg Cove 3 sheep

**OBP** Olieboomspoort 3 sheep/goat

**LIK** Likoaeng 2 sheep AMS

**BLNM** Balerno Main 1 -

**ORU** Orunwanje 95/1 3 goat

**BLY** Blydefontein 1 -

**RSF** Roosfontein 1 -

**KBC** Kasteelberg C 3 sheep

**AB** Atlantic Beach 3i sheep

**SCO** Scott’s Cave 2 sheep

**BJP** Biesje Poort 1 -

**WON** Wonderwerk 3 sheep/goat?

**HAA** Haaskraal 1 fibre from pot

**BIS** Bisoli 3 livestock present

**MPM** Mpame 3 cattle?

**TMB** Tambala 7i -

**MAG** Magogo 8i sheep, sheep/goat and cattle

**PLT** Plaston 1 -

**CHO** Chowo River 6i -

**CHU** Chundu 4 cattle

**LEB** Lebalelo 3i sheep/goat

**LPK** Leopard’s Kopje 8ii sheep/goat, cattle and livestock byre

**TAU** Taukome 3ii sheep/goat, cattle and livestock byre

**MATL** Matlapaneng 8 sheep/goat, cattle and livestock byre

**NTS** Ntsitsana 9ii livestock present based on the presence of a byre

**KAD** Kadzi 8 sheep, goat (c.f.) and cattle

**NAN** Nanda 9i sheep, sheep/goat, goat and cattle

**NGA** Nanga 3 sheep/goat and cattle

**MWE** Mwenezi 2 sheep and cattle

**KMG** Kamangoza 5i -

**ZMF** Zambezi Farm 4i cattle

**BOS** Bosutswe 4ii sheep, sheep/goat, goat, cattle and livestock byre

**DIV** Divuyu 9i sheep, sheep/goat, goat and cattle

**IGI** Ingombe Ilede 8i sheep/goat and cattle

**KNO** Kanono Mulapo 1 dated charcoal from pottery

**CHB** Chibuene 3 sheep, sheep/goat, goat and cattle

**BRD** Broaderstroom 8ii sheep/goat and cattle by association to a byre

**KUL** Kulubele 1i -

**MGR** Magarape 7i -

**NDD** Ndondondwane 8ii sheep, sheep/goat, goat, cattle and livestock byre

**KANG** Kangonga 6i -

**SK17** SK17 3 sheep/goat and cattle

**QOG** Qogana 1i -

**WOS** Wosi 9i sheep, sheep/goat, goat and cattle

**KLD** Kalundu Mound 8i sheep/goat and cattle

**KLF** Kalambo Falls 6i -

**DC19** 2531DC19 1 OSL on pottery

**DAM** Dambwa 6i -

**FIB** Fibobe 5i -

**NQM** Nqoma 4i sheep, sheep/goat, goat and cattle

**KGG** KwaGandaganda 9ii sheep, sheep/goat, goat, cattle and livestock byre

**RIA** Rian Rock Shelter 5 -

**CHD** Chondwe 6i -

**174** 2931 CA174 1 -

**GUN** Gundu 1i -

**PHO** Phopo Hill 6i -

**NAMB** Namaso Bay 5i -

**KLA**  Klein Afrika 9i sheep/goat and cattle

**LONF** Lonze Forest 1 -

**MAT** Matola IV 6 -

**GOK** Gokomere 6i -

**LHC** Leopard Hill Cave 1 -

**UNC** University Campus 6i -

**NAM** Namkala 5 -

**MAM** Mamba 9ii sheep, sheep/goat, goat, cattle and livestock byre

**MSC** Msuluzi Confluence 9i sheep, sheep/goat, goat and cattle

**BAN** Banda Hill 6i -

**LMH** Lumbule Hill 6i -

**KAB** Kabondo Kumbo 6i -

**LYD** Lydenburg Head 9i sheep, sheep/goat and cattle

**Tsh1** Tsh1 3 sheep/goat

**SIM** Sioma Mission 6 -

**DIA** Diamant 3ii sheep/goat, cattle and livestock byre

**RUU2** Ruuga 2(39) 6 -

**Group 8: AD 856 – 1056**

**Site ID and Site Name Database Code Livestock (conventional and AMS) and Direct date for pottery**

**BJP** Biesje Poort 1 -

**AUS** Australitz 1 -

**BYL** Blydefontein 1 -

**KN15** KN2004/015E 1 -

**KFB** Kreeftebaai 1 -

**KBA** Kasteelberg A 3 sheep and cattle

**URU** Ururu 1 -

**AB** Atlantic Beach 3 sheep

**DSM** Drie Susters Main 3 sheep/goat and cattle

**OVI** Ovizorombuku 96/1 1 -

**BES** Big Elephant Shelter 3 sheep and cattle?

**DOF** Doornfontein 1 -

**KBB** Kasteelberg B 3 sheep and cattle

**TOT3** Toteng 3 2 sheep AMS

**OBP** Olieboomspoort 3 sheep/goat?

**BKK** Blinkklipkop 3 sheep/goat?

**KSBD** !Khuseb Delta 1 -

**NBC** Nelson Bay Cave 3 sheep AMS

**ORU** Orunwanje 95/1 3 sheep and goat

**EBC** Elands Bay Cave 3 sheep

**SPR** Spoegrivier 3 sheep AMS

**KBC** Kasteelberg C 3 sheep

**RSF** Roosfontein 1 -

**GNE** Glen Elliot 1 fibre from pot

**ZAY** Zaayfontein 1 fibre from pot

**KKH** Klein Kliphuis 3 sheep

**CAE** Cae Cae 9 cow

**NAMB** Namaso Bay 5i -

**UNC** University Campus 6i -

**MPM** Mpame 3 cattle?

**NDN** Ndonde 1i -

**CHI** Chia Lagoon 1 -

**TWK** Twickenham Road 9i goat horn

**NPL** Nakapapula 1 -

**ISP** Isamu Pati 9i sheep/goat and cattle

**GUN** Gundu 1i -

**ZMF** Zambezi Farm 4i cattle

**KNO** Kanono Mulapa 1 charcoal from pot

**KAB** Kabondo Kumbo 6i -

**KMG** Kamangoza 5i -

**CHO** Chowo River 10 -

**RVS** Riverside 3ii cattle, livestock byre

**CHB** Chibuene 3 sheep, sheep/goat, goat and cattle

**LUB** Lubusi 6 -

**KLD** Kalundu Mound 8i sheep/goat and cattle

**CHD** Chondwe 6i -

**KGG** KwaGandaganda 9i sheep, sheep/goat, goat and cattle

**PTD** Pont Drift 3 sheep/goat and cattle

**Le7a** Le7a 3 sheep/goat and cattle

**KAD** Kadzi 8 sheep, sheep/goat, goat (c.f.) and cattle

**LRY** Lanlory 6i -

**THD** Thandwe 1 -

**TAF** Tafuna Hill 6i -

**NTKE** Ntshekane 9i sheep, sheep/goat and cattle

**SCH** Schroda 8i sheep/goat and cattle

**Le6** Le6 3 sheep/goat and cattle

**SHO** Shongweni South 10 -

**BOS** Bosutswe 4ii sheep, sheep/goat, goat, cattle and livestock byre

**NQM** Nqoma 8i sheep, sheep/goat and cattle

**KZ1** Kazindu 6 -

**MAM** Mamba 9ii sheep, sheep/goat, goat, cattle and livestock byre

**NDD** Ndondondwane 8ii sheep, sheep/goat, goat, cattle and livestock byre

**MAG** Magogo 8i sheep, sheep/goat and cattle

**DOM** Dombashaba 1 -

**MWE** Mwenezi 2 sheep, sheep/goat, goat and cattle

**NGA** Nanga 3 sheep/goat and cattle

**NAN** Nanda 9i sheep, sheep/goat, goat and cattle

**MATL** Matlapaneng 8 sheep/goat and cattle

**TAU** Taukome 3ii sheep/goat, cattle and livestock byre

**LPK** Leopards Kopje 8ii sheep/goat, cattle and livestock byre

**LEB** Lebabelo 3i sheep/goat

**CHU** Chundu 4 cattle

**PLT** Plaston 1 -

**SK17**  SK17 3 sheep/goat and cattle

**DIA** Diamant 3ii sheep/goat, cattle and livestock byre

**IGI** Ingombe Ilede 8i sheep/goat and cattle

**KAP** Kapako 6i -
